# Cardiovascular Risk in Pancreatic Cancer: A Meta-Analysis of 197 Studies

**DOI:** 10.3390/cancers18071108

**Published:** 2026-03-29

**Authors:** Jázmin Németh, Jimin Lee, Orsolya Eperjesi, Endre Botond Gagyi, Zoltán Bánfalvi, Veronika Lillik, Ioana Creanga-Murariu, Réka Tóth, Eszter Ágnes Szalai, Mahmoud Obeidat, Szilárd Váncsa, Stefania Bunduc, Péter Hegyi

**Affiliations:** 1 Centre for Translational Medicine, Semmelweis University, 1085 Budapest, Hungary; nemethjazmin222@gmail.com (J.N.); jmnlee10@gmail.com (J.L.); eperjesiorsi1348@gmail.com (O.E.); endre.gg@gmail.com (E.B.G.); banfalvi.zoltan11@gmail.com (Z.B.); ioana.creanga@d.umfiasi.ro (I.C.-M.); rekatoth83@gmail.com (R.T.); obeidat.mahmoud96@gmail.com (M.O.); vancsa.szilard@gmail.com (S.V.); hegyi2009@gmail.com (P.H.); 2 Institute for Translational Medicine, Medical School, University of Pécs, 7622 Pécs, Hungary; 3 Department of Internal Medicine, Toldy Ferenc Hospital, 2700 Cegléd, Hungary; 4 Institute of Pancreatic Diseases, Semmelweis University, 1085 Budapest, Hungary; 5 Department of Medical Imaging, Bajcsy-Zsilinszky Hospital and Clinic, 1106 Budapest, Hungary; 6 Selye János Doctoral College for Advanced Studies, Semmelweis University, 1085 Budapest, Hungary; 7 Fejér County Szent György University Teaching Hospital, 8000 Székesfehérvár, Hungary; 8 Advanced Research and Development Center for Experimental Medicine (CEMEX), “Grigore T. Popa” University of Medicine and Pharmacy, 700454 Iasi, Romania; 9 Department of Restorative Dentistry and Endodontics, Semmelweis University, 1085 Budapest, Hungary; 10 Carol Davila University of Medicine and Pharmacy, 020021 Bucharest, Romania; 11 Digestive Disease and Liver Transplant Centre, Fundeni Clinical Institute, 022328 Bucharest, Romania; 12 Translational Pancreatology Research Group, Interdisciplinary Centre of Excellence for Research Development and Innovation, University of Szeged, 6720 Szeged, Hungary

**Keywords:** pancreatic cancer, cardio-vascular events, thromboembolism, meta-analysis

## Abstract

**Background/Objectives**: Cardiovascular diseases (CVDs) frequently limit the feasibility and effectiveness of cancer treatment. However, CVD burden has not been comprehensively described in pancreatic cancer. In our systematic review and meta-analysis, we evaluated the prevalence and incidence of CVDs in pancreatic ductal adenocarcinoma (PDAC). **Methods**: We conducted the systematic search in PubMed, EMBASE, and CENTRAL on 5 February 2024. Studies reporting the prevalence or incidence of CVDs in PDAC were included. Subgroup analyses were performed based on cancer stage and treatment type. Pooled proportions with the 95% confidence interval (CI) were calculated using a random-effects model. (PROSPERO: CRD42023482295). **Results**: We included 197 articles. At PDAC diagnosis, non-thrombotic cardiovascular diseases (NT-CVDs) were as prevalent as in the general population: hypertension in 33% (CI: 27–40%), ischemic heart disease in 6% (CI: 3–12%), and heart failure, arrhythmia, and stroke each in 2–3%. Their incidence during treatment remained low (1–10%). Thrombotic events, excluding pulmonary embolism, were present in 11% (CI: 7–15%) at diagnosis, with an incidence of 8–10% regardless of stage or treatment. Pulmonary embolism affected 3% at diagnosis and occurred at a similar rate during treatment. **Conclusions**: Thromboembolic events are common in PDAC and occur both at diagnosis and during follow-up. Their incidence remains stable across treatment modalities and disease stages, suggesting that the tumor itself is the primary driver of thrombotic risk. The prevalence of NT-CVDs in PDAC is comparable to that in the general population and shows minimal variation across cancer stages or treatment modalities.

## Simple Summary

Cardiovascular diseases (CVDs) frequently complicate cancer management and may limit treatment options in pancreatic ductal adenocarcinoma (PDAC). However, their prevalence and incidence in PDAC have not been comprehensively described. This systematic review and meta-analysis aimed to evaluate the burden of CVDs in patients with PDAC. We conducted the systematic search in 3 main databases. Studies reporting the prevalence or incidence of CVDs in PDAC were included. Non-thrombotic cardiovascular diseases, including hypertension, ischemic heart disease, heart failure, arrhythmia, and stroke, were present at rates comparable to those of the general population and showed a low incidence during treatment. In contrast, thrombotic events were common at diagnosis and occurred at similar rates during follow-up, regardless of disease stage or treatment modality. These findings suggest that thrombotic risk in PDAC is primarily driven by the tumor itself, underlining the need for continuous vigilance from the time of diagnosis.

## 1. Introduction

Pancreatic cancer (PC) is associated with a great burden of comorbidities, of which cardiovascular diseases (CVDs) play a particularly significant role in influencing treatment strategies and overall prognosis. However, the incidence and prevalence of cardiovascular comorbidities in patients with PC remain poorly characterized [1,2,3].

Emerging evidence suggests that CVDs occur at elevated rates in this population, with several studies highlighting a complex, bidirectional association between PC and cardiovascular pathology [4,5,6,7,8]. This relationship may be partially explained by shared environmental risk factors and overlapping genetic predispositions—both inherited and acquired [9]. Moreover, many therapeutic modalities used in the management of PC, including chemotherapy, radiotherapy, small-molecule inhibitors, and monoclonal antibodies, are known to have cardiotoxic effects, further complicating the clinical landscape [9,10].

Cardiovascular complications associated with PC treatment can range from asymptomatic electrocardiographic abnormalities to clinically significant conditions such as pericarditis, myocardial infarction, angina, and sudden cardiac death. The incidence and severity of these adverse events are influenced by treatment type, cumulative dose, and administration schedule [11,12,13].

Pathophysiologically, PC and CVDs are linked by common mechanisms such as oxidative stress, immune dysregulation, and a pro-inflammatory state. PC is also characterized by an intrinsic hypercoagulable state driven by the release of inflammatory cytokines, activation of platelets, and upregulation of coagulation-promoting factors such as tissue factor and thrombin [14]. These changes significantly elevate the risk of thrombotic events. The development of diabetes following pancreatectomy, a common procedure in PC management, further increases the risk of ischemic heart disease [15].

Our systematic review and meta-analysis aimed to evaluate the prevalence of CVDs in patients with pancreatic ductal adenocarcinoma (PDAC) at diagnosis and their incidence during treatment according to disease stage and treatment type.

## 2. Materials and Methods

Our meta-analysis followed the recommendation of the Preferred Reporting Items for Systematic Reviews and Meta-Analyses (PRISMA) 2020 guideline [16] (Supplementary Table S1) and the Cochrane Handbook [17]. The study protocol was registered in PROSPERO (CRD42023482295), and we fully adhered to it. The research project was conducted under the Systems Education model coordinated by the Centre for Translational Medicine, Semmelweis University, and the Hungarian Pancreatic Study Group [18,19].

### 2.1. Eligibility Criteria

We included randomized controlled trials (RCTs) and cohort and case–control studies reporting the prevalence or incidence of CVDs in adult patients diagnosed with PC. Exclusion criteria were the following: non-human studies, pediatric population, case reports, case series, reviews, meta-analyses, conference abstracts, and pancreatic tumors other than PDAC.

### 2.2. Search Strategy

Our systematic search was conducted in three databases: PubMed, EMBASE, and Cochrane Central Register of Controlled Trials (CENTRAL) from inception to 13 November 2023. Our search key contained two domains, one related to PC and the other to CVDs (the detailed search key is available in Supplementary Table S2). In addition, we applied the Reference Citation Analysis (RCA) tool [20] to screen the reference lists of the identified, eligible manuscripts for additional articles. No language or time restrictions were applied.

### 2.3. Selection Process

After the systematic search, the yielded articles were imported into a reference management program (EndNote 20.1, Clarivate Analytics, Philadelphia, PA, USA). Duplicates were removed automatically and then manually. Screening and selection were performed first by title and abstract, and then by full-text content in four teams of two independent investigators working in pairs (E.G. and V.L, I.C. and O.E., J.L and J.N., Z.B. and J.N.). Cohen’s kappa coefficient (κ) was calculated after each selection step to evaluate inter-reviewer agreement. Disagreements were resolved by third party arbitration (J.N. or O.E., respectively).

### 2.4. Data Collection Process and Data Item

All data were manually collected into a standardized data collection sheet. The same investigators extracted relevant data from eligible studies. Disagreements were resolved by a third independent investigator (J.N. and O.E. respectively). The following data were extracted: first author, year of publication, digital object identifier, study design, study period, median or mean age of participants, sex distribution, tumor stage and grade, treatment type, type of CVD, and rate of CVD at baseline (prevalence) or during treatment (incidence) with the corresponding 95% confidence intervals (CIs).

### 2.5. Synthesis Methods

As considerable between-study heterogeneity was assumed, random-effects models were used. All statistical analyses were performed with R (R Core Team 2021, v4.4.1) using the meta package for basic meta-analysis calculations and plots, and dmetar package for additional influential analysis calculations and plots.

For both prevalences and incidences, proportions were used as effect size measure. The number of all patients and those with the event of interest was extracted from each study. Data were separated according to whether the study reported the prevalence of a given condition within their patient population, or events during follow-up, for prevalence and incidence meta-analyses, respectively. Incidences were planned to be subgrouped based on the length of follow-up, but cutoffs between subgroups were determined after data extraction. Where possible (at least three studies could be pooled), we conducted subgroup analyses based on treatment type and cancer stage. Random intercept logistic regression models were used to pool proportions as [21,22] with a maximum likelihood estimator for the heterogeneity variance measure (τ^2^) and using the Hartung-Knapp adjustment [23,24] for CIs. Between-study heterogeneity was described using Higgins & AMP and Thompson’s *I*^2^ statistics [25]. Potential outlier publications were explored using different influence measures and plots. For subgroup analysis, we used a mixed-effects model, assuming different τ^2^ values in the subgroups. Meta-analysis findings were summarized in forest plots, using the Clopper–Pearson method [26] for the CI of individual study proportions and including prediction intervals (i.e., the expected range of effects of future studies). Small study publication bias was assessed by visual inspection of Funnel-plots and Peters (modified Egger’s) test [27] for analyses with at least ten studies, assuming possible small study bias with *p* values below 0.1.

### 2.6. Risk of Bias Assessment

Two authors performed the risk of bias assessment in parallel using the Joanna Briggs Institute Prevalence Critical Appraisal Tool (JBI) [28]. Disagreements were resolved by third party arbitration. The tool contains nine items regarding the target population and study settings. Each item was rated as ‘yes’, ‘no’, ‘unclear’, or ‘not applicable’ according to information provided in each study, with a maximum score of nine points. (Supplementary Table S3)

## 3. Results

### 3.1. Search and Selection

**Figure 1:**
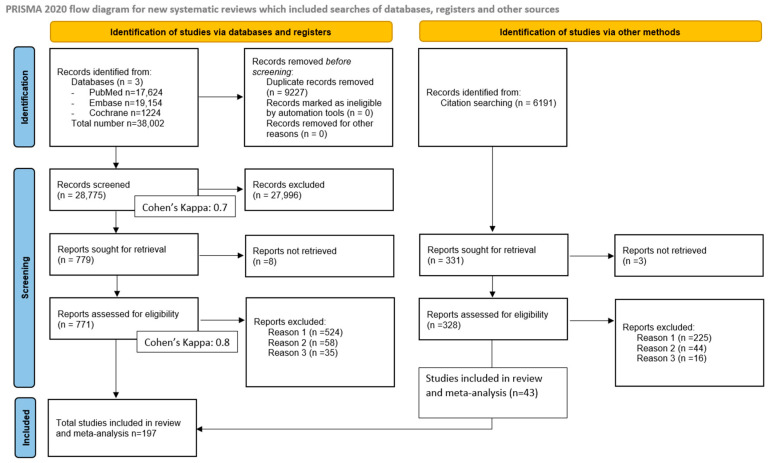
PRISMA 2020 flow chart—literature screening process.

Altogether, 38,002 studies were found in our systematic search. After duplicate removal, 28,775 were screened by title and abstract content. In total, 197 studies were included in the systematic review, of which 154 were identified in the original pool and 43 were found using Citation Chaser. Details of the selection are illustrated in the PRISMA 2020 flow chart (Figure 1).

### 3.2. Basic Characteristics of Included Studies

**Figure 2:**
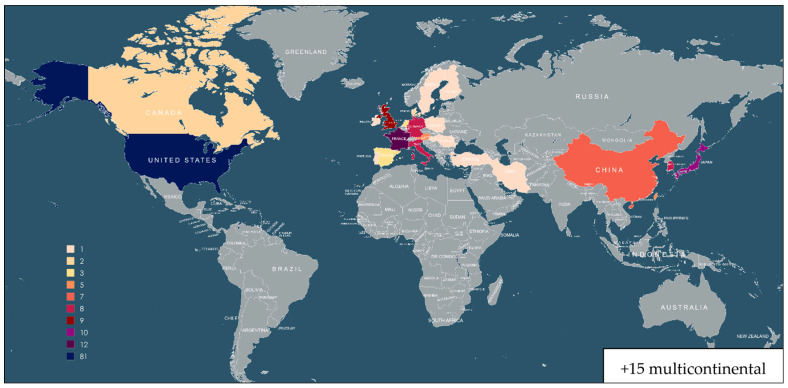
Geographical distribution of selected articles—the numbers next to the colored boxes indicate the number of articles from each country.

We included 110 cohort analyses and 87 RCTs. The geographical distribution of the included studies is summarized in Figure 2: 32 records were from Asia, 60 from Europe, 91 from the United States, and 15 were intercontinental. In total, more than 1,800,000 patients were included in the analysis. The main characteristics of the included studies are detailed in Supplemental Table S4. The definitions of the CVDs reported in each article are given in Supplemental Table S5. Figure 2 illustrates the geographical distribution of the selected articles.

### 3.3. The Rate of Cardiovascular Events in Pancreatic Adenocarcinoma

The prevalence of most investigated CVDs in patients with PDAC at diagnosis was similar to that in the general population. The most frequently reported conditions were hypertension, affecting approximately one in three patients (33%, CI: 27–40%), and ischemic heart disease (6%, CI: 3–12%). Heart failure, arrhythmia, and stroke each affected 2–3% of patients at diagnosis (Table 1, Supplemental Figure S1A–E).

The overall incidence of most investigated CVDs during treatment was below 10% for each evaluated event across all disease stages and regardless of treatment type. Follow-up time varied from 1 to 52 months. Hypertension was the most common, affecting 9% (CI: 5–15%) of patients, while hypotension, heart failure, stroke, or arrhythmia affected up to 5% of the cases. (Table 1) Subgroup analyses were conducted according to cancer stage and treatment type. Types of therapeutic agents are detailed in Supplemental Table S6. Unresectable disease stage and treatment type (gemcitabine-based, small-molecule inhibitors associated and monoclonal antibodies associated treatments) were not associated with a higher incidence of CVDs (Supplemental Figures S2–S7).

### 3.4. The Rate of Thromboembolic Events in Patients with PC

**Figure 3:**
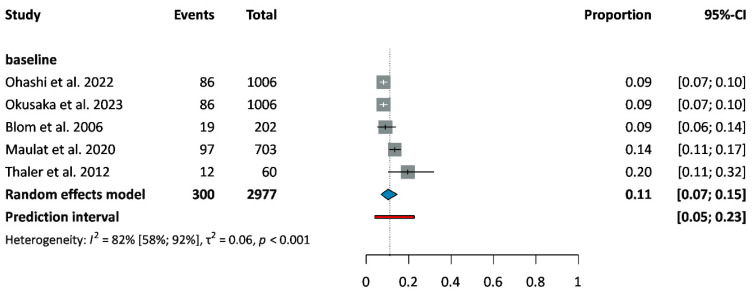
Prevalence of thrombotic events in patients with PC at baseline [29,30,31,32,33]. Abbreviations: CI: confidence interval.

**Figure 4:**
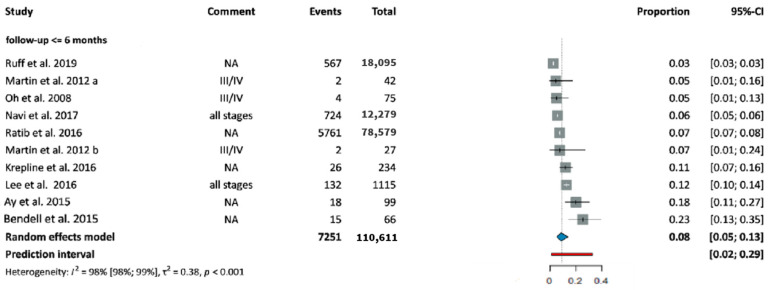
Incidence of thrombotic events in patients with PC during the first 6 months of treatment [34,35,36,37,38,39,40,41,42]. Abbreviations: CI: confidence interval.

Thromboembolic events were categorized as pulmonary embolism and other thrombotic events respectively (including deep vein thrombosis, splenic vein thrombosis, superficial vein thrombosis, portal vein thrombosis, splanchnic vein thrombosis, and arterial embolism). The prevalence of thrombotic events at baseline was 11% (CI: 7–15%) (Figure 3) [29,30,31,32,33]. Their incidence during treatment was relatively stable, affecting 8% (CI: 5–13%) of patients during the first six months of follow-up (Figure 4) [34,35,36,37,38,39,40,41,42].

Further subgroup analyses were performed according to treatment type and disease stage for the incidence of thrombotic events. (Table 2, Supplemental Figures S9–S12) They affected 8–10% of cases, regardless of treatment type. Follow-up period ranged from 1 to 60 months. The lowest incidence was found in those who received gemcitabine-based therapy and were evaluated at one or more years after diagnosis, affecting 6% of cases (CI: 3–10%). (Supplemental Figure S10A).

**Figure 5:**
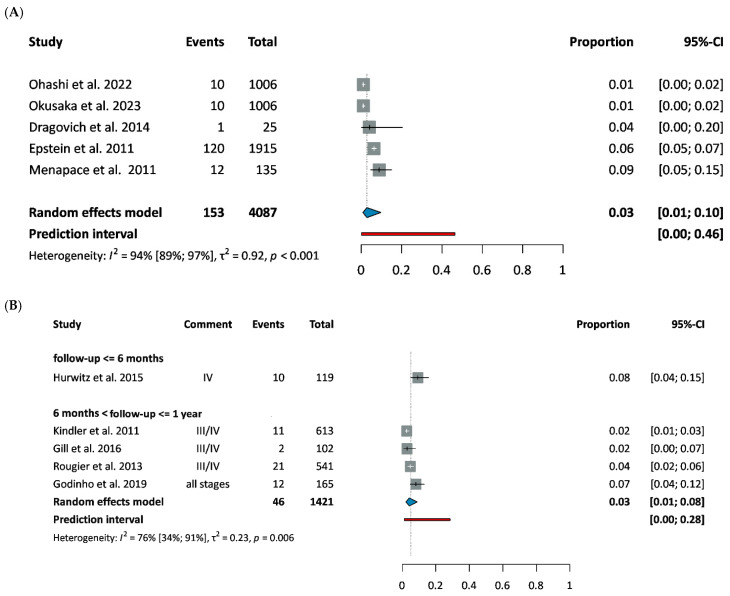
Prevalence (**A**) and incidence (**B**) of pulmonary embolism among PC patients [31,32,43,44,45,46,47,48,49,50]. Abbreviations: CI: confidence interval.

Pulmonary embolism affected 3% of PDAC patients at baseline [31,32,43,44,45] (Figure 5A) and developed in 3–4% of patients during follow-up, irrespective of treatment type and disease stage [46,47,48,49,50] (Figure 5B, Supplemental Figure S13). Follow-up period varied between 7 and 60 months, and the incidence did not vary in time (Supplemental Figure S13).

We additionally conducted incidence analyses stratified by study design, specifically comparing observational studies and randomized controlled trials, regarding hypertension, pulmonary embolism and thrombotic events. We found no substantial difference in the incidence rates between these categories; the detailed results are presented in Supplemental Figures S14–S16.

### 3.5. Risk of Bias Assessment

The overall risk of bias in the selected studies was low to moderate. (Supplemental Table S3) She sources of bias across the included articles comprised unclear definition of the diagnostic methodology or criteria, imprecise description of the measurement of the condition, lack of details on statistical analysis, or inadequate response rate. Significant heterogeneity (greater than 80%) was observed across all our analyses. Peters’ test suggested the presence of small-study effects (*p* < 0.001). The funnel plot assessing publication bias is presented in Supplemental Figure S17.

## 4. Discussion

The aim of our study was to evaluate the prevalence and incidence of CVD among patients with PC. The prevalence of the investigated NT-CVDs in PDAC was similar to that in the general population. The most frequent condition was hypertension, which affected one in three patients at baseline. Ischemic heart disease affects 6% of patients, while the other investigated diseases, heart failure, arrhythmia, and stroke, occurred in 2–3% of cases at diagnosis, respectively. The incidence of the investigated CVDs was under 5% during the treatment period. Thrombotic events other than pulmonary embolism affected 11% of cases at baseline, and a similar rate of cases during the follow-up. Pulmonary embolism was present in 3% of patients at diagnosis and developed in a further 3–4% during treatment. The incidence of each CVD did not vary during follow-up, regardless of treatment type and disease stage.

The similar prevalence of NT-CVDs in PDAC patients and the general population may be explained by shared risk factors contributing to both conditions [51]. PC and CVDs can be linked to several common genetic and epigenetic changes. Various microRNAs have been described as contributing factors to the manifestation of CVDs, with some also showing alterations in PDAC [10,52,53]. The prevalence of CVDs, such as hypertension and arrhythmia, in patients with PDAC is comparable to that seen in other common cancers, including breast and lung cancer [54,55,56,57]. An exception is colorectal cancer, where hypertension appears slightly more prevalent, potentially due to microbiome-related mechanisms [58,59,60].

The development of cancer-associated coagulopathy is a complex and multifactorial process. A key aspect is the ability of tumor cells to activate the coagulation system of the host. As a result, cancerous tissues begin to produce procoagulant proteins that promote the development of clinically evident coagulopathy. Additionally, alterations in the stromal cells of the tumor during cancer progression may influence the hemostatic system [61,62,63]. Comorbidities, tumor-specific characteristics, as well as cancer therapies may also impact the risk of thrombotic events [64]. However, our findings suggest that the increased incidence of thrombotic events is more likely attributed to the presence of the malignancy itself rather than the consequences of the treatment, as the frequency of such events did not change over time, regardless of treatment type or follow-up duration. International guidelines on thrombosis and cancer recommend the use of thromboprophylaxis with low-molecular-weight heparin for different patient groups, including PC patients undergoing major surgery, hospitalized patients with acute medical conditions and limited mobility, as well as ambulatory PC patients with locally advanced or metastatic disease receiving chemotherapy [65,66]. Our results suggest that we should maintain heightened awareness of thrombosis in patients with PDAC, starting from diagnosis regardless of tumor burden or performance status.

It is also important to emphasise that, in addition to therapy and tumor-induced hypercoagulability, other external factors substantially contribute to the development of thrombotic events, such as immobility and the use of invasive devices. Immobility contributes to venous stasis, which, together with endothelial injury and hypercoagulability, underlies the increased incidence of venous thromboembolism in this population [67]. Central venous access devices further elevate the risk: prospective cohort studies report that venous thromboembolic events occur in approximately 5.9% of cancer patients within three months of port implantation and up to 15% at one year. Observational data indicate that catheter-related thrombosis affects around 3.6% of patients, with certain catheter types, such as PICCs, associated with higher rates. Although many catheter-associated thrombi are asymptomatic, they contribute substantially to morbidity. These findings underscore the necessity of vigilant monitoring and early prophylactic strategies in oncology patients with restricted mobility or indwelling venous catheters [68,69,70].

Gemcitabine, a widely used agent in PDAC treatment, has been associated with myocardial ischemia, pericardial effusion, supraventricular arrhythmias, and heart failure. These events are rare (≤1%) and usually occur within 1–2 months after initiation of treatment, except for heart failure, which tends to appear later and is often irreversible [71]. Similarly, fluoropyrimidines such as 5-FU and capecitabine are key chemotherapeutics with cardiovascular side effects reported in 1.2–7.6% of cases [12,72]. These include arrhythmias, myocardial infarction, and, rarely, sudden death [12,72]. Additionally, newer chemotherapies and targeted agents can also cause cardiotoxicity—ranging from arrhythmias to atherosclerosis—due to effects on healthy tissues mediated by oxidative stress, inflammation, immunothrombosis, and growth factor signaling [13]. However, we found no major changes in the incidences of CVDs among PDAC patients based on treatment type.

### 4.1. Strengths and Limitations

In terms of strengths, this is the first meta-analysis to investigate the proportion of CVDs in PC. We were able to include numerous studies comprising a high number of patients, and we performed clinically relevant subgroup analyses based on disease stage and treatment type. Moreover, we followed a robust and transparent methodology.

In terms of limitations, most of our analyses were characterized by a high degree of heterogeneity due to the variability in definitions and diagnostic criteria for CVDs and population characteristics across the included articles. We would like to highlight that in cases where the definitions and diagnostic criteria of the examined conditions may vary, such as hypertension, stroke, and heart failure, our findings should be interpreted as indicative trends rather than as precise prevalence estimates.

An additional limitation is that many studies provided insufficient information on the time of prevalence measurement and the length of follow-up periods. For several subgroups, the majority of the articles fell into the category of unknown follow-up duration.

### 4.2. Implications for Practice and Research

Based on these considerations, we recommend that future researchers and clinicians adopt standardized definitions in their research protocols. In addition, methodological improvements are necessary to ensure accurate documentation of follow-up duration and clear reporting of both the timing of adverse event onset and the specific time points of clinical evaluation, thereby helping to eliminate data gaps currently categorized as “unknown”.

Our findings highlight the importance of rigorous and extensive examination of patients at baseline, while maintaining a high index of suspicion for thromboembolic events, as these complications are already present in approximately one out of ten patients at the time of diagnosis and may not always be accompanied by clinical symptoms. Further studies are needed that focus on the intricacies of pathophysiological and molecular mechanisms between PC and CVDs.

## 5. Conclusions

Thromboembolic events are common in PDAC and occur both at diagnosis and during follow-up. Their incidence remains stable across treatment modalities and disease stages, suggesting an association with tumor-related hypercoagulability; however, causality cannot be established. The prevalence of NT-CVDs in PDAC is comparable to that in the general population and shows minimal variation across cancer stages or treatment modalities.

## Figures and Tables

**Table 1:** Prevalence at baseline and incidence during treatment of various CVDs among PDAC patients.

Cardiovascular Disease/Event	Prevalence at Baseline				Incidence During Treatment			
	Number of Studies	Total Number of Patients	Rate	95% CI	Number of Studies	Total Number of Patients	Rate	95% CI
Hypertension	39	76,328	33%	27–40%	34	51,885	9%	5–15%
Ischaemic heart disease	16	267,431	6%	3–12%	11	21,705	4%	2–9%
Stroke	12	267,378	3%	1–7%	17	76,067	1%	1–9%
Arrhythmia	9	256,829	3%	1–8%	17	22,472	3%	2–5%
Cardiac failure	11	325,356	2%	1–8%	4	13,481	2%	0–11%
Hypotension	NA				19	2179	5%	3–10%

Abbreviations: CI: confidence interval; NA: not available.

**Table 2:** Incidence of thrombotic events according to disease stage and treatment type.

Population	Number of Studies	Total Number of Patients	Rate (95% CI)
Overall	99	1,486,655	9% (7–10%)
Disease stage III–IV *^$^	54	7756	10% (8–12%)
Gemcitabine-based treatment **	41	6334	8% (6–11%)
Monoclonal antibodies associated treatment **	12	1660	9% (6–14%)
Small-molecule inhibitors associated treatment **	9	1213	8% (4–18%)

Abbreviations: CI: confidence interval. * Patients with TNM III-IV stage tumor; ^$^ various treatment types ** various disease stages.

## Data Availability

The datasets used in this study can be found in the full-text articles that were included in the systematic review and meta-analysis.

## Supplementary Materials

The following supporting information can be downloaded at: https://www.mdpi.com/article/10.3390/cancers18071108/s1, Table S1. Prisma checklist. Table S2. Searchkey. Table S3. Risk of bias assessment -using JBI tool. Table S4. Baseline characteristics of the included studies. Table S5. Provided methods underlying the definitions of cardiovascular diseases. Table S6. Therapeutic agents in treatments associated with A: small molecule inhibitors B: monoclonal antibodies. Figure S1. Prevalence of A: hypertension B: ischemic heart disease C: heart failure D: arrhythmia E: stroke among pancreatic cancer patients. Figure S2. Incidence of hypertension among patients A: with TNM III-IV B: who received Gemcitabine-based therapy C: treatment in association with small molecule inhibitors D: treatment in association with monoclonal antibodies. Figure S3. Incidence of ischemic heart disease among patients A: with TNM III-IV B: who received Gemcitabine-based therapy C: treatment in association with monoclonal antibodies. Figure S4. Incidence of stroke among patients A: with TNM III-IV B: who received Gemcitabine-based therapy C: treatment in association with small molecule inhibitors D: in association with monoclonal antibodies. Figure S5. Incidence of arrhythmia among patients A: with TNM III-IV B: who received Gemcitabine-based therapy. Figure S6. Incidence of cardiac failure among patients who received Gemcitabine-based therapy. Figure S7. Incidence of hypotension among patients A: with TNM III-IV B: who received Gemcitabine-based therapy. Figure S8. Incidence of thrombotic events with different follow-up times. Figure S9. Incidence of thrombotic events among patients with TNM III-IV. Figure S10. Incidence of thrombotic events among patients A: who received Gemcitabine-based therapy B: with TNM III-IV who received Gemcitabine-based therapy. Figure S11. Incidence of thrombotic events among patients A: who received treatment in association with monoclonal antibodies B: with TNM III-IV who received treatment in association with monoclonal antibodies. Figure S12. Incidence of thrombotic events among patients who received treatment in association with small molecule inhibitors. Figure S13. Incidence of pulmonary embolism among patients A: with TNM III-IV B: who received Gemcitabine-based therapy C: with TNM III-IV who received treatment in association with small molecule inhibitor. Figure S14. Incidence of hypertension A: including observational studies B: including randomised controlled trials. CI: confidence interval. Figure S15. Incidence of pulmonary embolism A: including observational studies B: including randomised controlled trials. CI: confidence interval. Figure S16. Incidence of thrombotic events A: including observational studies B: including randomised controlled trials. CI: confidence interval. Figure S17. Funnel plot for the analysis of the incidence of thrombotic events.

## Abbreviations

The following abbreviations are used in this manuscript:

CENTRAL	Cochrane Central Register of Controlled Trials
CI	confidence interval
CVD	cardiovascular disease
JBI	Joanna Briggs Institute Prevalence Critical Appraisal Tool
NT-CVD	non-thrombotic cardiovascular disease
PC	pancreatic cancer
PDAC	pancreatic ductal adenocarcinoma
PRISMA	Preferred Reporting Items for Systematic Reviews and Meta-Analyses
RCT	randomized controlled trial
