# Supplementary material for: Cardiovascular Risk in Pancreatic Cancer: A Meta-Analysis of 197 Studies
Source: Cancers (Basel). 2026 Mar 29;18(7):1108. doi: 10.3390/cancers18071108 (PMC13072353; doi:10.3390/cancers18071108)
Supplement: Supplementary file 1 [file cancers-18-01108-s001.zip › cancers-4163263-supplementary.pdf]

Supplemental Materials

Cardiovascular Risk in Pancreatic Cancer: A Meta-Analysis of 197 Studies

Authors

Jázmin Németh [1,2], Jimin Lee [1], Orsolya Eperjesi [1,3,4], Endre Botond Gagyi [1,5,6], Zoltán Bánfalvi [1,4], Veronika Lillik [1,7], Ioana Creanga-Murariu [1,8], Réka Tóth [1], Eszter Ágnes Szalai [1,9], Mahmoud Obeidat [1], Szilárd Váncsa [1,4], Stefania Bunduc [1,2,10,11,*,†] and Péter Hegyi [1,2,4,12,†]

Affiliations:

1 Centre for Translational Medicine, Semmelweis University, 1085 Budapest, Hungary; nemethjazmin222@gmail.com (J.N.); jmnlee10@gmail.com (J.L.); eperjesiorsi1348@gmail.com (O.E.); endre.gg@gmail.com (E.B.G.); banfalvi.zoltan11@gmail.com (Z.B.); ioana.creanga@d.umfiasi.ro (I.C.-M.); rekatoth83@gmail.com (R.T.); obeidat.mahmoud96@gmail.com (M.O.); vancsa.szilard@gmail.com (S.V.); hegyi2009@gmail.com (P.H.)

2 Institute for Translational Medicine, Medical School, University of Pécs, 7622 Pécs, Hungary

3 Department of Internal Medicine, Toldy Ferenc Hospital, 2700 Cegléd, Hungary

4 Institute of Pancreatic Diseases, Semmelweis University, 1085 Budapest, Hungary

5 Department of Medical Imaging, Bajcsy-Zsilinszky Hospital and Clinic, 1106 Budapest, Hungary

6 Selye János Doctoral College for Advanced Studies, Semmelweis University, 1085 Budapest, Hungary

7 Fejér County Szent György University Teaching Hospital, 8000 Székesfehérvár, Hungary

8 Advanced Research and Development Center for Experimental Medicine (CEMEX), "Grigore T. Popa" University of Medicine and Pharmacy, 700454 Iasi, Romania

9 Department of Restorative Dentistry and Endodontics, Semmelweis University, 1085 Budapest, Hungary

10 Carol Davila University of Medicine and Pharmacy, 020021 Bucharest, Romania

11 Digestive Disease and Liver Transplant Centre, Fundeni Clinical Institute, 022328 Bucharest, Romania

12 Translational Pancreatology Research Group, Interdisciplinary Centre of Excellence for Research Development and Innovation, University of Szeged, 6720 Szeged, Hungary

* Correspondence: stfnbndc@gmail.com; Tel.: +40-747-90-11-98

† These authors contributed equally to this work.

SUPPLEMENTAL MATERIALS

Table legends

Table S1. Prisma checklist…………………………………………………………………………………….…...…4

Table S2. Searchkey…………………………………………………………………………………………….….…7

Table S3. Risk of bias assessment -using JBI tool……………………………………..……………………………………………10

Table S4. Baseline caracteristis of included studies…………………………………………………………………………...…16

Table S5. Provided methods underlying the definitions of cardiovascular diseases.…………………………………………………..31

Table S6. Therapeutic agents in treatments associated with A: small molecule inhibitors B: monoclonal antibodies………………………………...38

Figure legends

Figure S1. Prevalence of A: hypertension B: ischemic heart disease C: heart failure D: arrhythmia E: stroke among pancreatic cancer patients……………...39

Figure S2. Incidence of hypertension among patients A: with TNM III-IV B: who received Gemcitabine-based therapy C: treatment in association with small molecule inhibitors D: treatment in association with monoclonal antibodies……………………………………………………………………………41

Figure S3. Incidence of ischemic heart disease among patients A: with TNM III-IV B: who received Gemcitabine-based therapy C: treatment in association with monoclonal antibodies…………………………………………………………………………………………………43

Figure S4. Incidence of stroke among patients A: with TNM III-IV B: who received Gemcitabine-based therapy C: treatment in association with small molecule inhibitors D: in association with monoclonal antibodies…………………………………………………………………………...44

Figure S5. Incidence of arrhythmia among patients A: with TNM III-IV B: who received Gemcitabine-based therapy………………………………………..46

Figure S6. Incidence of cardiac failure among patients who received Gemcitabine-based therapy……………………………………………………………...47

Figure S7. Incidence of hypotension among patients A: with TNM III-IV B: who received Gemcitabine-based therapy……………………………………47

Figure S8. Incidence of thrombotic events with different follow-up times……………………………………………………………………………….48

Figure S9. Incidence of thrombotic events among patients with TNM III-IV……………………………………………………………………….....48

Figure S10. Incidence of thrombotic events among patients A: who received Gemcitabine-based therapy B: with TNM III-IV who received Gemcitabine-based therapy………....49

Figure S11. Incidence of thrombotic events among patients A: who received treatment in association with monoclonal antibodies B: with TNM III-IV who received treatment in association with monoclonal antibodies…………………………………………………………………………………………..50

Figure S12. Incidence of thrombotic events among patients who received treatment in association with small molecule inhibitors……………………………51

Figure S13. Incidence of pulmonary embolism among patients A: with TNM III-IV B: who received Gemcitabine-based therapy C: with TNM III-IV who received treatment in association with small molecule inhibitors……………………………………………………………………………………..52

Figure S14. Incidence of hypertension A: including observational studies B: including randomised controlled trials. CI: confidence interval……………………………..53

Figure S15. Incidence of pulmonary embolism A: including observational studies B: including randomised controlled trials. CI: confidence interval……………………….55

Figure S16. Incidence of thrombotic events A: including observational studies B: including randomised controlled trials. CI: confidence interval…………………………..56

Figure S17. Funnel plot for the analysis of the incidence of thrombotic events………………………………………………………………………………….57

Table S1. Prisma checklist.

Section and Topic	Item #	Checklist item	Location where item is reported
**TITLE**			
Title	1	Identify the report as a systematic review.	1
**ABSTRACT**			
Abstract	2	See the PRISMA 2020 for Abstracts checklist.	3
**INTRODUCTION**			
Rationale	3	Describe the rationale for the review in the context of existing knowledge.	5
Objectives	4	Provide an explicit statement of the objective(s) or question(s) the review addresses.	5
**METHODS**			
Eligibility criteria	5	Specify the inclusion and exclusion criteria for the review and how studies were grouped for the syntheses.	6
Information sources	6	Specify all databases, registers, websites, organisations, reference lists and other sources searched or consulted to identify studies. Specify the date when each source was last searched or consulted.	6
Search strategy	7	Present the full search strategies for all databases, registers and websites, including any filters and limits used.	6
Selection process	8	Specify the methods used to decide whether a study met the inclusion criteria of the review, including how many reviewers screened each record and each report retrieved, whether they worked independently, and if applicable, details of automation tools used in the process.	6
Data collection process	9	Specify the methods used to collect data from reports, including how many reviewers collected data from each report, whether they worked independently, any processes for obtaining or confirming data from study investigators, and if applicable, details of automation tools used in the process.	6-7
Data items	10a	List and define all outcomes for which data were sought. Specify whether all results that were compatible with each outcome domain in each study were sought (e.g. for all measures, time points, analyses), and if not, the methods used to decide which results to collect.	7
	10b	List and define all other variables for which data were sought (e.g. participant and intervention characteristics, funding sources). Describe any assumptions made about any missing or unclear information.	7
Study risk of bias assessment	11	Specify the methods used to assess risk of bias in the included studies, including details of the tool(s) used, how many reviewers assessed each study and whether they worked independently, and if applicable, details of automation tools used in the process.	7,13
Effect measures	12	Specify for each outcome the effect measure(s) (e.g. risk ratio, mean difference) used in the synthesis or presentation of results.	7
Synthesis methods	13a	Describe the processes used to decide which studies were eligible for each synthesis (e.g. tabulating the study intervention characteristics and comparing against the planned groups for each synthesis (item #5)).	7-8
	13b	Describe any methods required to prepare the data for presentation or synthesis, such as handling of missing summary statistics, or data conversions.	7-8

	13c	Describe any methods used to tabulate or visually display results of individual studies and syntheses.	7-8
	13d	Describe any methods used to synthesize results and provide a rationale for the choice(s). If meta-analysis was performed, describe the model(s), method(s) to identify the presence and extent of statistical heterogeneity, and software package(s) used.	7-8
	13e	Describe any methods used to explore possible causes of heterogeneity among study results (e.g. subgroup analysis, meta-regression).	7-8
	13f	Describe any sensitivity analyses conducted to assess robustness of the synthesized results.	NA
Reporting bias assessment	14	Describe any methods used to assess risk of bias due to missing results in a synthesis (arising from reporting biases).	8
Certainty assessment	15	Describe any methods used to assess certainty (or confidence) in the body of evidence for an outcome.	NA
**RESULTS**			
Study selection	16a	Describe the results of the search and selection process, from the number of records identified in the search to the number of studies included in the review, ideally using a flow diagram.	9
	16b	Cite studies that might appear to meet the inclusion criteria, but which were excluded, and explain why they were excluded.	9
Study characteristics	17	Cite each included study and present its characteristics.	Supplementary Table 4.
Risk of bias in studies	18	Present assessments of risk of bias for each included study.	Supplementary Table 3.
Results of individual studies	19	For all outcomes, present, for each study: (a) summary statistics for each group (where appropriate) and (b) an effect estimate and its precision (e.g. confidence/credible interval), ideally using structured tables or plots.	11-13
Results of syntheses	20a	For each synthesis, briefly summarise the characteristics and risk of bias among contributing studies.	Supplementary Table 3., Supplementary Table 4.
	20b	Present results of all statistical syntheses conducted. If meta-analysis was done, present for each the summary estimate and its precision (e.g. confidence/credible interval) and measures of statistical heterogeneity. If comparing groups, describe the direction of the effect.	11-13. Supplementary Figure 1-13.
	20c	Present results of all investigations of possible causes of heterogeneity among study results.	NA
	20d	Present results of all sensitivity analyses conducted to assess the robustness of the synthesized results.	NA
Reporting biases	21	Present assessments of risk of bias due to missing results (arising from reporting biases) for each synthesis assessed.	13
Certainty of evidence	22	Present assessments of certainty (or confidence) in the body of evidence for each outcome assessed.	NA
**DISCUSSION**			
Discussion	23a	Provide a general interpretation of the results in the context of other evidence.	14
	23b	Discuss any limitations of the evidence included in the review.	16

	23c	Discuss any limitations of the review processes used.	16
	23d	Discuss implications of the results for practice, policy, and future research.	16
**OTHER INFORMATION**			
Registration and protocol	24a	Provide registration information for the review, including register name and registration number, or state that the review was not registered.	6
	24b	Indicate where the review protocol can be accessed, or state that a protocol was not prepared.	6
	24c	Describe and explain any amendments to information provided at registration or in the protocol.	NA
Support	25	Describe sources of financial or non-financial support for the review, and the role of the funders or sponsors in the review.	2
Competing interests	26	Declare any competing interests of review authors.	2
Availability of data, code and other materials	27	Report which of the following are publicly available and where they can be found: template data collection forms; data extracted from included studies; data used for all analyses; analytic code; any other materials used in the review.	17

From: Page MJ, McKenzie JE, Bossuyt PM, Boutron I, Hoffmann TC, Mulrow CD, et al. The PRISMA 2020 statement: an updated guideline for reporting systematic reviews. BMJ 2021;372:n71. doi: 10.1136/bmj.n71.
This work is licensed under CC BY 4.0. To view a copy of this license, visit https://creativecommons.org/licenses/by/4.0/

Table S2. Search-key.

Database	Domain 1.		Domain 2.
Pubmed	((((pancrea*) AND (tumo* OR malignan* OR "carcinoma" OR "cancer" OR "adenocarcinoma")) OR "PDAC"))	AND	(((("heart" OR aort* OR cardi* OR "cordis" OR "cerebrovascular" OR "vascular" OR "vein" OR veno* OR atri* OR "myocardial" OR "coronary" OR ventric* OR ischaemi* OR "diastolic" OR "systolic") AND ("cachexia" OR "insufficiency" OR "accident" OR "necrosis" OR "fibrosis" OR thrombo* OR embo* OR "occlusion" OR diseas* OR "stenosis" OR "rupture" OR "perforation" OR "deficiency" OR disorde* OR "dysfunction" OR "emergency" OR "inflammation" OR "rate" OR "degeneration" OR "defect" OR "prolapse" OR "murmur" OR "regurgitation" OR "hypertrophy" OR "contracture" OR "dilatation" OR "enlargement" OR "hyperplasia" OR "dissociation" OR "cardiopathy" OR "event" OR "remodeling" OR "infarction" OR "atherosclerosis" OR "syndrome" OR "overdriving" OR "overloading" OR "disturbance" OR "toxicity" OR "arrhythmia" OR "fibrillation" OR "flutter" OR "palpitation" OR "preexcitation" OR "proarrhythmia" OR "problem" OR "calcinosis" OR "calcification" OR "death" OR "failure" OR decompensatio* OR "incompetence" OR "arrest" OR "injury" OR "damage" OR "lesion" OR "trauma" OR "stress")) OR ("parasystole" OR "tachycardia" OR "bradycardia" OR "cardiotoxicity" OR "angina" OR "MINOCA" OR "cardiomyopathy" OR "STEMI" OR "NSTEMI" OR "hypertension" OR ("blood" AND "pressure") OR "stroke" OR "endoleak" OR aneur* OR "vasculopathy" OR "cardiomyofibrosis" OR pericard*)))
Embase	(((((pancrea*) AND (tumo* OR malignan* OR carcinoma OR cancer OR adenocarcinoma)) OR PDAC))	AND	((((heart OR aort* OR cardi* OR cordis OR cerebrovascular OR vascular OR vein OR veno* OR atri* OR myocardial OR coronary OR ventric* OR ischaemi* OR diastolic OR systolic) AND (cachexia OR insufficiency OR accident OR necrosis OR fibrosis OR thrombo* OR embo* OR occlusion OR diseas* OR stenosis OR rupture OR perforation OR deficiency OR disorde* OR dysfunction OR emergency OR inflammation OR rate OR degeneration OR defect OR prolapse OR murmur OR regurgitation OR hypertrophy OR contracture OR dilatation OR enlargement OR hyperplasia OR dissociation OR cardiopathy OR event OR remodeling OR infarction OR atherosclerosis OR syndrome OR overdriving OR overloading OR disturbance OR toxicity OR arrhythmia OR fibrillation OR flutter OR palpitation OR preexcitation OR proarrhythmia OR problem OR calcinosis OR calcification OR death OR failure OR decompensatio* OR incompetence OR arrest OR injury OR damage OR lesion OR trauma OR stress)) OR (parasystole OR tachycardia OR bradycardia OR cardiotoxicity OR angina OR MINOCA OR cardiomyopathy OR STEMI OR NSTEMI OR hypertension OR (blood AND pressure) OR stroke OR endoleak OR aneur* OR vasculopathy OR cardiomyofibrosis OR pericard*)))):ab,kw,ti
Cochrane Library	((((pancrea*) AND (tumo* OR malignan* OR carcinoma OR cancer OR adenocarcinoma)) OR PDAC))	AND	((((heart OR aort* OR cardi* OR cordis OR cerebrovascular OR vascular OR vein OR veno* OR atri* OR myocardial OR coronary OR ventric* OR ischaemi* OR diastolic OR systolic) AND (cachexia OR insufficiency OR accident OR necrosis OR fibrosis OR thrombo* OR embo* OR occlusion OR diseas* OR stenosis OR rupture OR perforation OR deficiency OR disorde* OR dysfunction OR emergency OR inflammation OR rate OR degeneration OR defect OR prolapse OR murmur OR regurgitation OR hypertrophy OR contracture OR dilatation OR enlargement OR hyperplasia OR dissociation OR cardiopathy OR event OR remodeling OR infarction OR atherosclerosis OR syndrome OR overdriving OR overloading OR disturbance OR toxicity OR arrhythmia OR fibrillation OR flutter OR palpitation OR preexcitation OR proarrhythmia OR problem OR calcinosis OR calcification OR death OR failure OR decompensatio* OR incompetence OR arrest OR injury OR damage OR lesion OR trauma OR stress)) OR (parasystole OR tachycardia OR bradycardia OR cardiotoxicity OR angina OR MINOCA OR cardiomyopathy OR STEMI OR NSTEMI OR hypertension OR (blood AND pressure) stroke OR endoleak OR aneur* OR vasculopathy OR cardiomyofibrosis OR pericard*)))

Table S3. Risk of bias assessment -using JBI tool.

First Author	Year of publication	1. Was the sample frame appropriate to adress the target population ?	2. Were study participants sampled in an appropriate way?	3. Was the sample size adequate ?	4. Were the study subjects and the settings described in detail?	5. Was the data analysis conducted with sufficient coverage of the identified sample?	6. Were valid methods used for the identification of the condition?	7. Was the condition measured in a standard, reliable way for all participants?	8. Was there appropriate statistical analysis?	9. Was the response rate adequate, and if not, was the low response rate managed appropriately?
Navi et al.	2017	Yes	Yes	yes	Yes	yes	Yes	Yes	Yes	Unclear
Baluka et al.	2016	Yes	Yes	yes	Yes	yes	Unclear	Unclear	Yes	Yes
Huang et al.	2022	Yes	Yes	yes	Yes	yes	Unclear	Unclear	Yes	Unclear
Lee et al.	2016	Yes	Yes	yes	Yes	yes	Unclear	Unclear	Yes	Yes
Ryan et al.	2018	Yes	Yes	yes	Yes	yes	Unclear	Unclear	Yes	Yes
Shen et al.	2022	Yes	Yes	yes	Yes	yes	Unclear	Unclear	Yes	Unclear
Tonnesen et al.	2023	Yes	Yes	yes	Yes	yes	Yes	Yes	Yes	Unclear
Sogaard et al.	2016	Yes	Yes	yes	Yes	yes	Yes	Yes	Yes	Yes
Anker et al.	2016	Yes	Yes	yes	Yes	yes	Yes	Yes	Yes	Unclear
Ratib et al.	2016	Yes	Yes	yes	No	yes	Yes	Yes	Yes	Unclear
Goncalves et al.	2012	Yes	Yes	yes	Yes	yes	Unclear	Unclear	Yes	Yes
Le Bozec et al.	2023	Yes	Yes	yes	Yes	yes	Unclear	Unclear	Yes	Unclear
Navi et al.	2015	Yes	Yes	yes	Yes	yes	Yes	Yes	Yes	Unclear
Navi et al.	2018	Yes	Yes	yes	No	yes	Yes	Yes	Yes	Unclear
Faille et al.	2018	Yes	Yes	yes	Yes	yes	Yes	Yes	Yes	Unclear
Fogelman et al.	2011	Yes	Yes	yes	Yes	yes	Unclear	Unclear	Yes	Yes
Li et al.	2015	Yes	Yes	yes	Yes	yes	Yes	Yes	Yes	Unclear
Yip et al.	2005	yes	yes	yes	no	yes	unclear	unclear	unclear	yes
Couturaud et al.	2023	Yes	Yes	yes	Yes	yes	Yes	Yes	Yes	Unclear

Stitzel et al.	2023	Yes	Yes	yes	Yes	yes	Yes	Yes	Yes	Yes
Kindler et al.	2011	Yes	Yes	yes	Yes	yes	Yes	Yes	Yes	Yes
Lee et al.	2016	Yes	Yes	yes	Yes	yes	Yes	Yes	Yes	Yes
Javle et al.	2009	Yes	Yes	yes	Yes	yes	Unclear	Unclear	Yes	Unclear
Jiang et al.	2017	Yes	Yes	yes	Yes	yes	Unclear	Unclear	Yes	Unclear
Cai et al.	2019	Yes	Yes	yes	Yes	yes	Unclear	Unclear	Yes	Unclear
Tingle et al.	2020	Yes	Yes	yes	Yes	yes	Unclear	Unclear	Yes	Unclear
Corrie et al.	2020	Yes	Yes	yes	Yes	yes	Unclear	Unclear	Yes	Yes
Rosemurgy et al.	2008	Yes	Yes	Yes	Yes	Yes	Unclear	Unclear	Yes	Unclear
Menapace et al.	2011	yes	yes	yes	yes	yes	yes	yes	yes	yes
Huang et al.	2023	yes	yes	yes	yes	yes	yes	yes	Yes	yes
Ishigaki et al.	2017	yes	yes	yes	yes	yes	yes	yes	Yes	yes
Pant et al.	2014	yes	yes	yes	yes	yes	yes	Unclear	yes	yes
Zhai et al.	2019	yes	yes	no	yes	yes	unclear	unclear	unclear	unclear
Rivera et al.	2019	yes	yes	yes	yes	yes	yes	yes	yes	yes
Sgori et al.	2015	yes	yes	yes	yes	yes	Unclear	Unclear	yes	yes
Martin et al.	2012	yes	yes	yes	yes	yes	Unclear	Unclear	unclear	yes
Wun et al.	2009	yes	yes	yes	no	yes	Unclear	Unclear	unclear	Unclear
Chen et al.	2018	yes	yes	yes	yes	yes	yes	yes	yes	yes
Suzuki et al.	2021	yes	yes	yes	yes	yes	yes	yes	Yes	yes
Laderman et al.	2023	yes	yes	yes	yes	yes	yes	yes	yes	yes
Jeong et al.	2023	yes	yes	yes	yes	yes	yes	yes	yes	yes
Ruff et al.	2019	yes	yes	yes	yes	yes	yes	yes	yes	yes
Oh et al.	2008	yes	yes	yes	yes	yes	yes	yes	yes	yes
Mandala et al.	2007	yes	yes	yes	yes	yes	yes	yes	yes	yes
Krepline et al.	2016	yes	yes	yes	yes	yes	yes	yes	yes	yes
Lyman et al.	2013	yes	yes	yes	yes	yes	yes	yes	yes	yes
Kim et al.	2016	yes	yes	yes	yes	yes	Unclear	Unclear	yes	yes
Rich et al.	2012	yes	yes	yes	yes	yes	yes	yes	yes	yes
Katz et al.	2016	yes	yes	yes	yes	yes	yes	yes	yes	yes
Zhao et al.	2022	yes	yes	yes	no	yes	yes	yes	yes	yes
Alberts et al.	2005	yes	yes	yes	yes	yes	yes	yes	yes	yes
O'Reilly et al.	2020	yes	yes	yes	yes	yes	yes	yes	yes	yes

Infante et al.	2014	yes	yes	yes	yes	yes	yes	yes	yes	yes
Rougier et al.	2013	yes	yes	yes	yes	yes	yes	yes	yes	yes
Friess et al.	2006	yes	yes	yes	yes	yes	yes	yes	yes	yes
El-Khoueiry et al.	2012	yes	yes	yes	yes	yes	yes	yes	yes	yes
Noel et al.	2022	yes	yes	yes	yes	yes	yes	yes	yes	yes
Burtness et al.	2016	yes	yes	yes	yes	yes	yes	yes	yes	yes
He et al.	2023	Yes	Yes	Yes	Yes	Yes	Yes	Yes	Yes	Yes
Nakai et al.	2010	Yes	Yes	Yes	Yes	Yes	unclear	unclear	Yes	Yes
Kamarajah et al.	2021	Yes	Yes	Yes	Yes	Yes	unclear	unclear	Yes	Yes
Rocha et al.	2016	Yes	Yes	Yes	Yes	Yes	Yes	Yes	Yes	Yes
Kambakamba et al.	2016	Yes	Yes	no	Yes	Yes	Yes	Yes	Yes	Yes
Terada et al.	2023	Yes	Yes	Yes	Yes	Yes	Yes	Yes	Yes	Yes
Liu et al.	2019	Yes	Yes	Yes	Yes	Yes	Yes	Yes	Yes	Yes
Martin et al.	2012	Yes	Yes	Yes	Yes	Yes	Yes	Yes	Yes	Yes
Turrini et al.	2006	Yes	Yes	Yes	no	Yes	unclear	unclear	unclear	unclear
Yuan et al.	2023	Yes	Yes	Yes	Yes	Yes	Yes	Yes	Yes	Yes
Thaler et al.	2012	Yes	Yes	Yes	Yes	Yes	unclear	unclear	Yes	Yes
Nakai et al.	2013	Yes	Yes	Yes	Yes	Yes	Yes	Yes	Yes	Yes
Cascinu et al.	2021	yes	yes	yes	yes	yes	unclear	yes	yes	yes
Agnelli et al.	2009	yes	yes	yes	no	yes	yes	yes	yes	yes
Sherman et al.	2015	yes	yes	yes	yes	yes	yes	yes	yes	yes
Maulat et al.	2020	yes	yes	yes	yes	yes	unclear	unclear	yes	yes
Reiss et al.	2022	yes	yes	yes	yes	yes	unclear	unclear	yes	yes
Ohashi et al.	2022	yes	yes	yes	yes	yes	yes	yes	yes	yes
Godinho et al.	2019	yes	yes	yes	yes	yes	yes	yes	yes	yes
Ko et al.	2015	yes	yes	yes	yes	yes	yes	yes	yes	yes
Conti et al.	2022	no	yes	yes	yes	yes	unclear	unclear	yes	yes
Gill et al.	2016	yes	yes	yes	yes	yes	yes	yes	yes	yes
Kharofa et al.	2019	yes	yes	yes	yes	yes	yes	yes	yes	yes
Khorana et al	2007	yes	yes	yes	yes	yes	yes	unclear	yes	unclear
Khorana et al	2013	yes	yes	yes	yes	yes	yes	yes	yes	unclear
Wagner et al.	2007	yes	yes	yes	yes	yes	unclear	unclear	yes	yes

Ko et al.	2008	yes	yes	yes	yes	yes	unclear	unclear	yes	yes
Walker et al.	2013	yes	yes	yes	yes	yes	yes	yes	yes	unclear
Berger et al.	2017	yes	yes	yes	yes	yes	unclear	unclear	yes	yes
Mahipal et al.	2018	yes	yes	yes	yes	yes	yes	yes	yes	yes
Maraveyas et al.	2012	yes	yes	yes	yes	yes	unclear	unclear	yes	yes
Mier-Hicks et al.	2018	yes	yes	yes	yes	yes	Unclear	yes	yes	unclear
Petrillo et al.	2019	yes	yes	yes	yes	yes	unclear	unclear	unclear	unclear
Suleman et al.	2019	yes	yes	yes	yes	yes	yes	yes	yes	yes
Ay et al.	2015	yes	yes	yes	yes	yes	unclear	unclear	yes	unclear
Dittrich et al.	2019	yes	yes	yes	yes	yes	yes	yes	yes	yes
Sharon et al.	2022	yes	yes	yes	yes	yes	unclear	unclear	unclear	unclear
Frere et al.	2020	yes	yes	yes	yes	yes	yes	yes	yes	yes
Lin et al.	2019	yes	yes	yes	yes	yes	unclear	unclear	unclear	unclear
Cardin et al.	2014	yes	yes	yes	yes	yes	unclear	unclear	unclear	yes
Zhen et al.	2016	yes	yes	yes	yes	yes	unclear	unclear	unclear	yes
Li et al.	2014	yes	yes	yes	yes	yes	unclear	unclear	unclear	unclear
Assenat et al.	2019	yes	yes	yes	yes	yes	yes	yes	yes	yes
Martín-Martos et al.	2017	yes	yes	yes	yes	yes	yes	yes	yes	yes
Moik et al.	2021	yes	yes	yes	yes	yes	unclear	unclear	unclear	unclear
Ullenhag et al.	2015	yes	yes	yes	yes	yes	yes	yes	yes	yes
Beatty et al.	2013	yes	yes	no	yes	yes	yes	yes	yes	yes
Middleton et al.	2014	yes	yes	yes	yes	yes	yes	yes	yes	yes
Tuinmann et al.	2004	yes	yes	yes	yes	yes	yes	yes	yes	yes
Van Buren Ii et al.	2013	yes	yes	yes	yes	yes	yes	yes	yes	yes
Chew et al.	2006	yes	yes	yes	yes	yes	yes	yes	yes	yes
Kindler et al.	2010	yes	yes	yes	yes	yes	yes	yes	yes	yes
Yhim et al.	2013	yes	yes	yes	yes	yes	yes	yes	yes	yes
Astsaturov et al.	2011	yes	yes	yes	yes	yes	yes	yes	yes	yes
Chau et al.	2006	yes	yes	yes	yes	yes	yes	yes	yes	yes
Gade al.	2017	yes	yes	yes	yes	yes	yes	yes	yes	yes
Aparicio et al.	2014	yes	yes	yes	yes	yes	yes	yes	yes	yes

Bendell et al.	2015	yes	yes	yes	yes	yes	yes	yes	yes	yes
Hoffman et al.	1998	yes	yes	yes	yes	yes	unclear	unclear	unclear	yes
Spano et al.	2012	yes	yes	no	yes	yes	yes	unclear	unclear	yes
Infante et al.	2011	yes	yes	yes	yes	yes	unclear	unclear	unclear	unclear
Kim et al.	2018	yes	yes	yes	yes	yes	yes	yes	yes	yes
Blom et al.	2006	yes	yes	yes	yes	yes	yes	yes	yes	yes
Rad et al.	2020	yes	yes	yes	yes	yes	unclear	unclear	unclear	unclear
Barrau et al.	2021	yes	yes	yes	yes	yes	unclear	unclear	yes	yes
Lambert et al.	2016	yes	yes	yes	yes	yes	yes	yes	yes	yes
Mita et al.	2016	yes	yes	yes	yes	yes	unclear	unclear	unclear	unclear
Ouaissi et al	2015	yes	yes	yes	yes	yes	yes	yes	yes	yes
Cardillo et al.		yes	yes	yes	no	yes	unclear	unclear	yes	yes
Khalaf et al.	2023	yes	yes	yes	yes	yes	yes	yes	yes	yes
Nso et al.	2023	yes	yes	yes	yes	yes	unclear	unclear	yes	yes
Starling et al.	2009	yes	yes	no	yes	yes	unclear	unclear	unclear	yes
Chan et al.	2018	yes	yes	yes	yes	yes	unclear	unclear	yes	yes
Stein et al.	2006	yes	yes	yes	yes	yes	unclear	unclear	yes	yes
Eijgenraam et al.	2013	yes	yes	yes	yes	yes	unclear	unclear	yes	no
Ghaneh et al.	2023	yes	yes	yes	yes	yes	unclear	yes	yes	no
Grierson et al.	2020	yes	yes	yes	yes	yes	unclear	yes	yes	yes
Han et al.	2021	yes	yes	yes	yes	yes	unclear	unclear	yes	yes
Hanna-Sawires et al.	2021	yes	yes	yes	yes	yes	yes	unclear	yes	yes
Ramanathan et al.	2019	yes	yes	yes	yes	yes	unclear	yes	yes	yes
García Adrián et al.	2022	yes	yes	yes	yes	yes	yes	unclear	yes	yes
Kondo et al.	2018	yes	yes	yes	yes	yes	unclear	unclear	no	yes
Kruger et al.	2017	yes	yes	yes	yes	yes	unclear	unclear	yes	yes
Hingorani et al.	2018	yes	yes	yes	yes	yes	unclear	yes	yes	unclear
Yoon et al.	2018	yes	yes	yes	yes	yes	yes	yes	yes	yes
Dragovich et al.	2014	yes	yes	yes	yes	yes	unclear	yes	no	yes
Okusaka et al.	2023	yes	yes	yes	yes	yes	unclear	unclear	yes	yes

Chew et al.	2015	yes	yes	yes	yes	yes	yes	unclear	yes	yes
Yamai et al. Ikezawa et al.	2022	yes	yes	yes	yes	yes	yes	yes	yes	yes
Nakai et al.	2011	yes	yes	yes	yes	yes	unclear	yes	no	yes
Nakai et al.	2013	yes	yes	yes	yes	yes	unclear	unclear	yes	yes
Hung et al.	2018	yes	yes	yes	yes	yes	yes	yes	yes	yes
Nagy et al.	2012	yes	yes	yes	yes	yes	unclear	unclear	no	unclear
Schwartzberg et al.	2017	yes	yes	yes	yes	yes	yes	yes	yes	yes
Riedl et al.	2014	yes	yes	yes	yes	yes	yes	yes	yes	yes
Infante et al.	2013	yes	yes	yes	yes	yes	yes	yes	yes	yes
Ko et al.	2016	yes	yes	yes	yes	yes	yes	yes	yes	yes
Picozzi et al.	2015	yes	yes	yes	yes	yes	yes	unclear	yes	yes
Herman et al.	2008	yes	yes	yes	yes	yes	yes	unclear	yes	yes
Epstein et al.	2011	yes	yes	yes	yes	yes	yes	yes	yes	yes
O'Reilly et al.	2010	yes	yes	yes	yes	yes	yes	unclear	yes	yes
Xiong	2004	yes	yes	yes	yes	yes	yes	yes	yes	yes
Spanheimer et al.	2014	yes	yes	yes	yes	yes	yes	yes	yes	yes
Spano et al.	2008	yes	yes	yes	yes	yes	yes	yes	yes	yes
Pelzer et al.	2009	yes	yes	yes	yes	yes	yes	yes	yes	yes
Pandit	2019	yes	yes	yes	yes	yes	yes	unclear	yes	yes
Müller et al.	2007	yes	yes	yes	yes	yes	yes	unclear	yes	yes
Faris	2013	yes	yes	yes	yes	yes	yes	yes	yes	yes
Conroy et al.	2018	yes	yes	yes	yes	yes	yes	yes	yes	yes
Conroy et al.	2011	yes	yes	yes	yes	yes	yes	yes	yes	yes
Loehrer et al.	2011	yes	yes	yes	yes	yes	yes	yes	yes	yes
Heinemann et al.	2000	yes	yes	yes	yes	yes	yes	yes	yes	yes
Kindler et al.	2010	yes	yes	yes	yes	yes	yes	yes	yes	yes
Haddock et al.	2007	yes	yes	yes	yes	yes	yes	yes	yes	yes
Burris et al.	1997	yes	yes	yes	yes	yes	yes	yes	yes	yes
Chou et al.	2020	yes	yes	yes	no	yes	yes	yes	yes	yes
Martin	2014	yes	yes	yes	yes	yes	yes	yes	yes	yes
Vogel et al.	2017	yes	yes	yes	yes	yes	yes	unclear	yes	yes

Lima et al.	2004	yes	yes	yes	yes	yes	yes	yes	yes	yes
Icli et al.	2007	yes	yes	yes	yes	yes	yes	yes	yes	yes
Reni et al.	2013	yes	yes	yes	yes	yes	yes	yes	yes	yes
Kim et al.	2013	yes	yes	yes	yes	yes	yes	yes	yes	yes
Philip et al.	2020	yes	yes	yes	yes	yes	yes	yes	yes	yes
Miyamoto et al.	2010	yes	yes	yes	yes	yes	yes	yes	yes	yes
Safran et al.	2001	yes	yes	yes	yes	yes	yes	yes	yes	yes
Mamon et al.	2010	yes	yes	yes	yes	yes	yes	yes	yes	yes
Ko et al.	2011	yes	yes	yes	yes	yes	yes	yes	yes	yes
Ko et al.	2010	yes	yes	yes	yes	yes	yes	yes	yes	yes
Rich et al.	2004	yes	yes	yes	yes	yes	yes	yes	yes	yes
Small et al.	2011	yes	yes	yes	yes	yes	yes	yes	yes	yes
Van Cutsem et al.	2009	yes	yes	yes	yes	yes	yes	yes	yes	yes
Van Cutsem et al.	2004	yes	yes	yes	yes	yes	yes	yes	yes	yes
Poplin et al.	2009	yes	yes	yes	yes	yes	yes	yes	yes	yes
Pisters et al.	2000	yes	yes	yes	yes	yes	yes	unclear	yes	yes
Varadhachary	2008	yes	yes	yes	yes	yes	yes	yes	yes	yes
Evans et al.	2008	yes	yes	yes	yes	yes	yes	yes	yes	yes
Reni et al.	2018	yes	yes	yes	yes	yes	yes	yes	yes	yes
Colucci et al.	2010	yes	yes	yes	yes	yes	yes	yes	yes	yes
Hurwitz et al.	2015	yes	yes	yes	yes	yes	yes	yes	yes	yes
Martin et al.	2015	yes	yes	yes	yes	yes	yes	unclear	yes	yes

1. Was the sample frame appropriate to address the target population?
2. Were study participants sampled in an appropriate way?
3. Was the sample size adequate?
4. Were the study subjects and the setting described in detail?

5. Was the data analysis conducted with sufficient coverage of the identified sample?
6. Were valid methods used for the identification of the condition?
7. Was the condition measured in a standard, reliable way for all participants?
8. Was there appropriate statistical analysis?
9. Was the response rate adequate, and if not, was the low response rate managed appropriately?

Table S4. Baseline caracteristis of included studies.

Author	*Year of publication*	*Study Type*	*Country*	*Total number of patients*	*Median age*	*Mean age*	*TNM -stage*	*Proportion of female population (%)*	*Reported oucome*
Navi et al. (1)	2017	retrospective cohort study	USA	12279	NA	77	all stages	63	incidence
Baluka et al.(2)	2016	observational study	Poland	35	NA	63	all stages	NA	prevalence
Huang et al. (3)	2022	retrospective analysis	China	48	NA	57,26	all stages	52	prevalence
Lee et al. (4)	2016	retrosepctive analysis	South Korea	1761	NA	NA	all stages	41	prevalence
Ryan et al.(5)	2018	retrospective cohort study	USA	29	74	NA	NA	62	prevalence
Shen et al.(6)	2022	retrospective cohort study	China	210	NA	60	all stages	NA	prevalence
Tonnesen et al.(7)	2023	retrospective cohort study	Denmark	11351	71,3	NA	NA	50	prevalence and incidence
Sogaard et al. (8)	2016	prospective cohort study	Denmark	116	63	NA	NA	48	prevalence
Anker et al. (9)	2016	prospective cohort study	Germany	72	NA	59	NA	36	prevalence
Ratib et al. (10)	2016	retrospective cohort study	England	78579	NA	NA	NA	NA	incidence

Goncalves et al. (11)	2012	randomized control trial	France	52	64	NA	III/IV	40	incidence
Le Bozec et al.(12)	2023	retrospective cohort study	France	182	65,73	NA	III/IV	45	prevalence
Navi et al.(13)	2015	retrospective cohort study	USA	16571	NA	78	all stages	NA	prevalence and incidence
Navi et al.(14)	2018	retrospective cohort study	USA	16386	NA	NA	NA	NA	prevalence
Faille et al.(15)	2018	prospective cohort study	France	42	66	NA	NA	38	prevalence
Fogelman et al.(16)	2011	randomized control trial	USA	47	59	NA	III/IV	30	incidence
Li et al.(17)	2015	retrospective cohort study	USA	670	62	NA	all stages	41	incidence
Yip et al.(18)	2005	randomised controlled trial	USA, Canada , New Zeland	173	NA	NA	III/IV	NA	incidence
Couturaud et al.(19)	2023	retrospective study	France	22242	70	69,7	all stages	49	prevalence and incidence
Stitzel et al.(20)	2023	retrospective cohort study	USA	478	67	NA	NA	51	prevalence
Kindler et al.(21)	2011	randomised control trial	USA, UK<br>Amsterdam, France,<br>Canada,Japan, South Korea	308	61	NA	III/IV	39	incidence
Lee et al.(22)	2016	retrospective cohort study	South Korea	1115	64,6	NA	all stages	41	incidence
Javle et al.(23)	2009	randomized control trial	USA	50	64	NA	III/IV	56	incidence

Jiang et al.(24)	2017	retrospective cohort study	USA	1783	59,6	NA	all stages	43	prevalence
Cai et al.(25)	2019	retrospective cohort study	China	98	56,5	NA	III/IV	42	prevalence
Tingle et al.(26)	2020	retrospective cohort study	UK	164	NA	NA	III/IV	46	prevalence
Corrie et al.(27)	2020	randomized control trial	Ireland	142	66	NA	III/IV	43	incidence
Rosemurgy et al.(28)	2008	randomized control trial	USA	30	70	68	III/IV	NA	incidence
Menapace et al.(29)	2011	retrospective cohort study	USA	135	NA	65,9	all stages	43	prevalence
Huang et al.(30)	2023	retrospective observational study	USA	242903	NA	67,8	NA	50	prevalence
Ishigaki et al.(31)	2017	retrospective cohort study	Japan	475	67	NA	III/IV	42	prevalence
Pant et al.(32)	2016	randomized control trial	USA	163	59,2	NA	III/IV	48	incidence
Zhai et al.(33)	2019	retrospective cohort study	China	9	NA	63,3	NA	22	prevalence
Rivera et al.(34)	2019	randomized control trial	Spain, Switzerland	20	73	NA	III/IV	60	incidence
Sgori et al.(35)	2015	retrospective cohort study	USA	147	NA	NA	III/IV	49	prevalence
Martin et al.(36)	2012	randomized control trial	USA	42	60	NA	III/IV	48	incidence
Wun et al.(37)	2009	observational study	USA	6524	NA	NA	NA	NA	incidence

Chen et al.(38)	2018	retrospective cohort study	Taiwan	838	62	NA	III/IV	41	prevalence and incidence
Suzuki et al.(39)	2021	retrospective cohort study	Finnland	432	NA	70,7	all stages	49	prevalence
Laderman et al.(40)	2023	retrospective cohort study	USA	400	66	NA	III/IV	48	prevalence
Jeong et al.(41)	2023	retrospective, observational cohort study	South Korea	170	64	NA	III/IV	42	incidence
Ruff et al.(42)	2019	retrospective cohort study	USA	18095	66	NA	NA	54	incidence
Oh et al.(43)	2008	retrospective cohort study	South Korea	75	67	NA	III/IV	41	incidence
Mandala et al.(44)	2007	retrospective cohort study	Italy	227	63	NA	III/IV	47	incidence
Krepline et al.(45)	2016	prospective cohort study	USA	234	64	NA	NA	49	incidence
Lyman et al.(46)	2013	retrospective cohort study	USA	27479	NA	NA	NA	49	incidence
Kim et al.(47)	2016	retrospective cohort study	South Korea	499	NA	63,19	all stages	68	prevalence
Rich et al.(48)	2012	retrospective cohort study	USA	185	62	NA	III/IV	46	incidence
Katz et al.(49)	2016	prospective cohort study	USA	22	64	NA	NA	55	incidence
Zhao et al.(50)	2022	retrospective cohort study	China	49	NA	NA	NA	59	incidence
Alberts et al.(51)	2005	randomized control trial	USA	43	67	NA	III/IV	41	incidence

O'Reilly et al.(52)	2020	randomized control trial	USA, Canada, Israel	117	65	NA	III/IV	56	incidence
Infante et al.(53)	2014	randomized controlled trial	USA, South Korea, Taiwan	80	64	NA	III/IV	47	incidence
Rougier et al.(54)	2013	randomized controlled trial	USA,France, Germany,Czech Republic,Belgium, Italy	345	61	NA	III/IV	42	prevalence and incidence
Friess et al.(55)	2006	randomized control trial	Germany,Belgium,Spain,Austria	44	68	NA	III/IV	51	incidence
El-Khoueriry et al.(56)	2012	randomized control trial	USA	52	66	NA	III/IV	40	incidence
Noel et al.(57)	2022	randomized control trial	USA	48	NA	10,4	III/IV	47	incidence
Burtness et al.(58)	2016	randomized control trial	USA	91	60	NA	III/IV	30	incidence
He et al. (59)	2023	retrospective cohort study	China	2663	NA	NA	NA	49	incidence
Nakai et al.(60)	2010	retrospective cohort study	Japan	155	NA	NA	III/IV	46	prevalence
Kamarajah et al.(61)	2021	retrospective cohort study	UK	690	NA	NA	all stages	49	prevalence
Rocha et al.(62)	2016	prospective cohort study	USA	43	62	NA	II	40	prevalence
Kambakamba et al.(63)	2016	prospective cohort study	USA	10	NA	NA	III/IV	33	incidence
Terada et al.(64)	2023	retrospective case-control study	Japan	35	NA	69,7	III/IV	49	prevalence

Liu et al.(65)	2019	prospective cohort study	China	54	61	NA	III/IV	52	incidence
Martin et al.(36)	2012	prospective cohort study	USA	27	61	NA	III/IV	48	prevalence and incidence
Turrini et al.(66)	2006	retrospective cohort study	France	26	NA	NA	III	NA	incidence
Yuan et al.(67)	2023	prospective cohort study	UK	115	NA	NA	NA	54	incidence
Thaler et al.(68)	2012	prospective cohort study	Austria	132	65	NA	all stages	60	incidence
Nakai et al.(69)	2013	randomized control trial	Japan	35	69	NA	III/IV	49	incidence
Cascinu et al.(70)	2021	randomized control trial	Italy	236	NA	NA	III/IV	56	incidence
Agnelli et al.(71)	2009	randomized control trial	Italy	53	NA	NA	III/IV	52	incidence
Sherman et al.(72)	2015	prospective cohort study	USA	45	NA	64	III/IV	58	incidence
Maulat et al.(73)	2020	prospective cohort study	France	703	70	NA	all stages	45	prevalence
Reiss et al.(74)	2022	randomized control trial	USA	91	NA	NA	III/IV	42	incidence
Ohashi et al.(75)	2022	prospective cohort study	Japan	1006	69	67,6	II and III/IV	44	prevalence
Godinho et al.(76)	2019	retrospective cohort study	Portugal	165	73	NA	all stages	45	incidence
Ko et al.(77)	2015	randomized control trial	USA	46	67	NA	III/IV	NA	incidence

Conti et al.(78)	2022	retrospective cohort study	France	159	80	NA	III/IV	52	Prevalence
Gill et al.(79)	2016	randomized control trial	Canada	102	NA	NA	III/IV	44	incidence
Kharofa et al.(80)	2019	randomized control trial	USA	18	66	NA	all stages	50	incidence
Khorana et al.(81)	2007	retrospective cohort study	USA	26118	NA	64	NA	49	incidence
Khorana et al.(82)	2013	retrospective cohort study	USA	1336	NA	64	NA	NA	incidence
Wagner et al.(83)	2007	randomized control trial	USA	45	63,8	NA	III/IV	38	incidence
Ko et al.(84)	2008	randomized control trial	USA	52	60	NA	IV	56	incidence
Walker et al.(85)	2013	retrospective cohort study	UK	83203	NA	NA	NA	NA	incidence
Berger et al.(86)	2017	retrospective cohort study	Germany	132	NA	NA	III/IV	38	incidence
Mahipal et al.(87)	2018	randomized control trial	USA	24	68	NA	III/IV	36	incidence
Maraveyas et al.(88)	2012	randomized control trial	UK	121	63	NA	III/IV	41	incidence
Mier-Hicks et al.(89)	2018	retrospective cohort study	USA	95	NA	62,9	all stages	54	prevalence
Petrillo et al.(90)	2019	retrospective cohort study	Italy	64	69,5	NA	III/IV	64	incidence
Suleman et al.(91)	2019	prospective cohort study	Canada	26	58,2	NA	NA	NA	incidence

Ay et al.(92)	2015	prospective obs. study	Austria	99	NA	NA	NA	NA	incidence
Dittrich et al.itt(93)	2019	randomized control trial	Austria	30	NA	63,9	III/IV	50	incidence
Sharon et al.(94)	2022	retrospective cohort study	USA	28888	68	NA	all stages	49	prevalence
Frere et al.(95)	2020	prospective, observational study	France	731	69	NA	NA	42	incidence
Lin et al.(96)	2019	randomized control trial	USA	11	65	NA	all stages	18	incidence
Cardin et al.(97)	2014	randomized control trial	Taiwan , USA	38	71	NA	III/IV	49	incidence
Zhen et al.(98)	2016	randomized control trial	USA	11	61	NA	III/IV	50	incidence
Li et al.(99)	2014	randomized control trial	USA	19	NA	64	IV	63	incidence
Assenat et al.(100)	2021	randomized control trial	France	62	62	NA	IV	40	incidence
Martín-Martos et al.(101)	2017	retrospective observational study	Spain, France, Italy	517	NA	NA	NA	NA	incidence
Moik et al.(102)	2021	prospective observational cohort study	Austria	145	66	NA	all stages	54	prevalence and incidence
Ullenhag et al.(103)	2015	randomized control trial	Sweden	12	64	NA	IV	NA	incidence
Beatty et al.(104)	2013	randomized control trial	USA	16	59	NA	III/IV	36	incidence
Middleton et al.(105)	2014	randomized control trial	UK	1062	62	NA	III/IV	43	incidence

Tuinmann et al.(106)	2004	randomized control trial	Germany	52	58	NA	II and III/IV	38	incidence
Van Buren li et al.(107)	2013	randomized control trial	Romania	58	60	NA	I/II/III	48	incidence
Chew et al.(108)	2006	retrospective cohort study	USA	5289	71	NA	all stages	52	incidence
Kindler et al.(109)	2010	randomized control trial	USA	540	63,7	NA	III/IV	45	incidence
Yhim et al.(110)	2013	retrospective cohort study	South Korea	63	65	NA	III/IV	46	incidence
Astsaturov et al.(111)	2011	randomized control trial	USA	16	67	NA	IV	65	incidence
Chau et al.(112)	2006	randomized control trial	UK	95	64	NA	III/IV	43	incidence
Gade al.(113)	2017	prospective cohort study	Denmark , Norway	402	59	NA	all stages	49	incidence
Aparicio et al.(114)	2014	randomized control trial	Spain	12	59	NA	III/IV	58	incidence
Bendell et al.(115)	2015	randomized control trial	USA, UK	66	63	NA	NA	45	incidence
Hoffman et al.(116)	1998	randomized control trial	USA	95	NA	NA	NA	49	prevalence and incidence
Spano et al.(117)	2012	randomized control trial	USA, Canada	8	59	NA	III/IV	12	incidence
Infante et al.(118)	2011	randomized control trial	USA	22	62	NA	IV	30	incidence

Kim et al.(119)	2018	retrospective cohort study	South Korea	216	63	NA	IV	36	incidence
Blom et al.(120)	2006	retrospective cohort study	Netherlands	202	64	NA	NA	43	prevalence
Rad et al.(121)	2020	retrospective study	Iran	348	NA	59,67	NA	39	prevalence
Barrau et al.(122)	2021	retrospective study	France	174	67	NA	III/IV	45	incidence
Lambert et al.(123)	2016	retrospective study	France	142	61	60	III/IV	42	prevalence
Mita et al.(124)	2016	randomized control trial	USA	16	61	NA	III/IV	42	incidence
Ouaissi et al.(125)	2015	retrospective study	France	162	69	NA	all stages	44	incidence
Cardillo et al.(126)	2018	retrospective study	USA	298	NA	NA	NA	49	incidence
Khalaf et al.(127)	2023	retrospective cohort study	USA	6342	69	NA	all stages	3	prevalence
Nso et al.(128)	2023	retrospective cohort study	USA	34744	NA	NA	NA	NA	incidence
Starling et al.(129)	2009	randomized control trial	UK	20	63	NA	III/IV	50	prevalence
Chan et al.(130)	2018	retrospective cohort cohort study	Taiwan	7479	NA	64,7	all stages	41	incidence
Stein et al.(131)	2006	retrospective cohort study	USA	1176000	NA	NA	NA	NA	incidence
Eijgenraam et al.(132)	2013	Retrospectove cohort study	Netherlands	448	NA	61	NA	NA	prevalence

Ghaneh et al.(133)	2023	randomized control trial	UK, Germany	78	61	NA	NA	19	incidence
Grierson et al.(134)	2020	randomized control trial	USA	16	66	73	III/IV	56	prevalence
Han et al.(135)	2021	retrospective cohort study	USA	12785	71,9	NA	I/II	46	incidence
Hanna-Sawires et al.(136)	2021	retrospective cohort study	Netherlands	361	64	72	I/II and III/IV	48	incidence
Ramanathan et al.(137)	2019	randomized control trial	USA	42	66	NA	III/IV	40	prevalence
García Adrián et al.(138)	2022	retrospective cohort study	Spain	666	65	NA	all stages	42	incidence
Kondo et al.(139)	2018	retrospective cohort study	Japan	103	65	NA	III/IV	46	incidence
Kruger et al.(140)	2017	retrospective cohort study	Germany	127	63,2	NA	III/IV	41	incidence
Hingorani et al.(141)	2018	randomized control trial	USA	279	NA	65	IV	46	prevalence
Yoon et al.(142)	2018	retrospective cohort study	South Korea	505	65,1	NA	III/IV	42	incidence
Dragovich et al.(143)	2014	randomized control trial	USA	25	63	NA	III/IV	NA	prevalence
Okusaka et al.(144)	2023	retrospective cohort study	Japan	1006	69	67,7	II and III/IV	44	prevalence
Chew et al.(145)	2015	retrospective cohort study	Taiwan	618	NA	NA	NA	NA	incidence

Yamai et al.(146)	2022	retrospective cohort study	Japan	157	63	NA	III/IV	48	incidence
Nakai et al.(147)	2012	randomized control trial	Japan	14	60	NA	III/IV	NA	prevalence
Nakai et al.(148)	2013	retrospective cohort study	Japan	250	66	NA	III/IV	49	prevalence
Hung et al.(149)	2018	retrospective cohort study	Taiwan	838	62	NA	III/IV	41	prevalence
Nagy et al.(150)	2012	retrospective cohort study	Hungary	36	68	NA	all stages	58	incidence
Schwartzberg et al.(151)	2017	randomized control trial	USA	80	65	NA	II and III/IV	51	incidence
Riedl et al.(152)	2014	prospective, observational cohort study	Austria	1840	62	NA	NA	46	incidence
Infante et al.(153)	2013	randomized control trial	UK,USA	72	64	NA	III/IV	32	incidence
Ko et al.(154)	2016	randomized control trial	USA	15	NA	58,5	NA	33	incidence
Picozzi et al.(155)	2015	randomized control trial	USA	58	62	NA	III/IV	43	incidence
Herman et al.(156)	2008	prospective cohort study	USA	616	NA	6.68	I/II/III	48	prevalence
Epstein et al.(157)	2011	prospective cohort study	USA	1915	NA	66	II/III/IV	49	prevalence
O'Reilly et al.(158)	2010	randomized control trial	USA	74	65	NA	III/IV	45	incidence
Xiong et al.(159)	2004	randomized control trial	USA	41	NA	61,1	III/IV	39	incidence

Spanheimer et al.(160)	2014	retrospectice cohort rtudy	USA	34	61	NA	III/IV	44	incidence
Spano et al.(161)	2008	randomized control trial	USA,Spain,Canada,UK ,Italy	113	65	NA	III/IV	41	prevalence
Pelzer et al.(162)	2015	randomized control trial	Germany	152	63	NA	III/IV	41	incidence
Pandit et al.(163)	2019	prospective cohort study	USA	11	63	NA	III	NA	prevalence
Müller et al.(164)	2007	prospective cohort study	Germany	136	63	NA	III/IV	41	prevalence
Faris et al.(165)	2013	retrospectice cohort rtudy	USA	22	63	NA	III	41	incidence
Conroy et al.(166)	2018	randomized control trial	France, Canada	481	63	NA	all stages	44	incidence
Conroy et al.(167)	2011	randomized control trial	France, Canada	335	61	NA	IV	60	incidence
Loehrer et al.(168)	2011	randomized control trial	USA	69	66	NA	III	48	incidence
Heinemann et al.(169)	2000	randomized control trial	Germany	39	57	NA	III/IV	39	incidence
Kindler et al.(170)	2012	randomized control trial	USA	17	62	NA	III/IV	55	incidence
Haddock et al.(171)	2007	randomized control trial	USA	48	68	NA	III	40	incidence
Burris et al.(172)	1997	randomized control trial	USA	63	62	NA	III/IV	46	incidence
Chou et al.(173)	2022	retrospectice cohort rtudy	Taiwan	709	63	NA	NA	45	incidence

Martin et al.(174)	2014	retrospectice cohort rtudy	Spain	84	63	NA	III/IV	50	prevalence and incidence
Vogel et al.(175)	2017	prospective cohort study	Netherlands, USA	132	NA	64	III	46	prevalence
Lima et al.(176)	2004	randomized control trial	USA,Italy, Canada,New Zeland	180	63	NA	III/IV	41	incidence
Icli et al.(177)	2007	prospective cohort study	Turkey	33	56	NA	III/IV	22	incidence
Reni et al.(178)	2013	randomized control trial	Italy	27	61	NA	IV	49	incidence
Kim et al.(179)	2013	randomized control trial	USA	71	64	NA	all stages	53	incidence
Philip et al.(180)	2020	randomized control trial	France, USA, Canada, Spain, Italy	106	65	NA	III	55	incidence
Miyamoto et al.(181)	2010	retrospectice cohort rtudy	USA	24	78	NA	I/II/III	66	incidence
Safran et al.(182)	2001	prospective cohort study	USA	44	64	NA	III	42	incidence
Mamon et al.(183)	2010	randomized control trial	USA	78	NA	62	III	50	incidence
Ko et al.(184)	2011	randomized control trial	USA	29	63	NA	III/IV	45	incidence
Ko et al.(185)	2010	randomized control trial	USA	36	60	NA	IV	44	incidence
Rich et al.(186)	2004	randomized control trial	USA	109	63	NA	III	53	incidence
Small et al.(187)	2011	retrospectice cohort study	USA	29	62	NA	I/II/III	38	incidence

Van Cutsem et al.(188)	2009	randomized control trial	USA	583	61	NA	IV	41	incidence
Van Cutsem et al.(189)	2004	randomized control trial	USA, Belgium, Czech Republic, Germany, Amsterdam, Poland, Austria, Netherlands,	673	61	NA	III/IV	43	incidence
Poplin et al.(190)	2009	randomized control trial	USA	824	63	NA	III/IV	47	prevalence
Pisters et al.(191)	2000	prospective cohort study	USA	308	63	NA	NA	55	prevalence
Varadhachary et al.(192)	2008	randomized control trial	USA	79	64	NA	NA	40	incidence
Evans et al.(193)	2008	randomized control trial	USA	86	NA	NA	I/II	NA	incidence
Reni et al.(194)	2018	randomized control trial	Italy	54	66	NA	NA	59	incidence
Colucci et al.(195)	2010	randomized control trial	Italy	189	63	NA	II/III/IV	41	incidence
Hurwitz et al.(196)	2015	randomized control trial	USA	119	66	NA	IV	NA	incidence
Martin et al.(197)	2015	prospective cohort study	USA	200	62	NA	III	49	prevalence

Table S5. Provided methods underlying the definitions of cardiovascular diseases

Navi et al.	2017	International Classification of Diseases (ICD)
Baluka et al.	2016	NA
Huang et al.	2022	NA
Lee et al.	2016	NA

Ryan et al.	2018	NA
Shen et al.	2022	NA
Tonnesen et al.	2023	ICD
Sogaard et al.	2016	ICD
Anker et al.	2016	All patients had a 12-lead ECG performed after 10 min of supine rest. ECGs were analysed using Welch Allyn software and adjudicated by two medical reviewers
Ratib et al.	2016	ICD
Goncalves et al.	2012	Common Terminology Criteria for Adverse Events (NCI-CTCAE) version 3.0
Le Bozec et al.	2023	NA
Navi et al.	2015	ICD
Navi et al.	2018	ICD
Faille et al.	2018	TE events were identified based on radiological evidence.
Fogelman et al.	2011	NCI-CTCAE version 3.0
Li et al.	2015	TE events were identified based on radiological evidence.
Yip et al.	2005	NA
Couturaud et al.	2023	ICD
Stitzel et al.	2023	TE events were identified based on radiological evidence.
Kindler et al.	2011	NCI-CTCAE
Lee et al.	2016	TE events were identified based on radiological evidence.
Javle et al.	2009	NCI-CTCAE version 3.0
Jiang et al.	2017	NA
Cai et al.	2019	NA
Tingle et al.	2020	NA
Corrie et al.	2020	NCI-CTCAE version 4.03
Rosemurgy et al.	2008	NCI-CTCAE
Menapace et al.	2011	TE events were identified based on radiological evidence.
Huang et al.	2023	ICD
Ishigaki et al.	2017	TE events were identified based on radiological evidence.
Pant et al.	2016	NCI-CTCAE
Zhai et al.	2019	NA
Rivera et al.	2019	NCI-CTCAE version 4.0
Sgori et al.	2015	NA

Martin et al.	2012	NA
Wun et al.	2009	NA
Chen et al.	2018	TE events were identified based on radiological evidence.
Suzuki et al.	2021	TE events were identified based on radiological evidence.
Laderman et al.	2023	TE events were identified based on radiological evidence.
Jeong et al.	2023	TE events were identified based on radiological evidence.
Ruff et al.	2019	NA
Oh et al.	2008	TE events were identified based on radiological evidence.
Mandala et al.	2007	ICD
Krepline et al.	2016	NA
Lyman et al.	2013	ICD
Kim et al.	2016	NA
Rich et al.	2012	NCI-CTCAE version 2.0
Katz et al.	2016	NCI-CTCAE version 4.0
Zhao et al.	2022	ICD
Alberts et al.	2005	NCI-CTCAE version 2.0
O'Reilly et al.	2020	NCI-CTCAE version 4.0
Infante et al.	2014	NCI-CTCAE version 4.0
Rougier et al.	2013	NCI-CTCAE version 3.0
Friess et al.	2006	NCI-CTCAE
El-Khoueriry et al.	2012	NA
Noel et al.	2022	NCI-CTCAE version 4.3
Burtness et al.	2016	NCI-CTCAE version 2.0
He et al.	2023	ICD
Nakai et al.	2010	NA
Kamarajah et al.	2021	NA
Rocha et al.	2016	NCI-CTCAE version 3.0
Kambakamba et al.	2016	NA
Terada et al.	2023	NA
Liu et al.	2019	NA
Martin et al.	2012	NA
Turrini et al.	2006	NA
Yuan et al.	2023	ICD

Thaler et al.	2012	TE events were identified based on radiological evidence.
Nakai et al.	2013	NCI-CTCAE version 3.0
Cascinu et al.	2021	NCI-CTCAE version 4.0
Agnelli et al.	2009	NA
Sherman et al.	2015	NA
Maulat et al.	2020	NA
Reiss et al.	2022	NCI-CTCAE version 5.0
Ohashi et al.	2022	TE events were identified based on radiological evidence.
Godinho et al.	2019	TE events were identified based on radiological evidence.
Ko et al.	2015	NCI-CTCAE
Conti et al.	2022	NA
Gill et al.	2016	NCI-CTCAE
Kharofa et al.	2019	NCI-CTCAE
Khorana et al.	2007	TE events were identified based on radiological evidence.
Khorana et al.	2013	ICD
Wagner et al.	2007	NCI-CTCAE
Ko et al.	2008	NCI-CTCAE version 2.0
Walker et al.	2013	VTE diagnosis was determined in the first instance from medical codes in the CPRD and HES
Berger et al.	2017	NCI-CTCAE
Mahipal et al.	2018	NCI-CTCAE version 4.0
Maraveyas et al.	2012	NCI-CTCAE version 3.0
Mier-Hicks et al.	2018	ICD
Petrillo et al.	2019	NCI-CTCAE version 4.3
Suleman et al.	2019	TE events were identified based on radiological evidence.
Ay et al.	2015	NA
Dittrich et al.	2019	NCI-CTCAE version 3.0
Sharon et al.	2022	NA
Frere et al.	2020	TE events were identified based on radiological evidence.
Lin et al.	2019	NCI-CTCAE version 3.0
Cardin et al.	2014	NCI-CTCAE version 3.0
Zhen et al.	2016	NCI-CTCAE version 4.0
Li et al.	2014	NCI-CTCAE version 4.0

Assenat et al.	2019	NCI-CTCAE version 3.0
Martín-Martos et al.	2017	NA
Moik et al.	2021	NA
Ullenhag et al.	2015	NCI-CTCAE version 3.0
Beatty et al.	2013	NCI-CTCAE version 3.0
Middleton et al.	2014	NCI-CTCAE version 3.0
Tuinmann et al.	2004	NCI-CTCAE version 2.0
Van Buren Ii et al.	2013	NCI-CTCAE version 4.0
Chew et al.	2006	TE events were identified based on radiological evidence.
Kindler et al.	2010	NCI-CTCAE version 3.0
Yhim et al.	2013	TE events were identified based on radiological evidence.
Astsaturov et al.	2011	NCI-CTCAE version 3.0
Chau et al.	2006	NCI-CTCAE version 2.0
Gade al.	2017	VTE were objectively confirmed by trained personnel by thorough review of medical records or discharge letters, biochemical investigations and diagnostic images
Aparicio et al.	2014	NCI-CTCAE version 3.0
Bendell et al.	2015	NCI-CTCAE version 3.0
Hoffman et al.	1998	Standard ECOG toxicity criteria
Spano et al.	2012	NCI-CTCAE
Infante et al.	2011	NCI-CTCAE version 3.0
Kim et al.	2018	TE events were identified based on radiological evidence.
Blom et al.	2006	NA
Rad et al.	2020	NA
Barrau et al.	2021	TE events were identified based on radiological evidence.
Lambert et al.	2016	TE events were identified based on radiological evidence.
Mita et al.	2016	NCI-CTCAE version 4.0
Ouaissi et al.	2015	TE events were identified based on radiological evidence.
Cardillo et al.	2018	NA
Khalaf et al.	2023	ICD
Nso et al.	2023	NA
Starling et al.	2009	NCI-CTCAE version 3.0
Chan et al.	2018	ICD

Stein et al.	2006	ICD
Eijgenraam et al.	2013	NA
Ghaneh et al.	2023	NCI-CTCAE version 4.0
Grierson et al.	2020	NA
Han et al.	2021	ICD
Hanna-Sawires et al.	2021	NA
Ramanathan et al.	2019	NA
García Adrián et al.	2022	NA
Kondo et al.	2018	TE events were identified based on radiological evidence.
Kruger et al.	2017	NA
Hingorani et al.	2018	NCI-CTCAE version 4.3
Yoon et al.	2018	TE events were identified based on radiological evidence.
Dragovich et al.	2014	NCI-CTCAE version 3.0
Okusaka et al.	2023	TE events were identified based on radiological evidence.
Chew et al.	2015	ICD
Yamai et al.	2022	NCI-CTCAE version 5.0
Nakai et al.	2011	NCI-CTCAE version 3.0
Nakai et al.	2013	NCI-CTCAE version 3.0
Hung et al.	2018	Myocardial infarction was confirmed using the third universal definition, including increased levels of cardiac enzymes combined with either clinical symptoms of angina, abnormal electrocardiogram, or identification of thrombus by coronary angiography. Ischemic stroke was defined as a sudden or rapid onset of neurological deficit that was confirmed by a neurologist and neuroimaging with either computed tomography scan or magnetic resonance imaging.
Nagy et al.	2012	NA
Schwartzberg et al.	2017	NCI-CTCAE version 4.0
Riedl et al.	2014	TE events were identified based on radiological evidence.
Infante et al.	2013	NCI-CTCAE version 3.0
Ko et al.	2016	NCI-CTCAE version 4.0
Picozzi et al.	2015	NCI-CTCAE version 4.0
Herman et al.	2008	NA
Epstein et al.	2011	ICD
O'Reilly et al.	2010	NCI-CTCAE version 3.0
Xiong et al.	2004	NCI-CTCAE version 2.0
Spanheimer et al.	2014	NCI-CTCAE version 4.0

Spano et al.	2008	NCI-CTCAE version 3.0
Pelzer et al.	2009	NCI-CTCAE
Pandit	2019	NA
Müller et al.	2007	NA
Faris et al.	2013	NA
Conroy et al.	2018	NCI-CTCAE version 4.0
Conroy et al.	2011	NCI-CTCAE version 4.0
Loehrer et al.	2011	NA
Heinemann et al.	2000	WHO grade
Kindler et al.	2010	NCI-CTCAE version 3.0
Haddock et al.	2007	NCI-CTCAE version 2.0
Burris et al.	1997	WHO criteria for toxicities
Chou et al.	2020	ICD
Martin et al.	2014	NCI-CTCAE version 3.0
Vogel et al.	2017	Clavien-Dindo classification
Lima et al.	2004	NCI-CTCAE version 2.0
Icli et al.	2007	WHO grading
Reni et al.	2013	NCI-CTCAE version 3.0
Kim et al.	2013	NA
Philip et al.	2020	NCI-CTCAE version 4.0
Miyamoto et al.	2010	ACE-27 comorbidity index
Safran et al.	2001	Cancer and Leukemia Group B (CALGB) toxicity criteria
Mamon et al.	2010	CALGB
Ko et al.	2011	NCI-CTCAE version 3.0
Ko et al.	2010	NCI-CTCAE version 2.0
Rich et al.	2004	Common Toxicity Criteria (CTEP) for acute reactions, and the RTOG late scoring system for late reactions
Small et al.	2011	NCI-CTCAE version 3.0
Van Cutsem et al.	2009	NCI-CTCAE version 3.0
Van Cutsem et al.	2004	NCI-CTCAE version 2.0
Poplin et al.	2009	NCI-CTCAE version 2.0
Pisters et al.	2000	WHO grading
Varadhachary et al.	2008	NCI-CTCAE version 3.0

Evans et al.	2008	NCI-CTCAE version 2.0
Reni et al.	2018	NCI-CTCAE version 4.0
Colucci et al.	2010	NCI-CTCAE version 2.0
Hurwitz et al.	2015	NCI-CTCAE version 4.0
Martin et al.	2015	NA

NA: not available, TE: trombotic event, VTE: venous thromboembolic event ECG: echocardiograpy

Table S6. Therapeutic agents in treatments associated with **A:** small molecule inhibitors **B:** monoclonal antibodies.

First Author	Year of publication	Small molecule inhibitors

Goncalves et al.	2012	Sorafenib
Kindler et al.	2011	Axitinib
Infante et al.	2014	Trametinib
El-Khoueriry et al.	2012	Sorafenib
Noel et al.	2022	SM-88
Cardin et al.	2014	Sorafenib+Erlotinib
Zhen et al.	2016	Cabozantinib
Assenat et al.	2019	Erlotinib
Aparicio et al.	2011	Sorafenib
Spano et al.	2012	Axitinib
O'Reilly et al.	2010	Sunitinib
Spano et al.	2008	Axitinib
Kindler et al.	2010	Sorafenib
Reni et al.	2013	Sunitinib
Ko et al.	2010	Erlotinib
Van Cutsem et al.	2009	Erlotinib
Hurwitz et al.	2015	Ruxolitinib

A:

B:

**First Author**	**Year of publication**	**Monoclonal antibodies**
Fogelman et al.	2011	Bevacizumab
Javle et al.	2009	Bevacizumab
Pant et al.	2016	Bevacizumab
Martin et al.	2012	Bevacizumab
Alberts et al.	2005	PS-341
Dittrich et al.	2019	Bevacizumab
Lin et al.	2019	Oregovomab
Beatty et al.	2013	CP-870,983
Van Buren et al.	2013	Bevacizumab
Kindler et al.	2010	Bevacizumab
Astsaturov et al.	2011	Bevacizumab
Astsaturov et al.	2011	Bevacizumab
Mita et al.	2016	CEP-37250/KHK2804
Picozzi et al.	2015	Clivatuzumab tetraxetan
Ko et al.	2011	Cetuximab + Bevacizumab
Ko et al.	2010	Bevacizumab
Small et al.	2011	Bevacizumab
Van Cutsem et al.	2009	Bevacizumab

Figure S1. Prevalence of **A:** hypertension **B:** ischemic heart disease **C:** heart failure **D:** arrhythmia **E**: stroke among pancreatic cancer patients.

CI: confidence interval.

A:

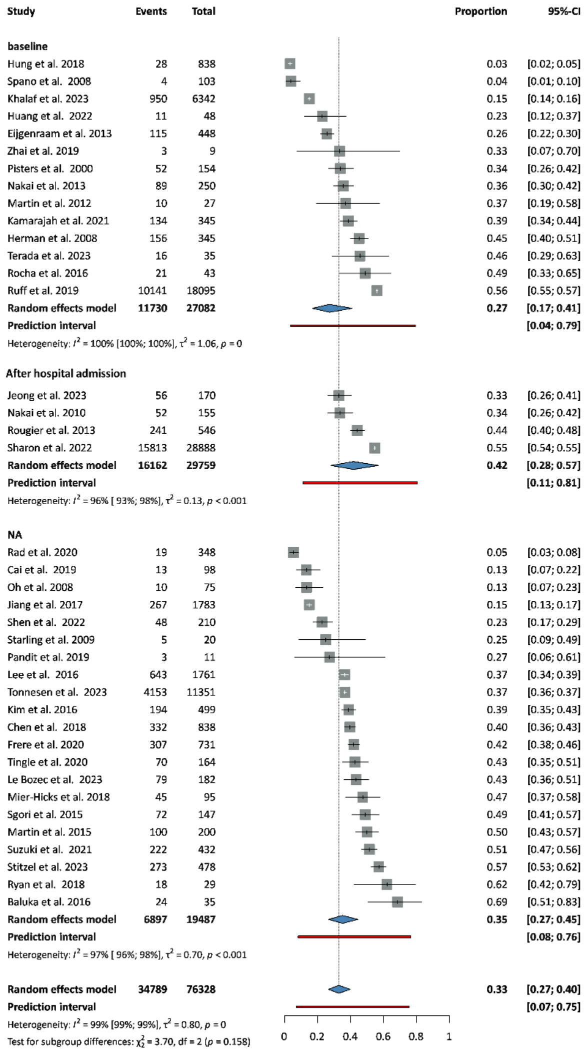

B:

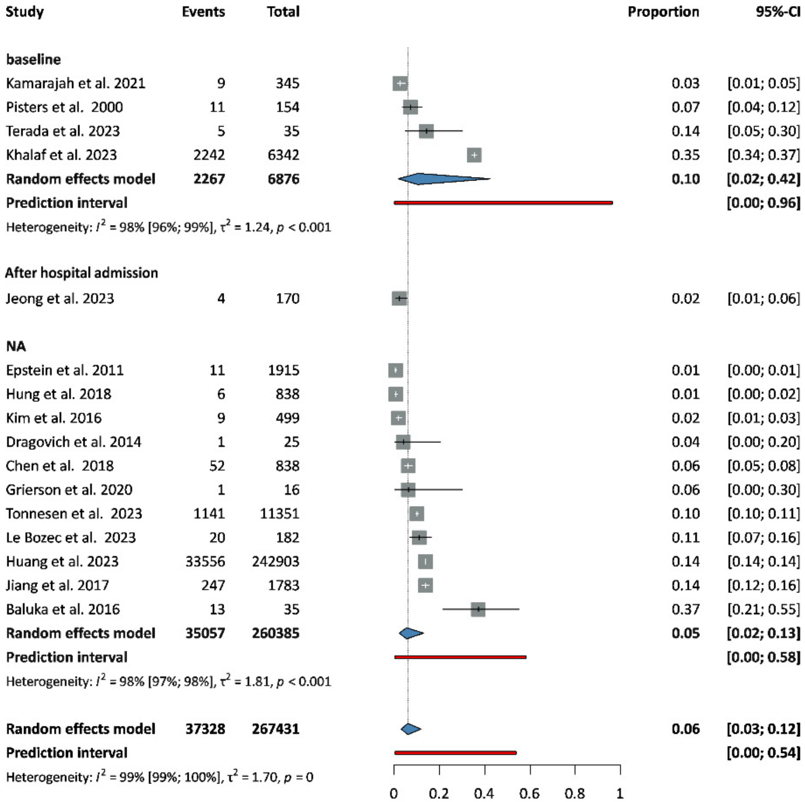

C:

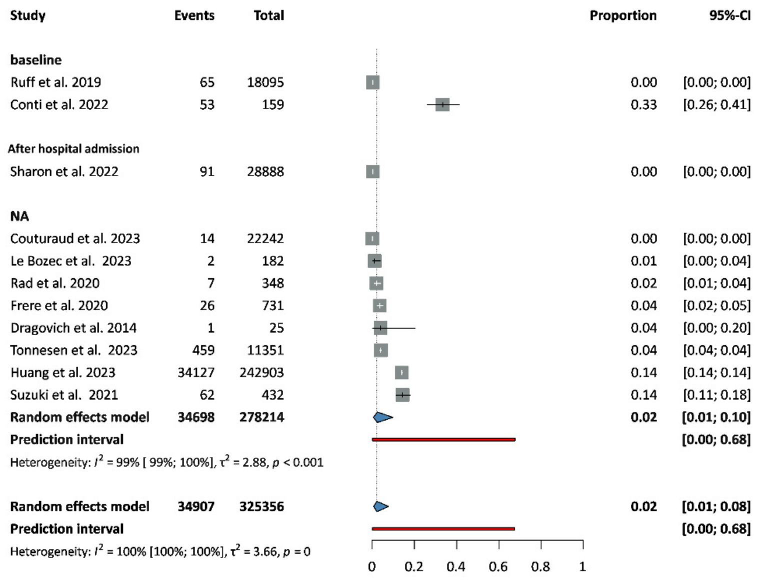

Study Events Total Proportion 95%-CI
baseline
Ruff et al. 2019 65 18095 0.00 [0.00; 0.00]
Conti et al. 2022 53 159 0.33 [0.26; 0.41]
After hospital admission
Sharon et al. 2022 91 28888 0.00 [0.00; 0.00]
NA
Couturaud et al. 2023 14 22242 0.00 [0.00; 0.00]
Le Bozec et al. 2023 2 182 0.01 [0.00; 0.04]
Rad et al. 2020 7 348 0.02 [0.01; 0.04]
Frere et al. 2020 26 731 0.04 [0.02; 0.05]
Dragovich et al. 2014 1 25 0.04 [0.00; 0.20]
Tonnesen et al. 2023 459 11351 0.04 [0.04; 0.04]
Huang et al. 2023 34127 242903 0.14 [0.14; 0.14]
Suzuki et al. 2021 62 432 0.14 [0.11; 0.18]
Random effects model 34698 278214 0.02 [0.01; 0.10]
Prediction interval [0.00; 0.68]
Heterogeneity: I^2 = 99% [99%; 100%], τ^2 = 2.88, p < 0.001
Random effects model 34907 325356 0.02 [0.01; 0.08]
Prediction interval [0.00; 0.68]
Heterogeneity: I^2 = 100% [100%; 100%], τ^2 = 3.66, p = 0
0 0.2 0.4 0.6 0.8 1

D:

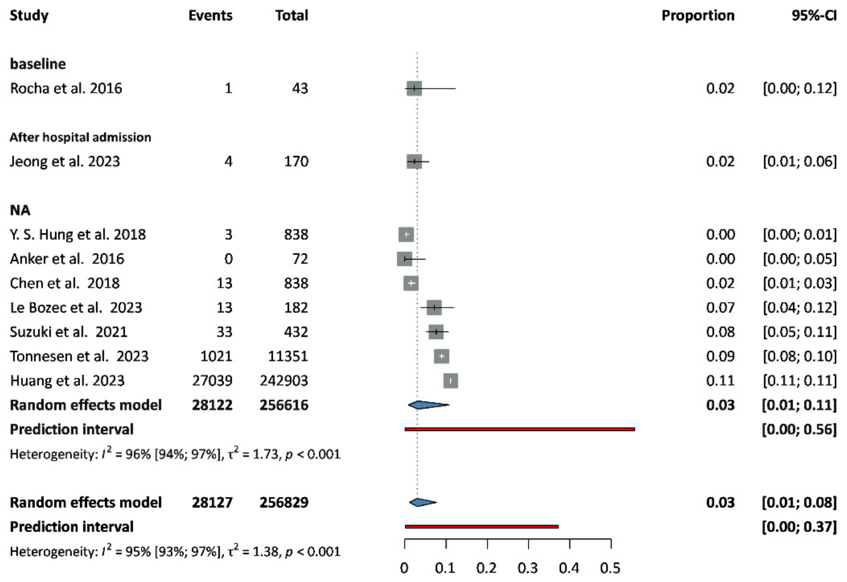

Study Events Total Proportion 95%-CI
baseline
Rocha et al. 2016 1 43 0.02 [0.00; 0.12]
After hospital admission
Jeong et al. 2023 4 170 0.02 [0.01; 0.06]
NA
Y. S. Hung et al. 2018 3 838 0.00 [0.00; 0.01]
Anker et al. 2016 0 72 0.00 [0.00; 0.05]
Chen et al. 2018 13 838 0.02 [0.01; 0.03]
Le Bozec et al. 2023 13 182 0.07 [0.04; 0.12]
Suzuki et al. 2021 33 432 0.08 [0.05; 0.11]
Tonnesen et al. 2023 1021 11351 0.09 [0.08; 0.10]
Huang et al. 2023 27039 242903 0.11 [0.11; 0.11]
Random effects model 28122 256616 0.03 [0.01; 0.11]
Prediction interval [0.00; 0.56]
Heterogeneity: I^2 = 96% [94%; 97%], τ^2 = 1.73, p < 0.001
Random effects model 28127 256829 0.03 [0.01; 0.08]
Prediction interval [0.00; 0.37]
Heterogeneity: I^2 = 95% [93%; 97%], τ^2 = 1.38, p < 0.001
0 0.1 0.2 0.3 0.4 0.5

E:

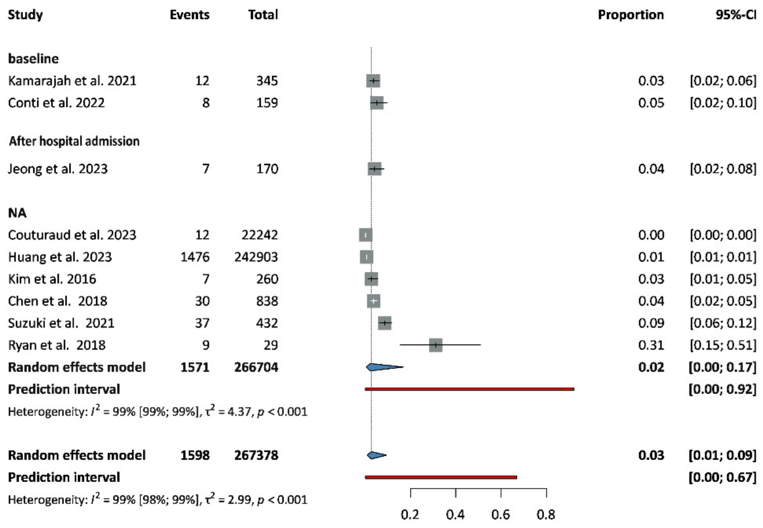

Study Events Total Proportion 95%-CI
baseline
Kamarajah et al. 2021 12 345 0.03 [0.02; 0.06]
Conti et al. 2022 8 159 0.05 [0.02; 0.10]
After hospital admission
Jeong et al. 2023 7 170 0.04 [0.02; 0.08]
NA
Couturaud et al. 2023 12 22242 0.00 [0.00; 0.00]
Huang et al. 2023 1476 242903 0.01 [0.01; 0.01]
Kim et al. 2016 7 260 0.03 [0.01; 0.05]
Chen et al. 2018 30 838 0.04 [0.02; 0.05]
Suzuki et al. 2021 37 432 0.09 [0.06; 0.12]
Ryan et al. 2018 9 29 0.31 [0.15; 0.51]
Random effects model 1571 266704 0.02 [0.00; 0.17]
Prediction interval [0.00; 0.92]
Heterogeneity: I^2 = 99% [99%; 99%], τ^2 = 4.37, p < 0.001
Random effects model 1598 267378 0.03 [0.01; 0.09]
Prediction interval [0.00; 0.67]
Heterogeneity: I^2 = 99% [98%; 99%], τ^2 = 2.99, p < 0.001
0.2 0.4 0.6 0.8

Figure S2. Incidence of hypertension among patients **A:** with TNM III-IV **B:** who received Gemcitabine-based therapy **C:** treatment in association with small molecule inhibitors **D:** in association with monoclonal antibodies**.** CI: confidence interval.

A:

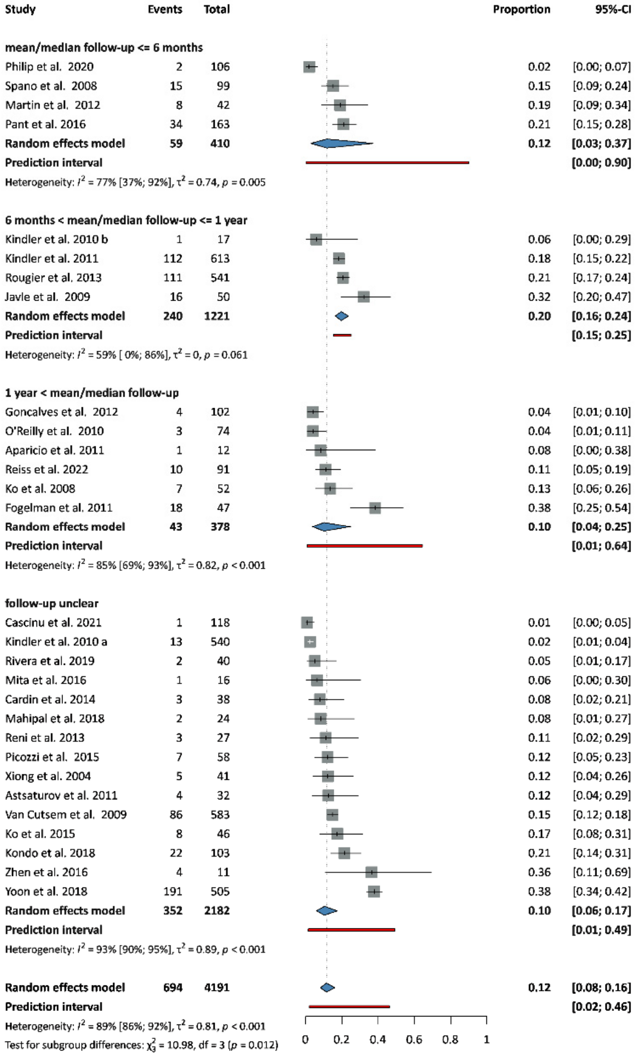

B:

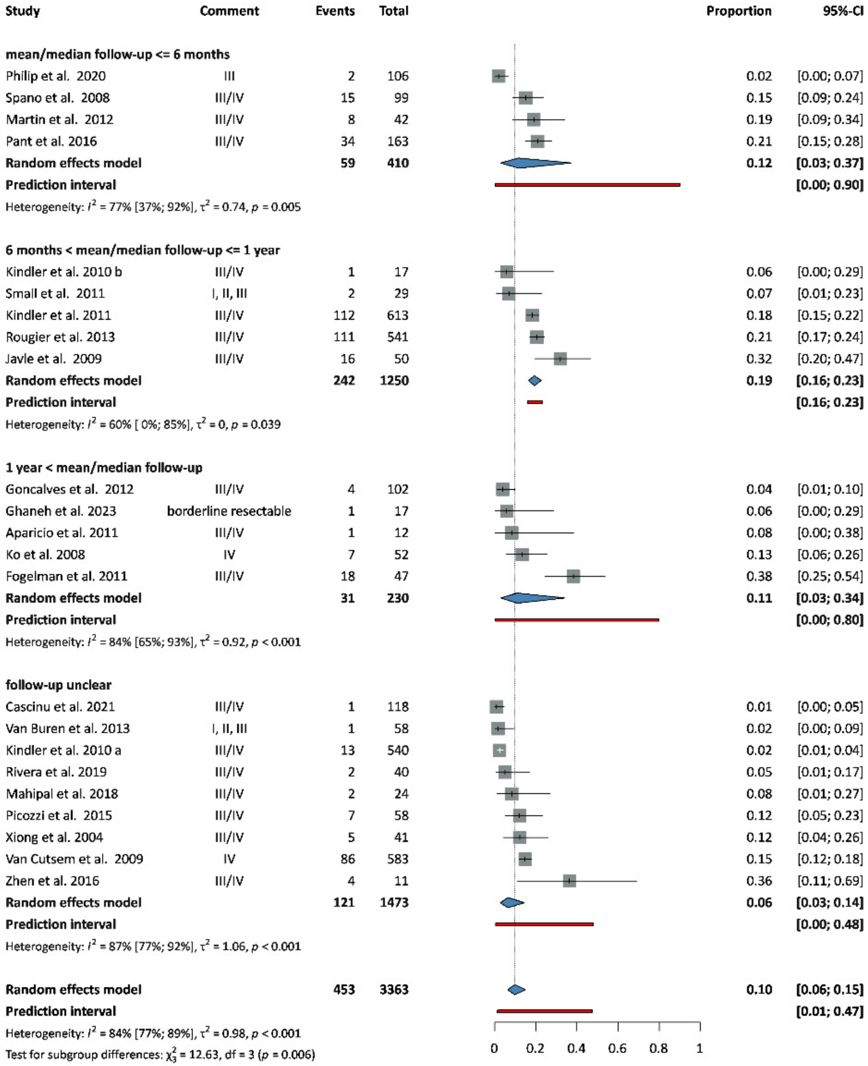

C:

Study	Events	Total	Proportion	95%-CI
**mean/median follow-up <= 6 months**				
Spano et al. 2008	15	68	0.22	[0.13; 0.34]
**6 months < mean/median follow-up <= 1 year**				
Kindler et al. 2010 b	1	17	0.06	[0.00; 0.29]
Kindler et al. 2011	85	305	0.28	[0.23; 0.33]
**1 year < mean/median follow-up**				
Goncalves et al. 2012	2	50	0.04	[0.00; 0.14]
O'Reilly et al. 2010	3	74	0.04	[0.01; 0.11]
Aparicio et al. 2011	1	12	0.08	[0.00; 0.38]
**Random effects model**	**6**	**136**	**0.04**	**[0.01; 0.22]**
**Prediction interval**				**[0.00; 0.90]**
Heterogeneity: I^2 = 0% [0%; 90%], τ^2 = 0, p = 0.795				
**follow-up unclear**				
Cardin et al. 2014	3	38	0.08	[0.02; 0.21]
Reni et al. 2013	3	27	0.11	[0.02; 0.29]
Van Cutsem et al. 2009	86	583	0.15	[0.12; 0.18]
Zhen et al. 2016	4	11	0.36	[0.11; 0.69]
**Random effects model**	**96**	**659**	**0.15**	**[0.11; 0.20]**
**Prediction interval**				**[0.10; 0.22]**
Heterogeneity: I^2 = 43% [0%; 81%], τ^2 = 0, p = 0.153				
**Random effects model**	**203**	**1185**	**0.12**	**[0.07; 0.21]**
**Prediction interval**				**[0.02; 0.45]**
Heterogeneity: I^2 = 80% [64%; 89%], τ^2 = 0.52, p < 0.001				

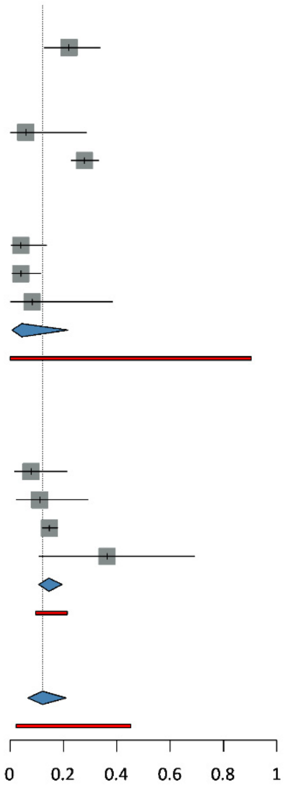

D:

Study	Comment	Events	Total	Proportion	95%-CI
**mean/median follow-up <= 6 months**					
Martin et al. 2012	III/IV	8	42	0.19	[0.09; 0.34]
Pant et al. 2016	III/IV	34	163	0.21	[0.15; 0.28]
**6 months < mean/median follow-up <= 1 year**					
Small et al. 2011	I, II, III	2	29	0.07	[0.01; 0.23]
Javle et al. 2009	III/IV	16	50	0.32	[0.20; 0.47]
**1 year < mean/median follow-up**					
Fogelman et al. 2011	III/IV	18	47	0.38	[0.25; 0.54]
**follow-up unclear**					
Van Buren et al. 2013	I, II, III	1	58	0.02	[0.00; 0.09]
Kindler et al. 2010 a	III/IV	10	277	0.04	[0.02; 0.07]
Mita et al. 2016	III/IV	1	16	0.06	[0.00; 0.30]
Picozzi et al. 2015	III/IV	7	58	0.12	[0.05; 0.23]
Astsaturov et al. 2011	IV	4	32	0.12	[0.04; 0.29]
Van Cutsem et al. 2009	IV	60	296	0.20	[0.16; 0.25]
**Random effects model**		**83**	**737**	**0.08**	**[0.03; 0.19]**
**Prediction interval**					**[0.01; 0.49]**
Heterogeneity: I^2 = 86% [72%; 93%], τ^2 = 0.61, p < 0.001					
**Random effects model**		**161**	**1068**	**0.13**	**[0.07; 0.23]**
**Prediction interval**					**[0.02; 0.56]**
Heterogeneity: I^2 = 84% [73%; 90%], τ^2 = 0.80, p < 0.001					

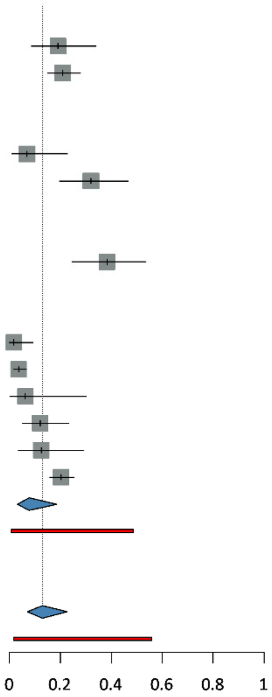

Figure S3. Incidence of ischemic heart disease among patients **A:** with TNM III-IV **B:** who received Gemcitabine-based therapy **C:** treatment in association with monoclonal antibodies. CI: confidence interval.

A:

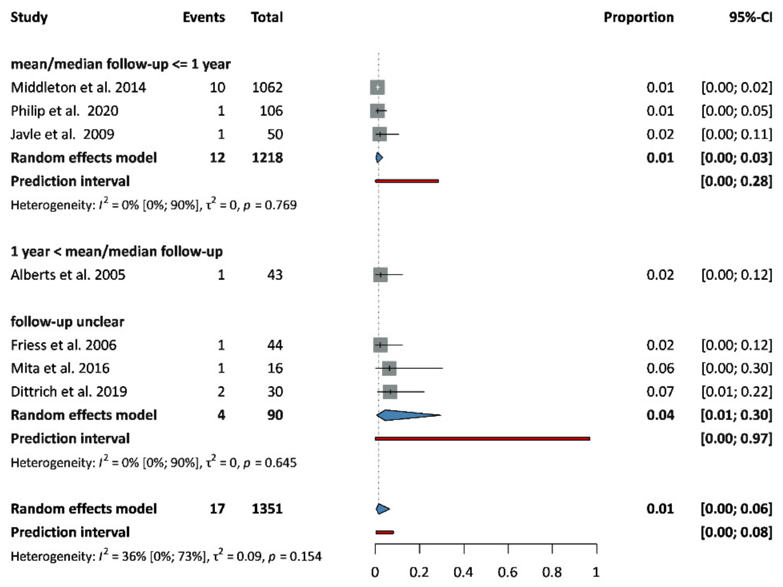

B:

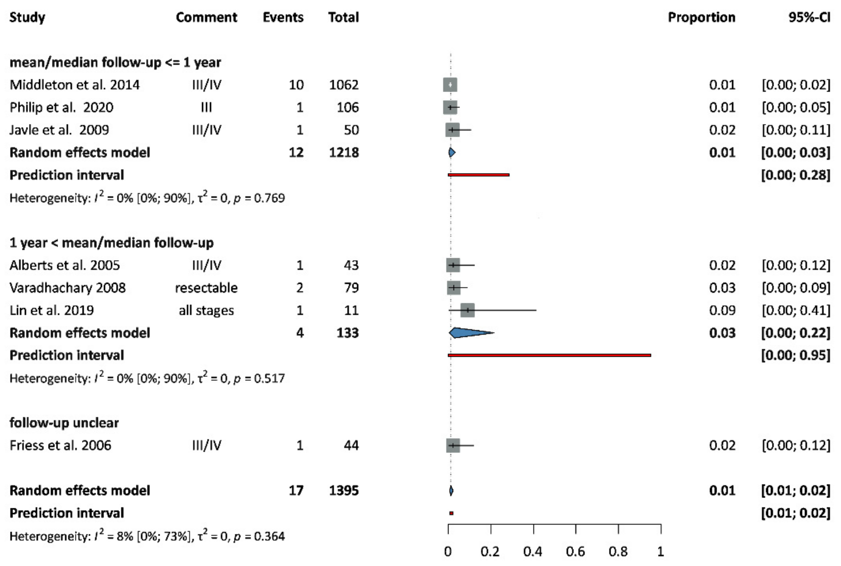

C:

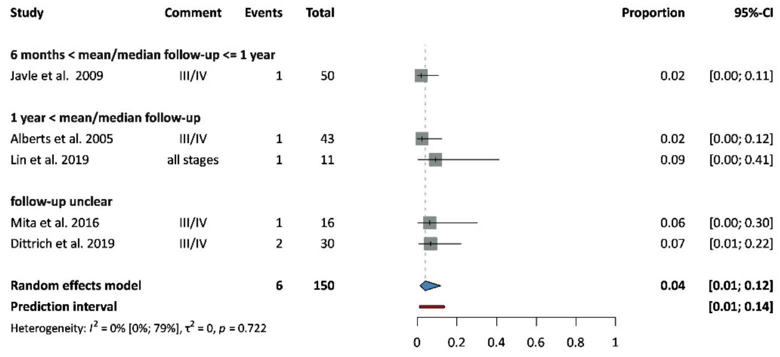

Figure S4. Incidence of stroke among patients **A:** with TNM III-IV **B:** who received Gemcitabine-based therapy **C:** treatment in association with small molecule inhibitors **D:** in association with monoclonal antibodies. CI: confidence interval.

A:

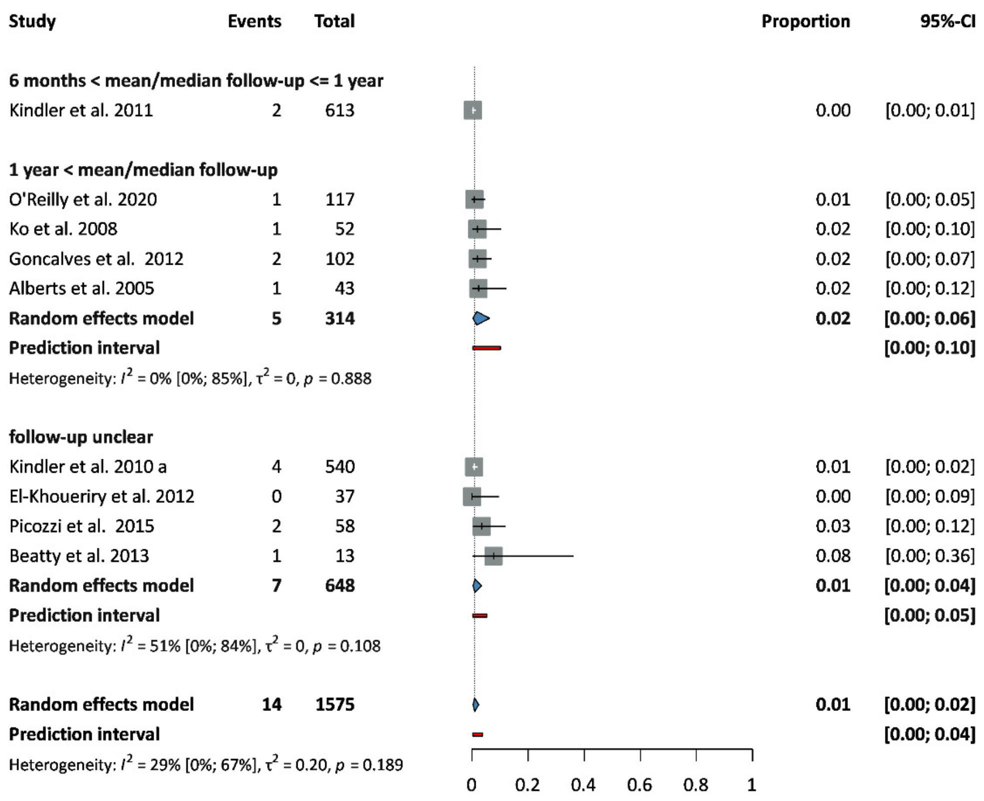

B:

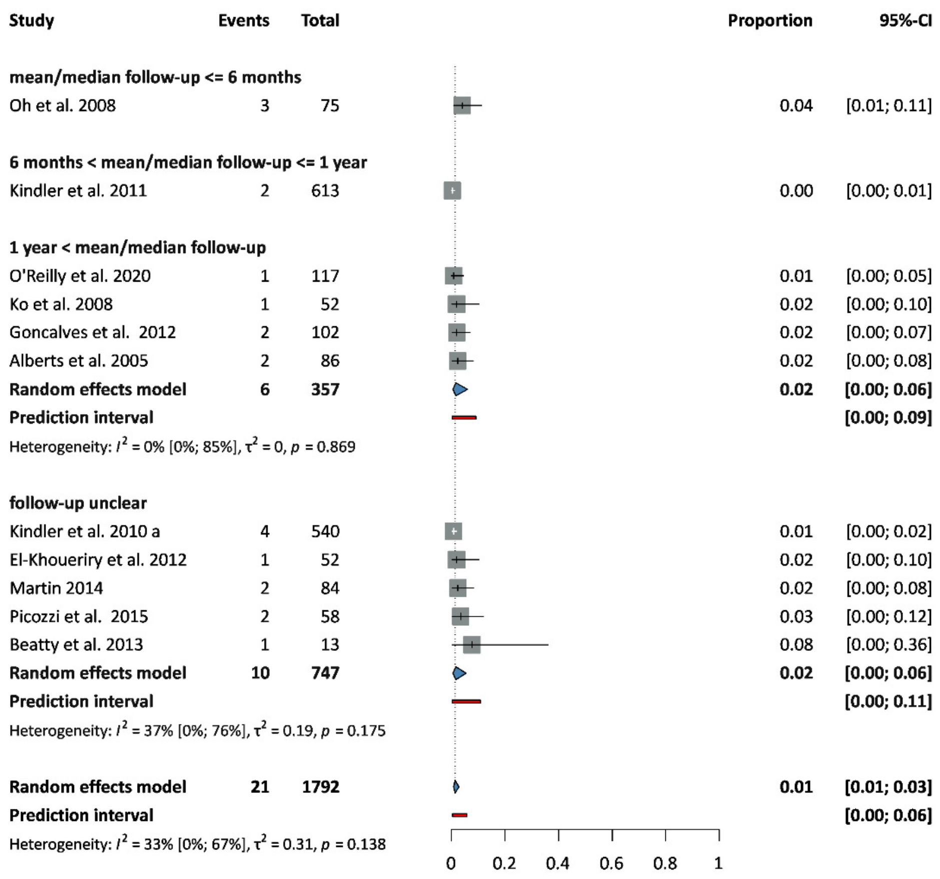

C:

Study	Events	Total	Proportion	95%-CI
**6 months < mean/median follow-up <= 1 year**				
Kindler et al. 2011	1	305	0.00	[0.00; 0.02]
**1 year < mean/median follow-up**				
Goncalves et al. 2012	1	50	0.02	[0.00; 0.11]
**follow-up unclear**				
El-Khoueriry et al. 2012	1	52	0.02	[0.00; 0.10]
**Random effects model**	**3**	**407**	**0.01**	**[0.00; 0.08]**
**Prediction interval**				**[0.00; 0.92]**

Heterogeneity: $I^2 = 7\%$ [0%; 90%], $\tau^2 = 0$, $p = 0.341$

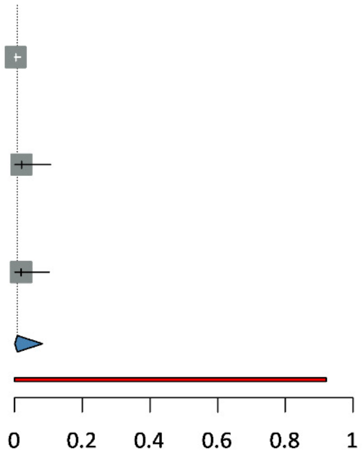

D:

Study	Events	Total	Proportion	95%-CI
**1 year < mean/median follow-up**				
Alberts et al. 2005	2	86	0.02	[0.00; 0.08]
**follow-up unclear**				
Kindler et al. 2010 a	2	277	0.01	[0.00; 0.03]
Picozzi et al. 2015	2	58	0.03	[0.00; 0.12]
Beatty et al. 2013	1	13	0.08	[0.00; 0.36]
**Random effects model**	**5**	**348**	**0.02**	**[0.00; 0.22]**
**Prediction interval**				**[0.00; 1.00]**
Heterogeneity: $I^2 = 56\%$ [0%; 87%], $\tau^2 = 0.30$, $p = 0.104$				
**Random effects model**	**7**	**434**	**0.02**	**[0.00; 0.07]**
**Prediction interval**				**[0.00; 0.18]**

Heterogeneity: $I^2 = 34\%$ [0%; 77%], $\tau^2 = 0.12$, $p = 0.209$

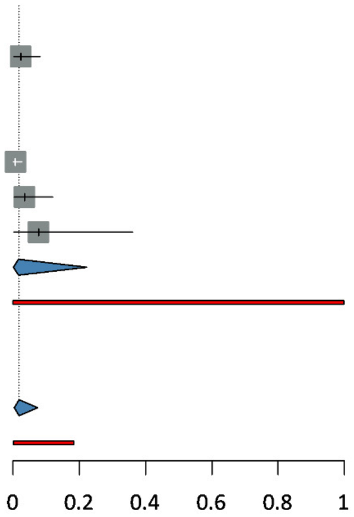

Figure S5. Incidence of arrhythmia among patients **A:** with TNM III-IV **B:** who received Gemcitabine-based therapy. CI: confidence interval.

A:

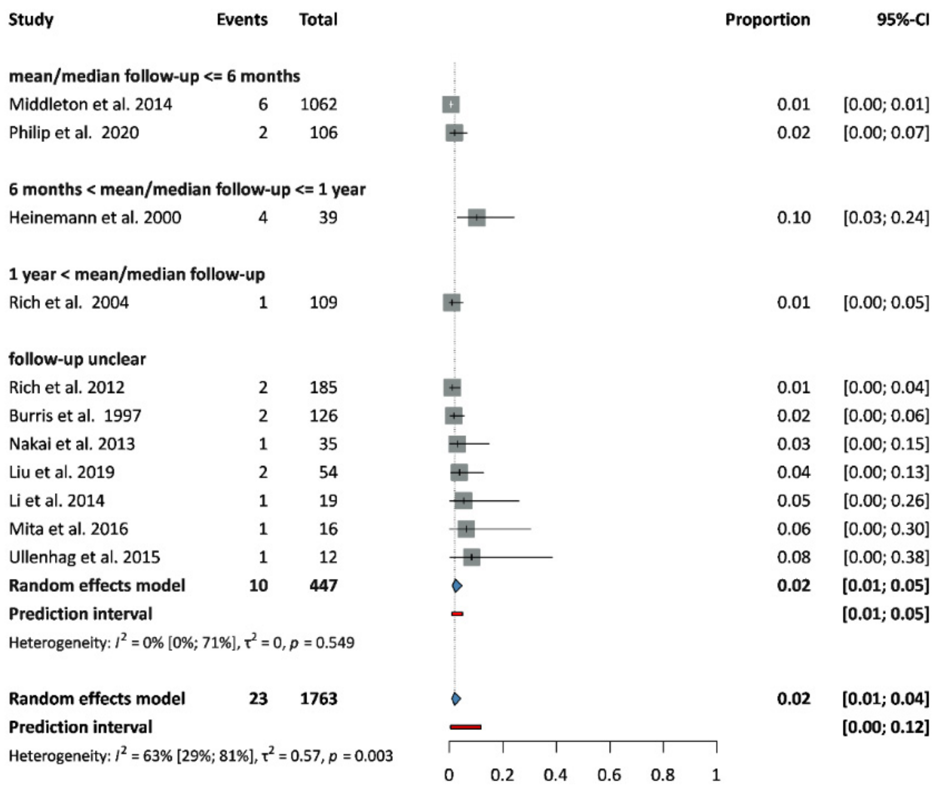

B:

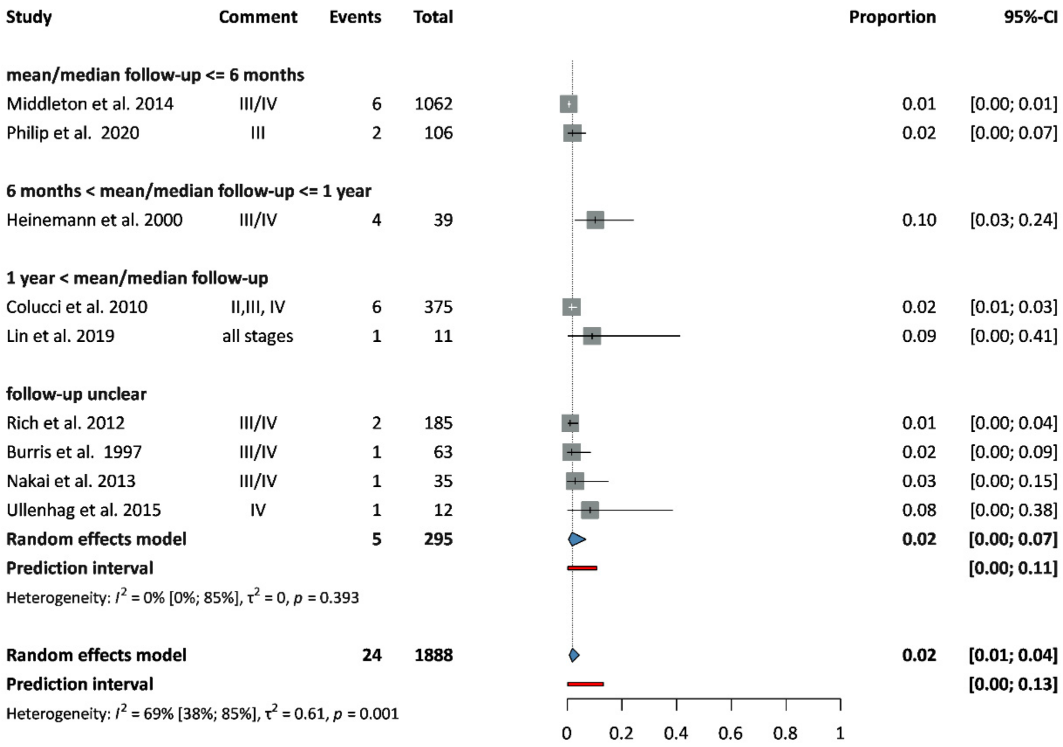

Figure S6. Incidence of cardiac failure among patients who received Gemcitabine-based therapy. CI: confidence interval.

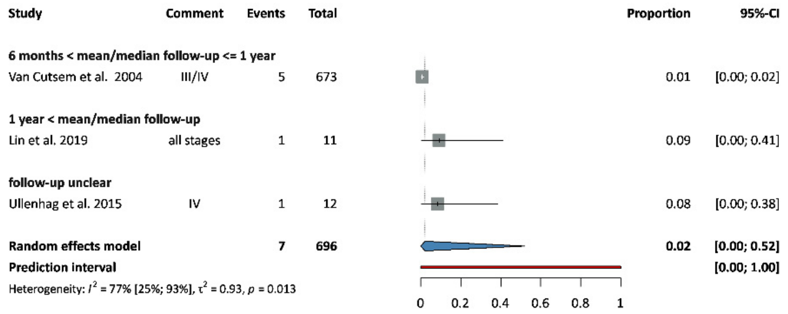

Figure S7. Incidence of hypotension among patients **A:** with TNM III-IV **B:** who received Gemcitabine-based therapy. CI: confidence interval.

A:

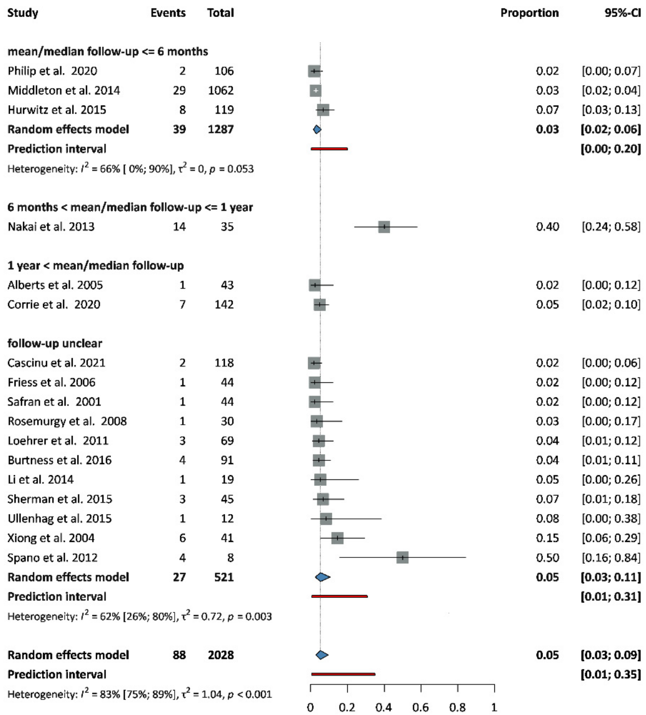

B:

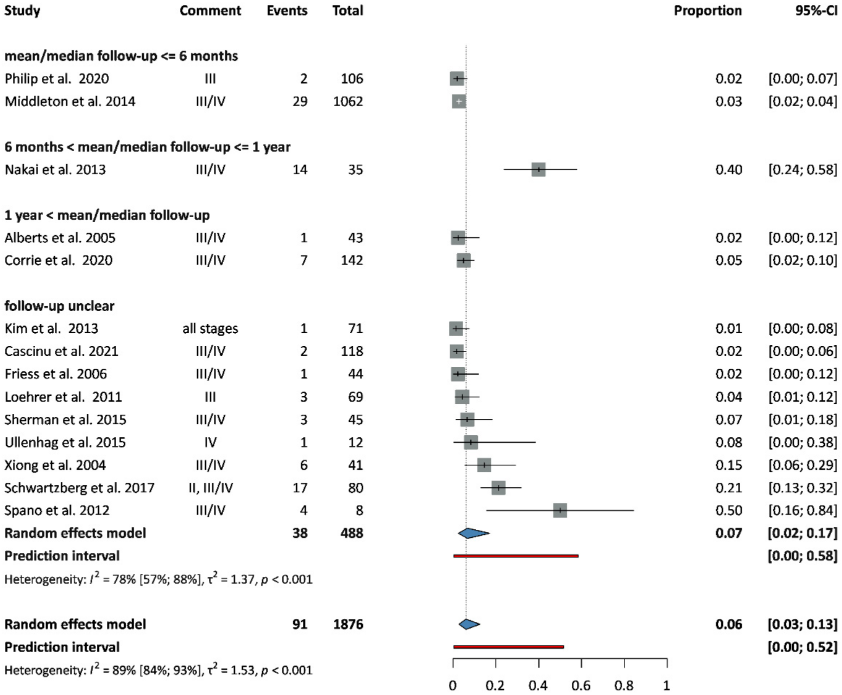

Figure S8. Incidence of thrombotic events with different follow-up times
CI: confidence interval

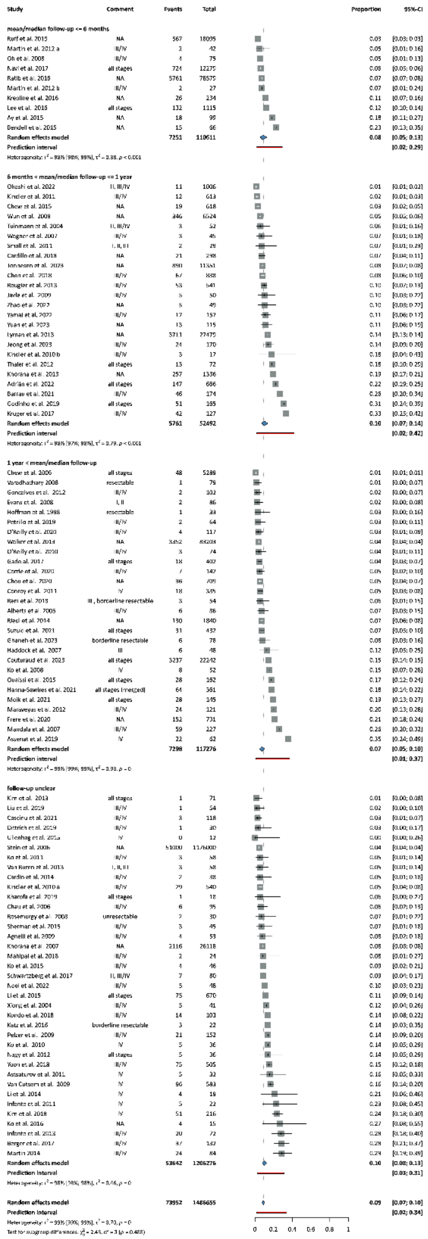

Figure S9. Incidence of thrombotic events among patients with TNM III-IV.

CI: confidence interval

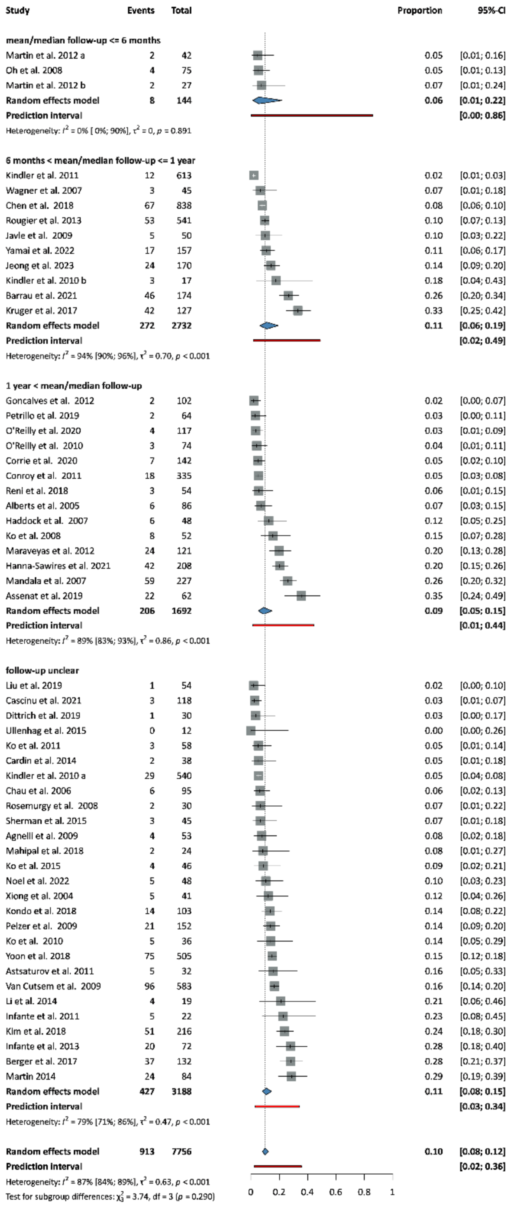

Figure S10. Incidence of thrombotic events among patients **A:** who received Gemcitabine-based therapy **B:** with TNM III-IV who received Gemcitabine-based therapy. CI: confidence interval.

A:

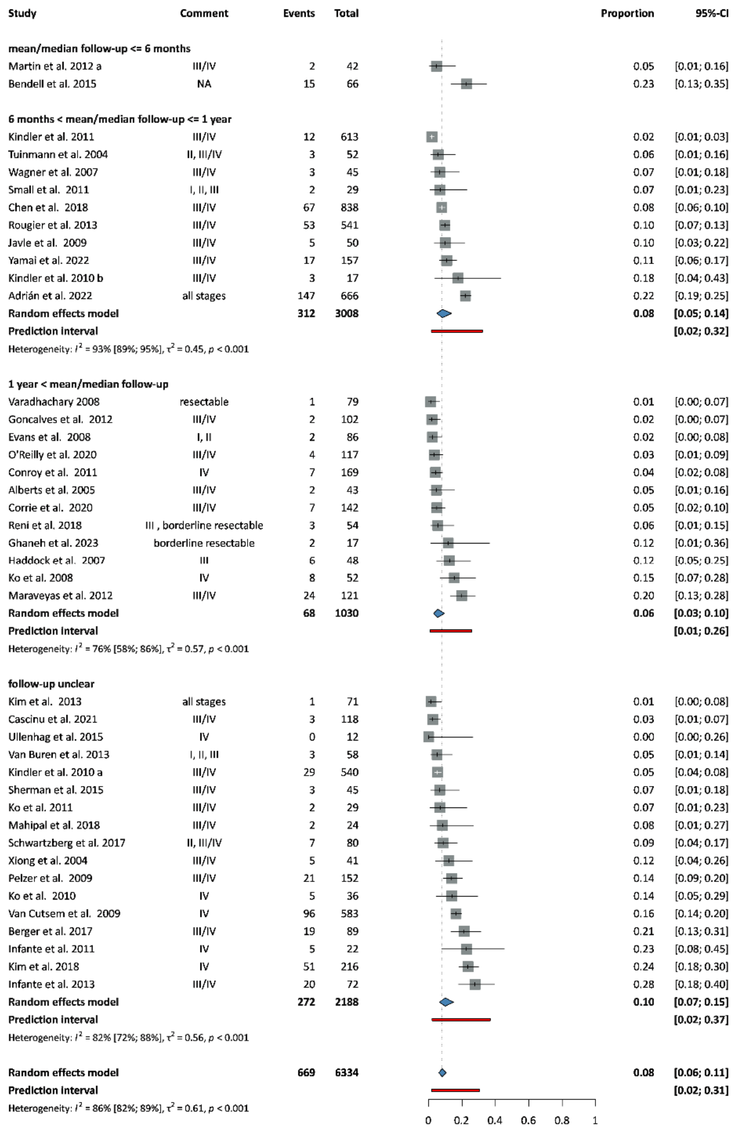

B:

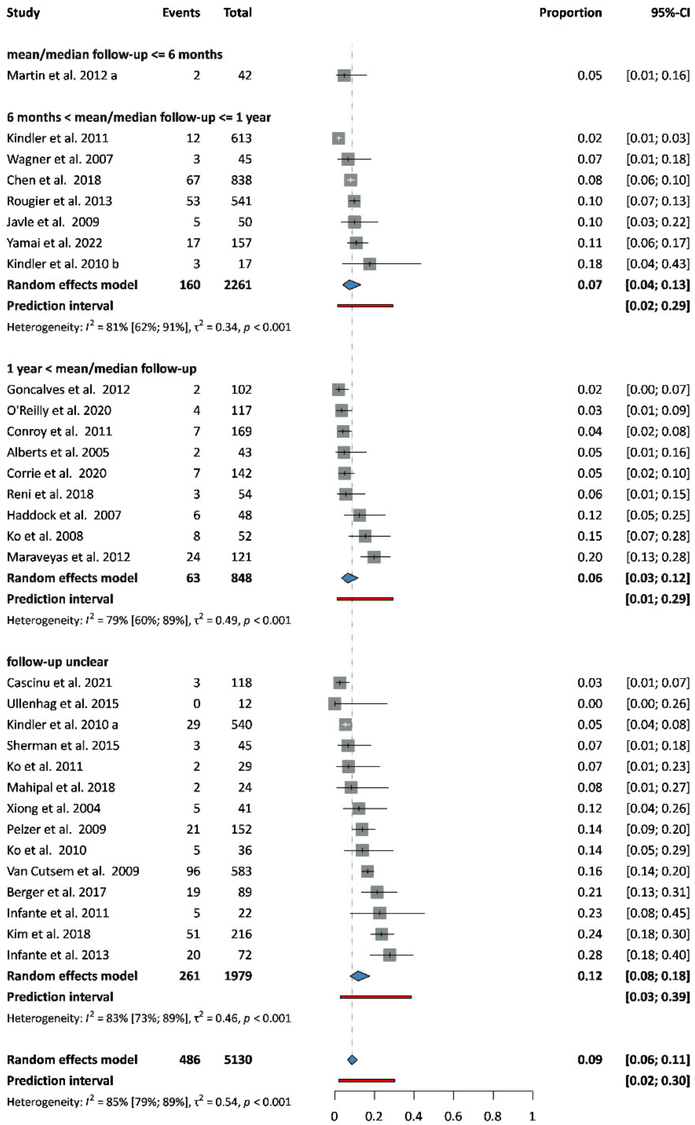

Figure S11. Incidence of thrombotic events among patients **A:** who received treatment in association with monoclonal antibodies **B:** with TNM III-IV who received treatment in association with monoclonal antibodies. CI: confidence interval.

A:

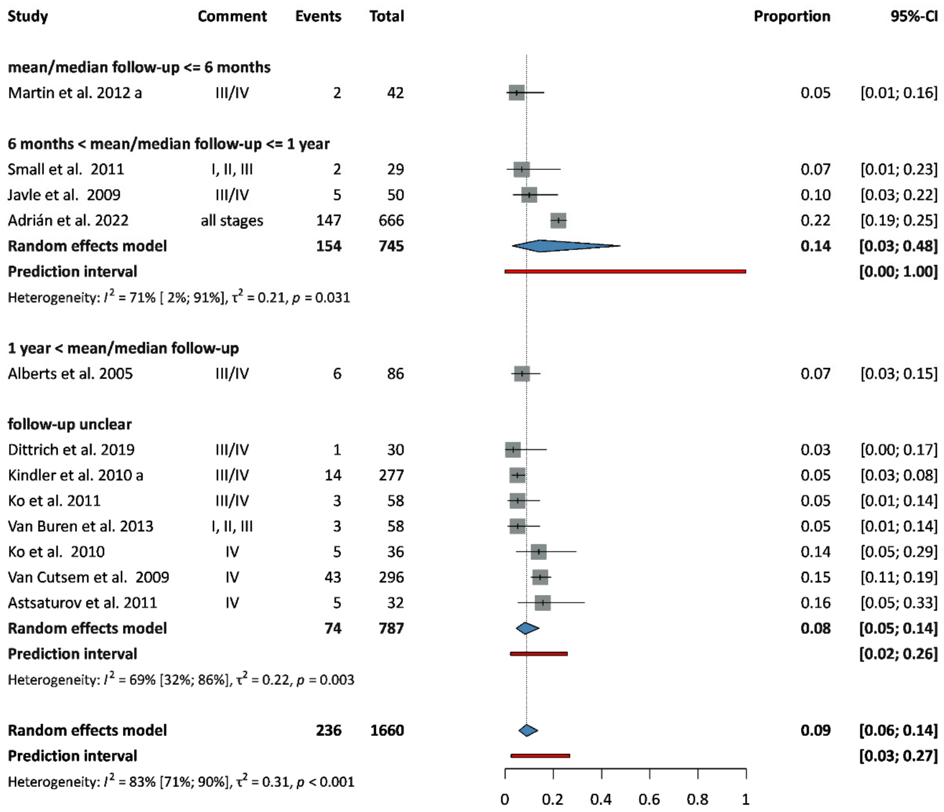

B:

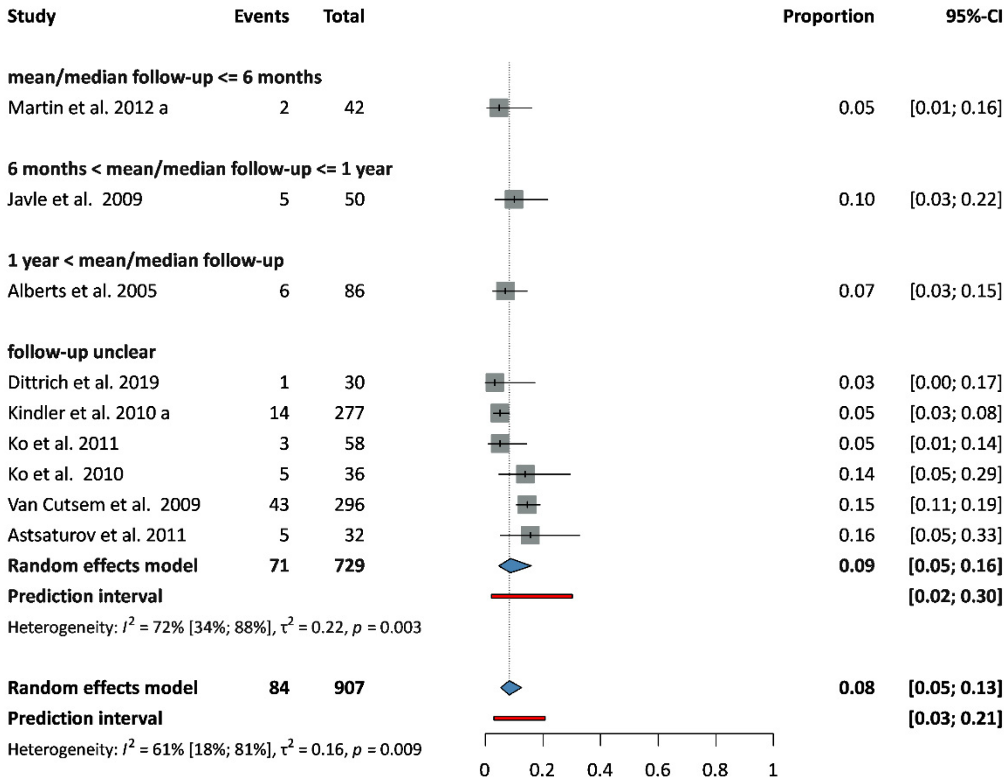

Figure S12. Incidence of thrombotic events among patients who received treatment in association with small-molecule inhibitors. CI: confidence interval.

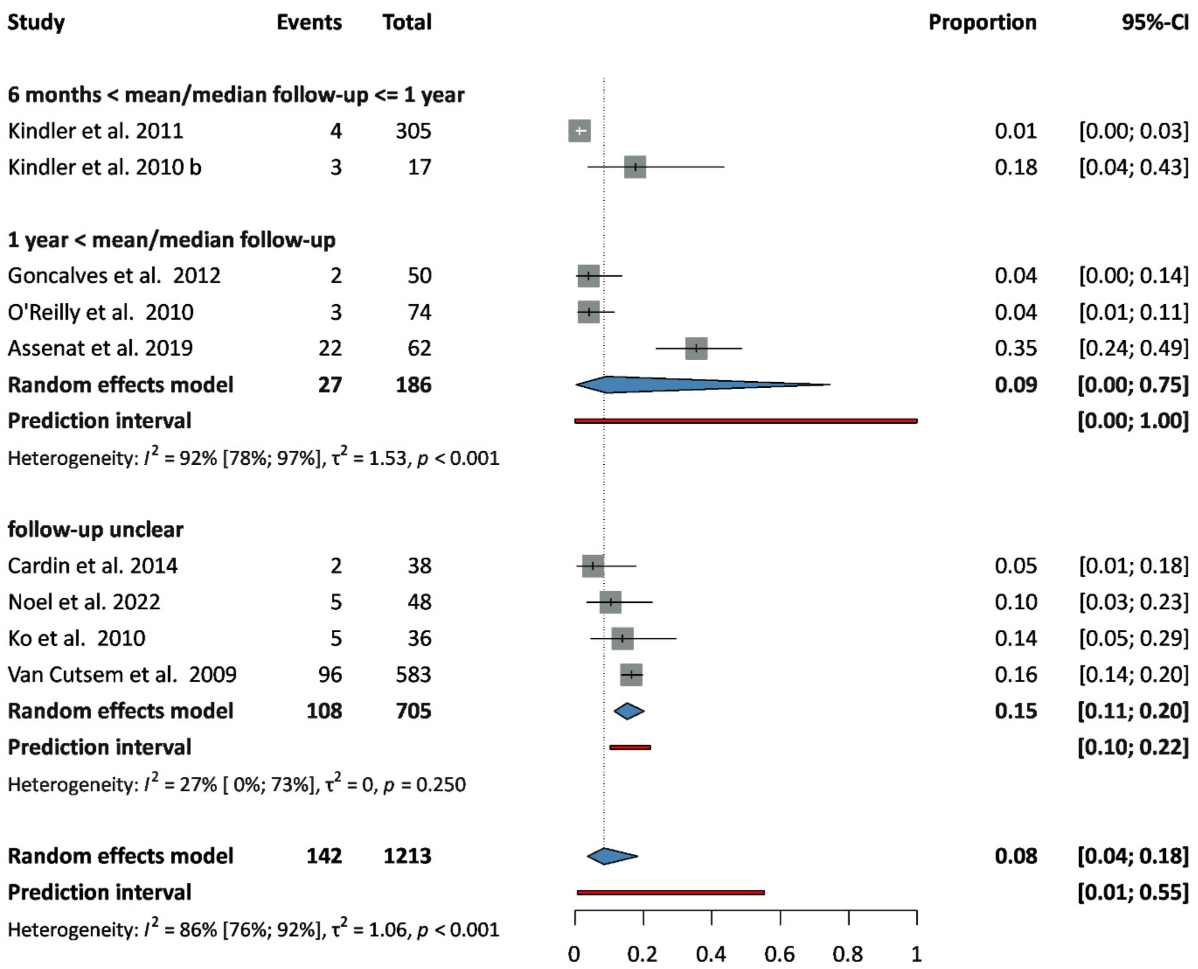

Figure S13. Incidence of pulmonary embolism among patients **A:** with TNM III-IV. **B:** who received Gemcitabine-based therapy **C:** with TNM III-IV. who received treatment in association with small molecule inhibitors. CI: confidence interval.

A:

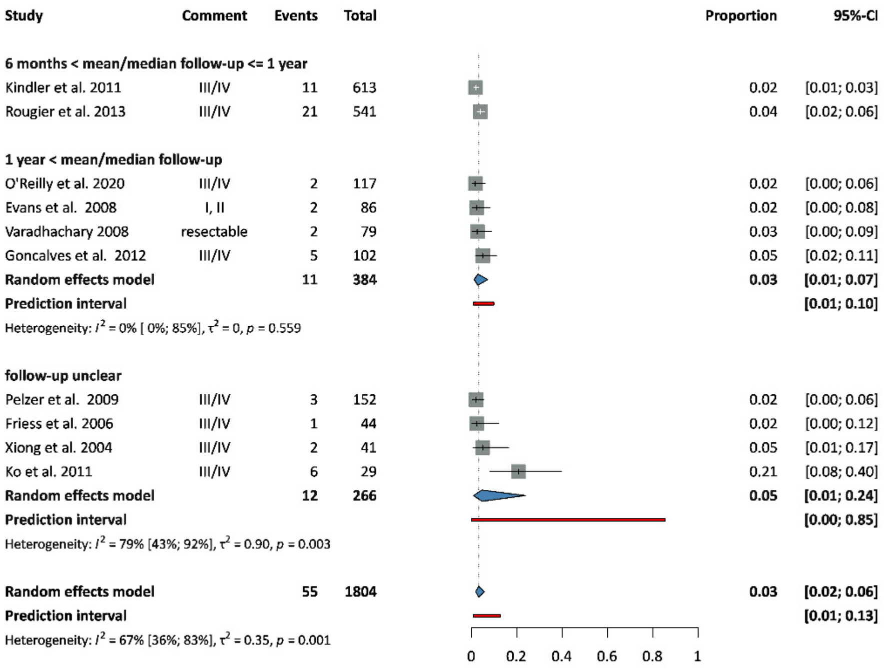

B:

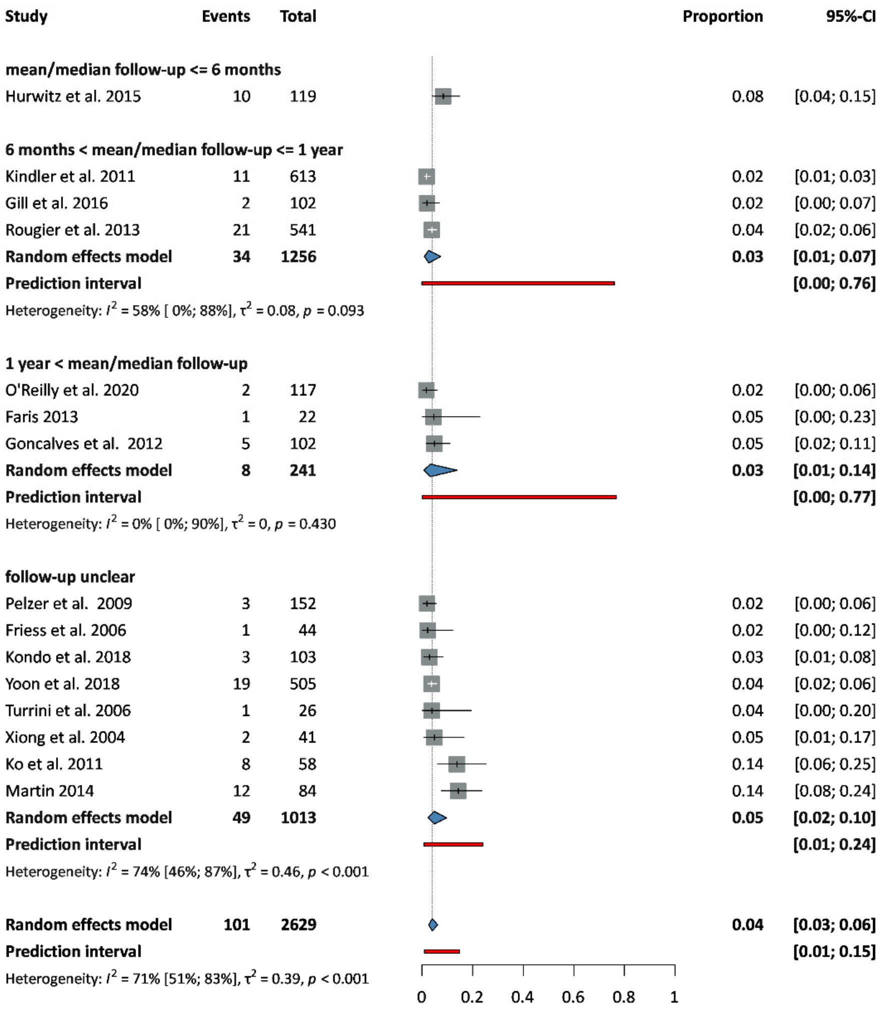

C:

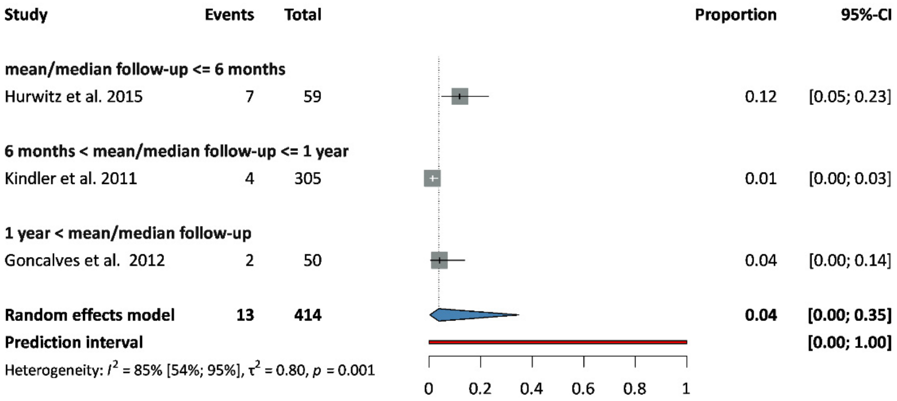

Figure S14. Incidence of hypertension A: including observational studies B: including randomised controlled trials. CI: confidence interval.

A:

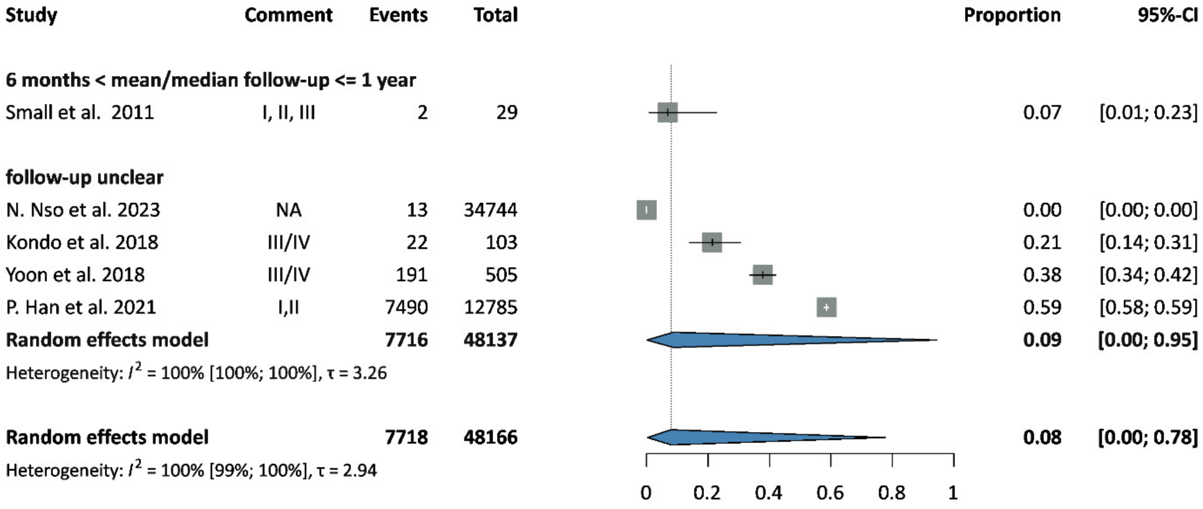

B:

Study	Comment	Events	Total	Proportion	95%-CI
**mean/median follow-up <= 6 months**					
P.A.Philip et al. 2020	III	2	106	0.02	[0.00; 0.07]
J.P.Spano et al. 2008	III/IV	15	99	0.15	[0.09; 0.24]
Martin et al. 2012 a	III/IV	8	42	0.19	[0.09; 0.34]
Pant et al. 2016	III/IV	34	163	0.21	[0.15; 0.28]
**Random effects model**		**59**	**410**	**0.12**	**[0.03; 0.37]**
Heterogeneity: I^2 = 77% [37%; 92%], τ = 0.86					
**6 months < mean/median follow-up <= 1 year**					
Kindler et al. 2010 b	III/IV	1	17	0.06	[0.00; 0.29]
Kindler et al. 2011	III/IV	112	613	0.18	[0.15; 0.22]
Rougier et al. 2013	III/IV	111	541	0.21	[0.17; 0.24]
Javle et al. 2009	III/IV	16	50	0.32	[0.20; 0.47]
**Random effects model**		**240**	**1221**	**0.20**	**[0.16; 0.24]**
Heterogeneity: I^2 = 59% [0%; 86%], τ = 0					
**1 year < mean/median follow-up**					
Goncalves et al. 2012	III/IV	4	102	0.04	[0.01; 0.10]
OReilly et al. 2010	III/IV	3	74	0.04	[0.01; 0.11]
Ghaneh et al. 2023	borderline resectable	6	78	0.08	[0.03; 0.16]
J. Aparicio et al. 2011	III/IV	1	12	0.08	[0.00; 0.38]
Reiss et al. 2022	III/IV	10	91	0.11	[0.05; 0.19]
Ko et al. 2008	IV	7	52	0.13	[0.06; 0.26]
Fogelman et al. 2011	III/IV	18	47	0.38	[0.25; 0.54]
**Random effects model**		**49**	**456**	**0.10**	**[0.04; 0.21]**
Heterogeneity: I^2 = 83% [67%; 92%], τ = 0.83					
**follow-up unclear**					
Cascinu et al. 2021	III/IV	1	118	0.01	[0.00; 0.05]
Van Buren et al. 2013	I, II, III	1	58	0.02	[0.00; 0.09]
Kindler et al. 2010 a	III/IV	13	540	0.02	[0.01; 0.04]
Rivera et al. 2019	III/IV	2	40	0.05	[0.01; 0.17]
M. M. Mita et al. 2016	III/IV	1	16	0.06	[0.00; 0.30]
Cardin et al. 2014	III/IV	3	38	0.08	[0.02; 0.21]
Mahipal et al. 2018	III/IV	2	24	0.08	[0.01; 0.27]
M.Reni et al. 2013	IV	3	27	0.11	[0.02; 0.29]
V.J. Picozzi et al. 2015	III/IV	7	58	0.12	[0.05; 0.23]
Xiong et al. 2004	III/IV	5	41	0.12	[0.04; 0.26]
Astsaturov et al. 2011	IV	4	32	0.12	[0.04; 0.29]
Van Cutsem et al. 2009	IV	86	583	0.15	[0.12; 0.18]
Ko et al. 2015	III/IV	8	46	0.17	[0.08; 0.31]
D. B. Zhen et al. 2016	III/IV	4	11	0.36	[0.11; 0.69]
**Random effects model**		**140**	**1632**	**0.08**	**[0.04; 0.13]**
**Prediction interval**					**[0.01; 0.35]**
Heterogeneity: I^2 = 79% [66%; 87%], τ = 0.83					
**Random effects model**		**488**	**3719**	**0.10**	**[0.07; 0.14]**
**Prediction interval**					**[0.02; 0.41]**

Heterogeneity: I^2 = 82% [74%; 87%], τ = 0.88
Test for subgroup differences: χ^2_3 = 20.31, df = 3 (p < 0.001)

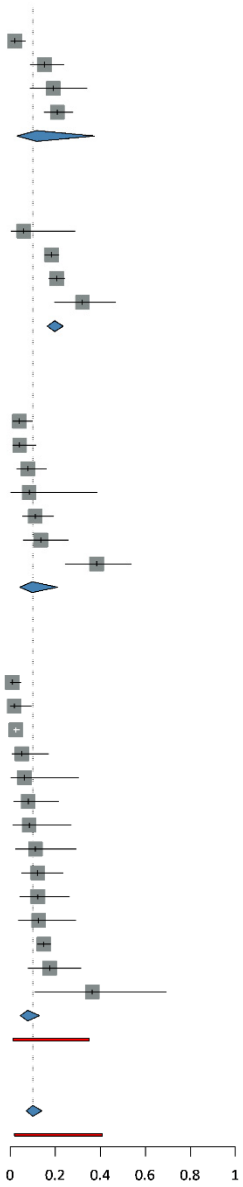

Figure S15. Incidence of pulmonary embolism A: including observational studies B: including randomised controlled trials. CI: confidence interval.

A:

Study	Comment	Events	Total	Proportion	95%-CI
**mean/median follow-up <= 6 months**					
H.I. Hurwitz et al. 2015	IV	10	119	0.08	[0.04; 0.15]
**6 months < mean/median follow-up <= 1 year**					
Kindler et al. 2011	III/IV	11	613	0.02	[0.01; 0.03]
Gill et al. 2016	III/IV	2	102	0.02	[0.00; 0.07]
Rougier et al. 2013	III/IV	21	541	0.04	[0.02; 0.06]
**Random effects model**		**34**	**1256**	**0.03**	**[0.01; 0.07]**
Heterogeneity: I^2 = 58% [0%; 88%], τ = 0.27					
**1 year < mean/median follow-up**					
OReilly et al. 2020	III/IV	2	117	0.02	[0.00; 0.06]
Evans et al. 2008	I, II	2	86	0.02	[0.00; 0.08]
Varadhachary 2008	resectable	2	79	0.03	[0.00; 0.09]
Goncalves et al. 2012	III/IV	5	102	0.05	[0.02; 0.11]
**Random effects model**		**11**	**384**	**0.03**	**[0.01; 0.07]**
Heterogeneity: I^2 = 0% [0%; 85%], τ = 0					
**follow-up unclear**					
Pelzer et al. 2009	III/IV	3	152	0.02	[0.00; 0.06]
Friess et al. 2006	III/IV	1	44	0.02	[0.00; 0.12]
Xiong et al. 2004	III/IV	2	41	0.05	[0.01; 0.17]
Ko et al. 2011	III/IV	8	58	0.14	[0.06; 0.25]
**Random effects model**		**14**	**295**	**0.04**	**[0.01; 0.18]**
Heterogeneity: I^2 = 72% [20%; 90%], τ = 0.75					
**Random effects model**		**69**	**2054**	**0.03**	**[0.02; 0.05]**
**Prediction interval**					**[0.01; 0.12]**
Heterogeneity: I^2 = 65% [35%; 81%], τ = 0.56					

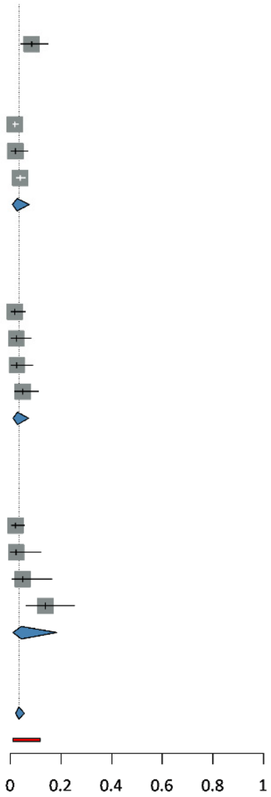

B:

Study	Comment	Events	Total	Proportion	95%-CI
**6 months < mean/median follow-up <= 1 year**					
Godinho et al. 2019	all stages	12	165	0.07	[0.04; 0.12]
**1 year < mean/median follow-up**					
J.E. Faris 2013	III	1	22	0.05	[0.00; 0.23]
Couturaud et al. 2023	all stages	2315	22242	0.10	[0.10; 0.11]
**follow-up unclear**					
Kondo et al. 2018	III/IV	3	103	0.03	[0.01; 0.08]
Yoon et al. 2018	III/IV	19	505	0.04	[0.02; 0.06]
Turrini et al. 2006	III	1	26	0.04	[0.00; 0.20]
Li et al. 2015	all stages	29	670	0.04	[0.03; 0.06]
Martin 2014	III/IV	12	84	0.14	[0.08; 0.24]
**Random effects model**		**64**	**1388**	**0.05**	**[0.02; 0.10]**
Heterogeneity: I^2 = 76% [42%; 90%], τ = 0.51					
**Random effects model**		**2392**	**23817**	**0.06**	**[0.04; 0.10]**
**Prediction interval**					**[0.02; 0.19]**
Heterogeneity: I^2 = 87% [77%; 93%], τ = 0.49					

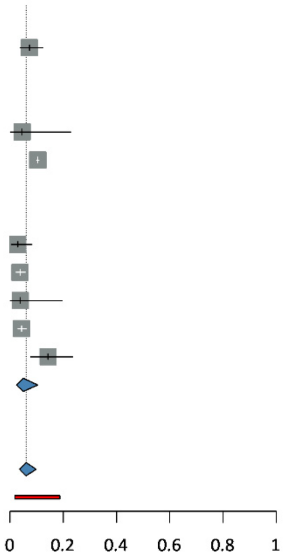

Figure S16. Incidence of thrombotic events A: including observational studies B: including randomised controlled trials. CI: confidence interval.

A:

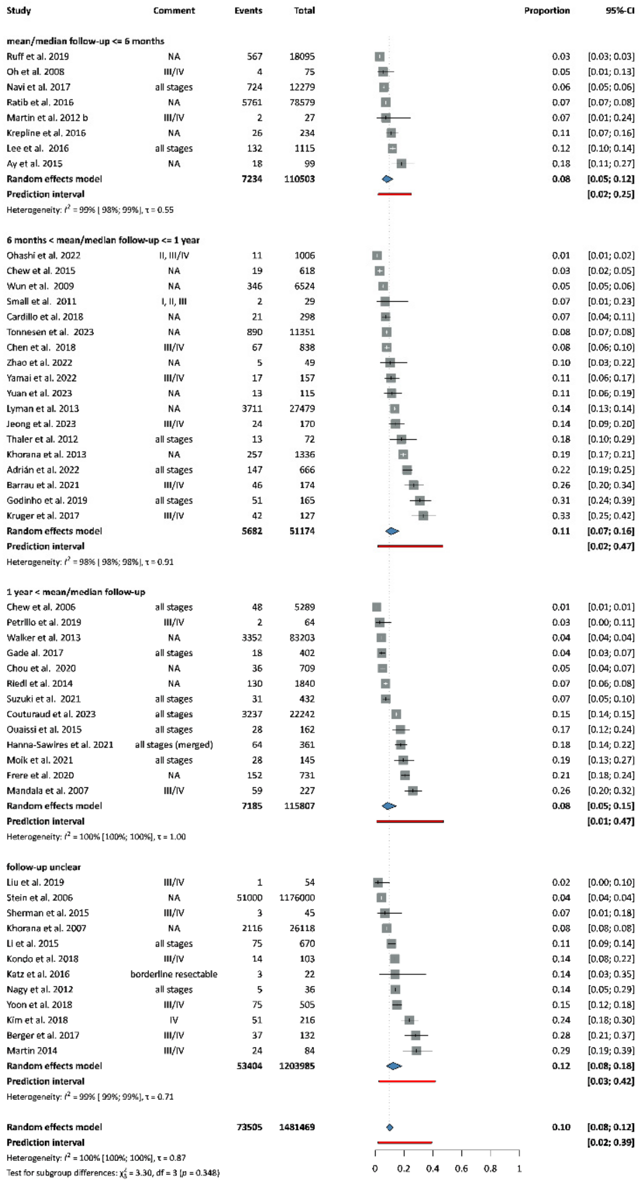

B:

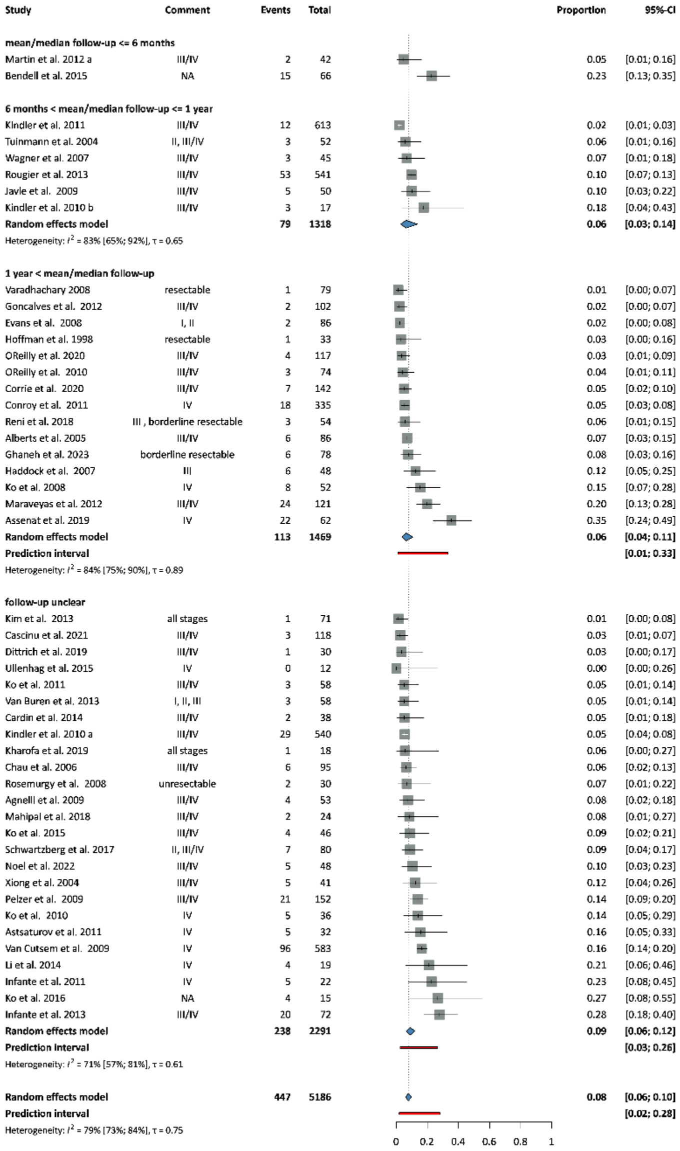

Figure S17. Funnel plot for the analysis of the incidence of thrombotic events.

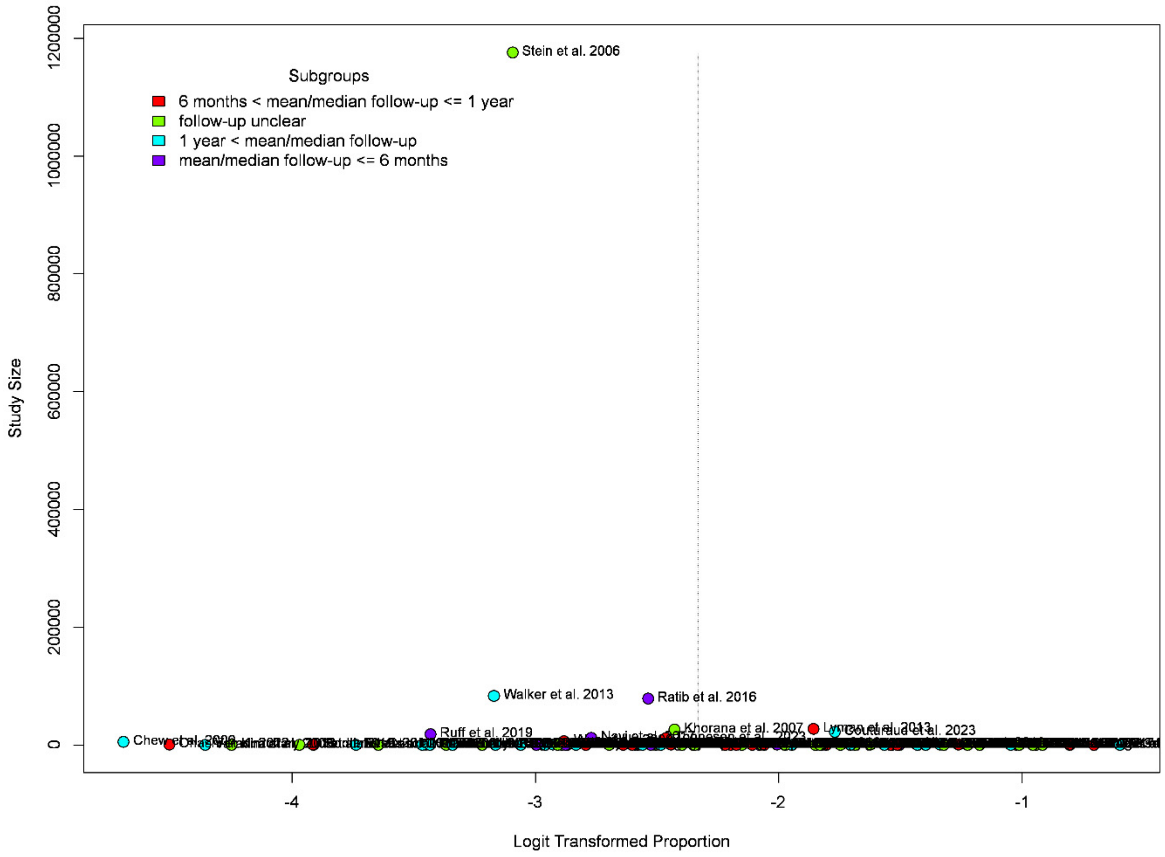

References

1. Navi BB, Reiner AS, Kamel H, Iadecola C, Okin PM, Elkind MSV, et al. Risk of Arterial Thromboembolism in Patients With Cancer. J Am Coll Cardiol. 2017 Aug;70(8):926–38.

2. D Baluka et al. The role of the tissue plasminogen activator as a prognostic and differentiation factor in patients with pancreatic cancer and chronic pancreatitis. J Physiol Pharmacol. 2016;93–101.

3. Huang BZ, Liu L, Zhang J, Pandol SJ, Grossman SR, Setiawan VW, et al. Rising Incidence and Racial Disparities of Early-Onset Pancreatic Cancer in the United States, 1995–2018. Gastroenterology. 2022 Jul;163(1):310-312.e1.

4. Lee JC, Ro YS, Cho J, Park Y, Lee JH, Hwang JH, et al. Characteristics of Venous Thromboembolism in Pancreatic Adenocarcinoma in East Asian Ethnics. Medicine. 2016 Apr;95(17):e3472.

5. Ryan JF, Rosati LM, Groot VP, Le DT, Zheng L, Laheru DA, et al. Stereotactic body radiation therapy for palliative management of pancreatic adenocarcinoma in elderly and medically inoperable patients. Oncotarget. 2018 Mar 27;9(23):16427–36.

6. Shen J, Pan P, Hu X, Zhao J, Wu H. Safety and Efficacy of Irreversible Electroporation in Locally Advanced Pancreatic Cancer: An Evaluation from a Surgeon's Perspective. Cancers (Basel). 2022 Nov 18;14(22):5677.

7. Tønnesen J, Pallisgaard J, Rasmussen PV, Ruwald MH, Lamberts M, Nouhravesh N, et al. Risk and timing of venous thromboembolism in patients with gastrointestinal cancer: a nationwide Danish cohort study. BMJ Open. 2023 Jan 19;13(1):e062768.

8. Søgaard KK, Farkas DK, Pedersen L, Sørensen HT. Splanchnic venous thrombosis is a marker of cancer and a prognostic factor for cancer survival. Blood. 2015 Aug 20;126(8):957–63.

9. Anker MS, Ebner N, Hildebrandt B, Springer J, Sinn M, Riess H, et al. Resting heart rate is an independent predictor of death in patients with colorectal, pancreatic, and non-small cell lung cancer: results of a prospective cardiovascular long-term study. Eur J Heart Fail. 2016 Dec 2;18(12):1524–34.

10. Ratib S, Walker AJ, Card TR, Grainge MJ. Risk of venous thromboembolism in hospitalised cancer patients in England—a cohort study. J Hematol Oncol. 2016 Dec 26;9(1):60.

11. Gonçalves A, Gilabert M, François E, Dahan L, Perrier H, Lamy R, et al. BAYPAN study: a double-blind phase III randomized trial comparing gemcitabine plus sorafenib and gemcitabine plus placebo in patients with advanced pancreatic cancer. Annals of Oncology. 2012 Nov;23(11):2799–805.

12. Le Bozec A, Brugel M, Djerada Z, Ayad M, Perrier M, Carlier C, et al. Beta-blocker exposure and survival outcomes in patients with advanced pancreatic ductal adenocarcinoma: a retrospective cohort study (BETAPANC). Front Pharmacol. 2023 May 19;14.

13. Navi BB, Reiner AS, Kamel H, Iadecola C, Elkind MS V., Panageas KS, et al. Association between incident cancer and subsequent stroke. Ann Neurol. 2015 Feb 7;77(2):291–300.

14. Navi BB, Reiner AS, Kamel H, Iadecola C, Okin PM, Tagawa ST, et al. Arterial thromboembolic events preceding the diagnosis of cancer in older persons. Blood. 2019 Feb 21;133(8):781–9.

15. Faille D, Bourrienne MC, de Raucourt E, de Chaisemartin L, Granger V, Lacroix R, et al. Biomarkers for the risk of thrombosis in pancreatic adenocarcinoma are related to cancer process. Oncotarget. 2018 May 29;9(41):26453–65.

16. Fogelman D, Jafari M, Varadhachary GR, Xiong H, Bullock S, Ozer H, et al. Bevacizumab plus gemcitabine and oxaliplatin as first-line therapy for metastatic or locally advanced pancreatic cancer: a phase II trial. Cancer Chemother Pharmacol. 2011 Dec 9;68(6):1431–8.

17. Li D, Pise MN, Overman MJ, Liu C, Tang H, Vadhan-Raj S, et al. <scp>ABO</scp> non-O type as a risk factor for thrombosis in patients with pancreatic cancer. Cancer Med. 2015 Nov 15;4(11):1651–8.

18. Yip D, Goldstein D. Adding irinotecan to first-line gemcitabine improves tumour response in advanced pancreatic cancer. Cancer Treat Rev. 2005 May;31(3):236–41.

19. Couturaud F, Mahé I, Schmidt J, Gleize JC, Lafon T, Saighi A, et al. Adult breast, lung, pancreatic, upper and lower gastrointestinal cancer patients with hospitalized venous thromboembolism in the national French hospital discharge database. BMC Cancer. 2023 Jun 10;23(1):531.

20. Stitzel HJ, Hue JJ, Elshami M, McCaulley L, Hoehn RS, Rothermel LD, et al. Assessing the use of Extended Venous Thromboembolism Prophylaxis on the Rates of Venous Thromboembolism and Postpancreatectomy Hemorrhage Following Pancreatectomy for Malignancy. Ann Surg. 2023 Jul;278(1):e80–6.

21. Kindler HL, Ioka T, Richel DJ, Bennouna J, Létourneau R, Okusaka T, et al. Axitinib plus gemcitabine versus placebo plus gemcitabine in patients with advanced pancreatic adenocarcinoma: a double-blind randomised phase 3 study. Lancet Oncol. 2011 Mar;12(3):256–62.

22. Lee HS, Lee SH, Lee HJ, Chung MJ, Park JY, Park SW, et al. Statin Use and Its Impact on Survival in Pancreatic Cancer Patients. Medicine. 2016 May;95(19):e3607.

23. Javle M, Yu J, Garrett C, Pande A, Kuvshinoff B, Litwin A, et al. Bevacizumab combined with gemcitabine and capecitabine for advanced pancreatic cancer: a phase II study. Br J Cancer. 2009 Jun 2;100(12):1842–5.

24. Jiang QL, Wang CF, Tian YT, Huang H, Zhang SS, Zhao DB, et al. Body mass index does not affect the survival of pancreatic cancer patients. World J Gastroenterol. 2017;23(34):6287.

25. Cai Q, Mao Y, Yang Q, Wen H, Lv Y, Zhang R. Are left ventricular muscle area and radiation attenuation associated with overall survival in advanced pancreatic cancer patients treated with chemotherapy? Clin Radiol. 2020 Mar;75(3):238.e1-238.e9.

26. Tingle SJ, Severs GR, Moir JAG, White SA. Calcium channel blockers in pancreatic cancer: increased overall survival in a retrospective cohort study. Anticancer Drugs. 2020 Aug 3;31(7):737–41.

27. Corrie PG, Qian W, Basu B, Valle JW, Falk S, Iwuji C, et al. Scheduling nab-paclitaxel combined with gemcitabine as first-line treatment for metastatic pancreatic adenocarcinoma. Br J Cancer. 2020 Jun 9;122(12):1760–8.

28. Rosemurgy A, Luzardo G, Cooper J, Bowers C, Zervos E, Bloomston M, et al. 32P as an Adjunct to Standard Therapy for Locally Advanced Unresectable Pancreatic Cancer: A Randomized Trial. Journal of Gastrointestinal Surgery. 2008 Apr;12(4):682–8.

29. Menapace LA, Peterson DR, Berry A, Sousou T, Khorana AA. Symptomatic and incidental thromboembolism are both associated with mortality in pancreatic cancer. Thromb Haemost. 2011 Nov 25;106(08):371–8.

30. Huang YM, Shih HJ, Chen YC, Hsieh TY, Ou CW, Su PH, et al. Systemic Anticoagulation and Inpatient Outcomes of Pancreatic Cancer: Real-World Evidence from U.S. Nationwide Inpatient Sample. Cancers (Basel). 2023 Mar 26;15(7):1985.

31. Ishigaki K, Nakai Y, Isayama H, Saito K, Hamada T, Takahara N, et al. Thromboembolisms in Advanced Pancreatic Cancer. Pancreas. 2017 Sep;46(8):1069–75.

32. Pant S, Martin LK, Geyer S, Wei L, Van Loon K, Sommovilla N, et al. Treatment-related Hypertension as a Pharmacodynamic Biomarker for the Efficacy of Bevacizumab in Advanced Pancreas Cancer. Am J Clin Oncol. 2016 Dec;39(6):614–8.

33. Zhai S, Huo Z, Wang Y, Qian H, Zhao S, Shi Y, et al. TRIANGLE operation for borderline resectable pancreatic cancer in total pancreatectomy. Transl Cancer Res. 2019 Oct;8(6):2416–24.

34. Rivera F, Benavides M, Gallego J, Guillen-Ponce C, Lopez-Martin J, Küng M. Tumor treating fields in combination with gemcitabine or gemcitabine plus nab-paclitaxel in pancreatic cancer: Results of the PANOVA phase 2 study. Pancreatology. 2019 Jan;19(1):64–72.

35. Sgroi MD, Narayan RR, Lane JS, Demirjian A, Kabutey NK, Fujitani RM, et al. Vascular reconstruction plays an important role in the treatment of pancreatic adenocarcinoma. J Vasc Surg. 2015 Feb;61(2):475–80.

36. Martin RCG, McFarland K, Ellis S, Velanovich V. Irreversible Electroporation Therapy in the Management of Locally Advanced Pancreatic Adenocarcinoma. J Am Coll Surg. 2012 Sep;215(3):361–9.

37. Wun T, White RH. Venous Thromboembolism (VTE) in Patients with Cancer: Epidemiology and Risk Factors. Cancer Invest. 2009 Jan 11;27(sup1):63–74.

38. Chen JS, Hung CY, Chang H, Liu CT, Chen YY, Lu CH, et al. Venous Thromboembolism in Asian Patients with Pancreatic Cancer Following Palliative Chemotherapy: Low Incidence but a Negative Prognosticator for Those with Early Onset. Cancers (Basel). 2018 Dec 10;10(12):501.

39. Suzuki T, Hori R, Takeuchi K, Yamamura R, Katoh H, Noji Y, et al. Venous Thromboembolism in Japanese Patients With Pancreatic Cancer. Clinical and Applied Thrombosis/Hemostasis. 2021 Jan 3;27.

40. Laderman L, Sreekrishnanilayam K, Pandey RK, Handorf E, Blumenreich A, Sorice KA, et al. Venous thromboembolism in metastatic pancreatic cancer. Eur J Haematol. 2023 Jun 30;110(6):706–14.

41. Jeong HT, Bae JH, Kim HG, Han J. Venous Thromboembolism in Patients with Advanced Pancreatic Cancer Receiving Palliative Chemotherapy: Incidence and Effect on Prognosis. The Korean Journal of Gastroenterology. 2023 Mar 25;81(3):109–20.

42. Ruff SM, Weber KT, Khader A, Conte C, Kadison A, Sullivan J, et al. Venous thromboembolism in patients with cancer undergoing surgical exploration. J Thromb Thrombolysis. 2019 Feb 17;47(2):316–23.

43. Oh SY, Kim JH, Lee KW, Bang SM, Hwang JH, Oh D, et al. Venous thromboembolism in patients with pancreatic adenocarcinoma: Lower incidence in Asian ethnicity. Thromb Res. 2008 Jan;122(4):485–90.

44. Mandalà M, Reni M, Cascinu S, Barni S, Floriani I, Cereda S, et al. Venous thromboembolism predicts poor prognosis in irresectable pancreatic cancer patients. Annals of Oncology. 2007 Oct;18(10):1660–5.

45. Krepline AN, Christians KK, George B, Ritch PS, Erickson BA, Tolat P, et al. Venous thromboembolism prophylaxis during neoadjuvant therapy for resectable and borderline resectable pancreatic cancer-Is it indicated? J Surg Oncol. 2016 Oct;114(5):581–6.

46. Lyman GH, Eckert L, Wang Y, Wang H, Cohen A. Venous Thromboembolism Risk in Patients With Cancer Receiving Chemotherapy: A Real-World Analysis. Oncologist. 2013 Dec 1;18(12):1321–9.

47. Kim SS, Nakakura EK, Wang ZJ, Kim GE, Corvera CU, Harris HW, et al. Preoperative FOLFIRINOX for borderline resectable pancreatic cancer: Is radiation necessary in the modern era of chemotherapy? J Surg Oncol. 2016 Oct;114(5):587–96.

48. Rich T, Winter K, Safran, Hoffman, Erickson B, Anne R, et al. Weekly paclitaxel, gemcitabine, and external irradiation followed by randomized farnesyl transferase inhibitor R115777 for locally advanced pancreatic cancer. Onco Targets Ther. 2012 Aug;161.

49. Katz MHG, Ou FS, Herman JM, Ahmad SA, Wolpin B, Marsh R, et al. Alliance for clinical trials in oncology (ALLIANCE) trial A021501: preoperative extended chemotherapy vs. chemotherapy plus hypofractionated radiation therapy for borderline resectable adenocarcinoma of the head of the pancreas. BMC Cancer. 2017 Dec 27;17(1):505.

50. Zhao M, Wang DDH, Liu X, Tian R. Metabolic Modulation of Macrophage Function Post Myocardial Infarction. Front Physiol. 2020 Jun 30;11.

51. Alberts SR, Foster NR, Morton RF, Kugler J, Schaefer P, Wiesenfeld M, et al. PS-341 and gemcitabine in patients with metastatic pancreatic adenocarcinoma: a North Central Cancer Treatment Group (NCCTG) randomized phase II study. Annals of Oncology. 2005 Oct;16(10):1654–61.

52. O'Reilly EM, Barone D, Mahalingam D, Bekaii-Saab T, Shao SH, Wolf J, et al. Randomised phase II trial of gemcitabine and nab-paclitaxel with necuparanib or placebo in untreated metastatic pancreas ductal adenocarcinoma. Eur J Cancer. 2020 Jun;132:112–21.

53. Infante JR, Somer BG, Park JO, Li CP, Scheulen ME, Kasubhai SM, et al. A randomised, double-blind, placebo-controlled trial of trametinib, an oral MEK inhibitor, in combination with gemcitabine for patients with untreated metastatic adenocarcinoma of the pancreas. Eur J Cancer. 2014 Aug;50(12):2072–81.

54. Rougier P, Riess H, Manges R, Karasek P, Humblet Y, Barone C, et al. Randomised, placebo-controlled, double-blind, parallel-group phase III study evaluating aflibercept in patients receiving first-line treatment with gemcitabine for metastatic pancreatic cancer. Eur J Cancer. 2013 Aug;49(12):2633–42.

55. Friess H, Langrehr JM, Oettle H, Raedle J, Niedergethmann M, Dittrich C, et al. A randomized multi-center phase II trial of the angiogenesis inhibitor Cilengitide (EMD 121974) and gemcitabine compared with gemcitabine alone in advanced unresectable pancreatic cancer. BMC Cancer. 2006 Dec 11;6(1):285.

56. El-Khoueiry AB, Ramanathan RK, Yang DY, Zhang W, Shibata S, Wright JJ, et al. A randomized phase II of gemcitabine and sorafenib versus sorafenib alone in patients with metastatic pancreatic cancer. Invest New Drugs. 2012 Jun 22;30(3):1175–83.

57. Noel MS, Kim S, Hartley ML, Wong S, Picozzi VJ, Staszewski H, et al. A randomized phase <scp>II</scp> study of <scp>SM</scp> -88 plus methoxsalen, phenytoin, and sirolimus in patients with metastatic pancreatic cancer treated in the second line and beyond. Cancer Med. 2022 Nov 2;11(22):4169–81.

58. Burtness B, Powell M, Catalano P, Berlin J, Liles DK, Chapman AE, et al. Randomized Phase II Trial of Irinotecan/Docetaxel or Irinotecan/Docetaxel Plus Cetuximab for Metastatic Pancreatic Cancer. Am J Clin Oncol. 2016 Aug;39(4):340–5.

59. He D, Qin K, Li J, Li Y, Chen Z, Xu J, et al. Increased incidence risks of cardiovascular disease among cancer patients: Evidence from a population-based cohort study in China. Int J Cardiol. 2024 Feb;396:131362.

60. Nakai Y, Isayama H, Ijichi H, Sasaki T, Sasahira N, Hirano K, et al. Inhibition of renin–angiotensin system affects prognosis of advanced pancreatic cancer receiving gemcitabine. Br J Cancer. 2010 Nov 26;103(11):1644–8.

61. Kamarajah SK, Chatzizacharias N, Hodson J, Marcon F, Kalisvaart M, Punia P, et al. Intention to treat outcomes among patients with pancreatic cancer treated using International Study Group on Pancreatic Surgery recommended pathways for resectable and borderline resectable disease. ANZ J Surg. 2021 Jul 12;91(7–8):1549–57.

62. Rocha FG, Hashimoto Y, Traverso LW, Dorer R, Kozarek R, Helton WS, et al. Interferon-based Adjuvant Chemoradiation for Resected Pancreatic Head Cancer. Ann Surg. 2016 Feb;263(2):376–84.

63. Kambakamba P, Bonvini JM, Glenck M, Castrezana López L, Pfammatter T, Clavien PA, et al. Intraoperative adverse events during irreversible electroporation–a call for caution. The American Journal of Surgery. 2016 Oct;212(4):715–21.

64. Terada S, Satoh T, Endo S, Hawke P, Nakatani E, Sato Y, et al. Intratumoral Air Bubbles and Hematemesis As Predictors of Pseudoaneurysm Rupture in Unresected Pancreatic Cancer. Internal Medicine. 2023 Jul 1;62(13):0856–22.

65. Liu S, Qin Z, Xu J, Zeng J, Chen J, Niu L, et al. <p>Irreversible electroporation combined with chemotherapy for unresectable pancreatic carcinoma: a prospective cohort study</p>. Onco Targets Ther. 2019 Feb;Volume 12:1341–50.

66. Turrini O, Moutardier V, Guiramand J, Viret F, Giovaninni M, Delpero JR. Long term morbidity of neoadjuvant chemoradiation for pancreatic head adenocarcinoma. European Journal of Surgical Oncology (EJSO). 2006 Feb;32(1):77–9.

67. Yuan S, Sun Y, Chen J, Li X, Larsson SC. Long-term risk of venous thromboembolism among patients with gastrointestinal non-neoplastic and neoplastic diseases: A prospective cohort study of 484 211 individuals. Am J Hematol. 2024 Feb 27;99(2):172–81.

68. THALER J, AY C, MACKMAN N, BERTINA RM, KAIDER A, MAROSI C, et al. Microparticle-associated tissue factor activity, venous thromboembolism and mortality in pancreatic, gastric, colorectal and brain cancer patients. Journal of Thrombosis and Haemostasis. 2012 Jul;10(7):1363–70.

69. Nakai Y, Isayama H, Ijichi H, Sasaki T, Takahara N, Ito Y, et al. A multicenter phase II trial of gemcitabine and candesartan combination therapy in patients with advanced pancreatic cancer: GECA2. Invest New Drugs. 2013 Oct 1;31(5):1294–9.

70. Cascinu S, Berardi R, Bianco R, Bilancia D, Zaniboni A, Ferrari D, et al. Nab-paclitaxel/gemcitabine combination is more effective than gemcitabine alone in locally advanced, unresectable pancreatic cancer – A GISCAD phase II randomized trial. Eur J Cancer. 2021 May;148:422–9.

71. Agnelli G, Gussoni G, Bianchini C, Verso M, Mandalà M, Cavanna L, et al. Nadroparin for the prevention of thromboembolic events in ambulatory patients with metastatic or locally advanced solid cancer receiving chemotherapy: a randomised, placebo-controlled, double-blind study. Lancet Oncol. 2009 Oct;10(10):943–9.

72. Sherman WH, Chu K, Chabot J, Allendorf J, Schrope BA, Hecht E, et al. Neoadjuvant gemcitabine, docetaxel, and capecitabine followed by gemcitabine and capecitabine/radiation therapy and surgery in locally advanced, unresectable pancreatic adenocarcinoma. Cancer. 2015 Mar 9;121(5):673–80.

73. Maulat C, Canivet C, Touraine C, Gourgou S, Napoleon B, Palazzo L, et al. A New Score to Predict the Resectability of Pancreatic Adenocarcinoma: The BACAP Score. Cancers (Basel). 2020 Mar 25;12(4):783.

74. Reiss KA, Mick R, Teitelbaum U, O'Hara M, Schneider C, Massa R, et al. Niraparib plus nivolumab or niraparib plus ipilimumab in patients with platinum-sensitive advanced pancreatic cancer: a randomised, phase 1b/2 trial. Lancet Oncol. 2022 Aug;23(8):1009–20.

75. Ohashi Y, Ikeda M, Kunitoh H, Sasako M, Okusaka T, Mukai H, et al. One-year incidence of venous thromboembolism, bleeding, and death in patients with solid tumors newly initiating cancer treatment: Results from the Cancer-VTE Registry. Thromb Res. 2022 May;213:203–13.

76. Godinho J, Casa-Nova M, Moreira-Pinto J, Simões P, Paralta Branco F, Leal-Costa L, et al. ONKOTEV Score as a Predictive Tool for Thromboembolic Events in Pancreatic Cancer—A Retrospective Analysis. Oncologist. 2020 Feb 1;25(2):e284–90.

77. Ko AH, Bekaii-Saab T, Van Ziffle J, Mirzoeva OM, Joseph NM, Talasaz A, et al. A Multicenter, Open-Label Phase II Clinical Trial of Combined MEK plus EGFR Inhibition for Chemotherapy-Refractory Advanced Pancreatic Adenocarcinoma. Clinical Cancer Research. 2016 Jan 1;22(1):61–8.

78. Conti C, Pamoukdjian F, Aparicio T, Mebarki S, Poisson J, Manceau G, et al. Overall Survival and Prognostic Factors among Older Patients with Metastatic Pancreatic Cancer: A Retrospective Analysis Using a Hospital Database. Cancers (Basel). 2022 Feb 22;14(5):1105.

79. Gill S, Ko YJ, Cripps C, Beaudoin A, Dhesy-Thind S, Zulfiqar M, et al. PANCREOX: A Randomized Phase III Study of Fluorouracil/Leucovorin With or Without Oxaliplatin for Second-Line Advanced Pancreatic Cancer in Patients Who Have Received Gemcitabine-Based Chemotherapy. Journal of Clinical Oncology. 2016 Nov 10;34(32):3914–20.

80. Kharofa J, Mierzwa M, Olowokure O, Sussman J, Latif T, Gupta A, et al. Pattern of Marginal Local Failure in a Phase II Trial of Neoadjuvant Chemotherapy and Stereotactic Body Radiation Therapy for Resectable and Borderline Resectable Pancreas Cancer. Am J Clin Oncol. 2019 Mar;42(3):247–52.

81. Khorana AA, Francis CW, Culakova E, Kuderer NM, Lyman GH. Frequency, risk factors, and trends for venous thromboembolism among hospitalized cancer patients. Cancer. 2007 Nov 15;110(10):2339–46.

82. Khorana AA, Dalal M, Lin J, Connolly GC. Incidence and predictors of venous thromboembolism (VTE) among ambulatory high-risk cancer patients undergoing chemotherapy in the United States. Cancer. 2013 Feb 14;119(3):648–55.

83. Wagner AD, Buechner-Steudel P, Wein A, Schmalenberg H, Lindig U, Moehler M, et al. Gemcitabine, oxaliplatin and weekly high-dose 5-FU as 24-h infusion in chemonaive patients with advanced or metastatic pancreatic adenocarcinoma: a multicenter phase II trial of the Arbeitsgemeinschaft Internistische Onkologie (AIO). Annals of Oncology. 2007 Jan;18(1):82–7.

84. Ko AH, Dito E, Schillinger B, Venook AP, Xu Z, Bergsland EK, et al. A phase II study evaluating bevacizumab in combination with fixed-dose rate gemcitabine and low-dose cisplatin for metastatic pancreatic cancer: is an anti-VEGF strategy still applicable? Invest New Drugs. 2008 Oct 1;26(5):463–71.

85. Walker AJ, Card TR, West J, Crooks C, Grainge MJ. Incidence of venous thromboembolism in patients with cancer – A cohort study using linked United Kingdom databases. Eur J Cancer. 2013 Apr;49(6):1404–13.

86. Berger AK, Singh HM, Werft W, Muckenhuber A, Sprick MR, Trumpp A, et al. High prevalence of incidental and symptomatic venous thromboembolic events in patients with advanced pancreatic cancer under palliative chemotherapy: A retrospective cohort study. Pancreatology. 2017 Jul;17(4):629–34.

87. Mahipal A, Tella SH, Kommalapati A, Goyal G, Soares H, Neuger A, et al. Phase 1 trial of enzalutamide in combination with gemcitabine and nab-paclitaxel for the treatment of advanced pancreatic cancer. Invest New Drugs. 2019 Jun 15;37(3):473–81.

88. Maraveyas A, Waters J, Roy R, Fyfe D, Propper D, Lofts F, et al. Gemcitabine versus gemcitabine plus dalteparin thromboprophylaxis in pancreatic cancer. Eur J Cancer. 2012 Jun;48(9):1283–92.

89. Mier-Hicks A, Raj M, Do RK, Yu KH, Lowery MA, Varghese A, et al. Incidence, Management, and Implications of Visceral Thrombosis in Pancreatic Ductal Adenocarcinoma. Clin Colorectal Cancer. 2018 Jun;17(2):121–8.

90. Petrillo A, Pappalardo A, Calabrese F, Tirino G, Pompella L, Ventriglia J, et al. First line nab-paclitaxel plus gemcitabine in elderly metastatic pancreatic patients: a good choice beyond age. J Gastrointest Oncol. 2019 Oct;10(5):910–7.

91. Suleman A, Jarvis V, Hadziomerovic A, Carrier M, McDiarmid S. Implanted vascular access device related deep vein thrombosis in oncology patients: A prospective cohort study. Thromb Res. 2019 May;177:117–21.

92. Ay C, Posch F, Kaider A, Zielinski C, Pabinger I. Estimating risk of venous thromboembolism in patients with cancer in the presence of competing mortality. Journal of Thrombosis and Haemostasis. 2015 Mar;13(3):390–7.

93. Dittrich C, Königsberg R, Mittlböck M, Geissler K, Sahmanovic-Hrgovcic A, Pleiner-Duxneuner J, et al. Phase Ib trial combining capecitabine, erlotinib and bevacizumab in pancreatic adenocarcinoma - REBECA trial. Invest New Drugs. 2019 Feb 15;37(1):127–38.

94. Sharon CE, Thaler AS, Straker RJ, Kelz RR, Raper SE, Vollmer CM, et al. Fourteen years of pancreatic surgery for malignancy among ACS-NSQIP centers: Trends in major morbidity and mortality. Surgery. 2022 Aug;172(2):708–14.

95. Frere C, Bournet B, Gourgou S, Fraisse J, Canivet C, Connors JM, et al. Incidence of Venous Thromboembolism in Patients With Newly Diagnosed Pancreatic Cancer and Factors Associated With Outcomes. Gastroenterology. 2020 Apr;158(5):1346-1358.e4.

96. Lin C, Verma V, Lazenby A, Ly QP, Berim LD, Schwarz JK, et al. Phase I/II Trial of Neoadjuvant Oregovomab-based Chemoimmunotherapy Followed by Stereotactic Body Radiotherapy and Nelfinavir For Locally Advanced Pancreatic Adenocarcinoma. Am J Clin Oncol. 2019 Oct;42(10):755–60.

97. Cardin DB, Goff L, Li C, Shyr Y, Winkler C, DeVore R, et al. Phase II trial of sorafenib and erlotinib in advanced pancreatic cancer. Cancer Med. 2014 Jun 12;3(3):572–9.

98. Zhen DB, Griffith KA, Ruch JM, Camphausen K, Savage JE, Kim EJ, et al. A phase I trial of cabozantinib and gemcitabine in advanced pancreatic cancer. Invest New Drugs. 2016 Dec 21;34(6):733–9.

99. Li D, Pant S, Ryan DP, Laheru D, Bahary N, Dragovich T, et al. A phase II, open-label, multicenter study to evaluate the antitumor efficacy of CO-1.01 as second-line therapy for gemcitabine-refractory patients with stage IV pancreatic adenocarcinoma and negative tumor hENT1 expression. Pancreatology. 2014 Sep;14(5):398–402.

100. Assenat E, Mineur L, Mollevi C, Lopez-Crapez E, Lombard-Bohas C, Samalin E, et al. Phase <scp>II</scp> study evaluating the association of gemcitabine, trastuzumab and erlotinib as first-line treatment in patients with metastatic pancreatic adenocarcinoma (<scp>GATE</scp> 1). Int J Cancer. 2021 Feb 2;148(3):682–91.

101. Martín-Martos et al. Gender differences in patients with venous thromboembolism and five common sites of cancer. Thrombosis Research. 2017;S16–20.

102. Moik F, Prager G, Thaler J, Posch F, Wiedemann S, Schramm T, et al. Hemostatic Biomarkers and Venous Thromboembolism Are Associated With Mortality and Response to Chemotherapy in Patients With Pancreatic Cancer. Arterioscler Thromb Vasc Biol. 2021 Nov;41(11):2837–47.

103. Ullenhag GJ, Rossmann E, Liljefors M. A Phase I Dose-Escalation Study of Lenalidomide in Combination with Gemcitabine in Patients with Advanced Pancreatic Cancer. PLoS One. 2015 Apr 2;10(4):e0121197.

104. Beatty GL, Torigian DA, Chiorean EG, Saboury B, Brothers A, Alavi A, et al. A Phase I Study of an Agonist CD40 Monoclonal Antibody (CP-870,893) in Combination with Gemcitabine in Patients with Advanced Pancreatic Ductal Adenocarcinoma. Clinical Cancer Research. 2013 Nov 15;19(22):6286–95.

105. Middleton G, Silcocks P, Cox T, Valle J, Wadsley J, Propper D, et al. Gemcitabine and capecitabine with or without telomerase peptide vaccine GV1001 in patients with locally advanced or metastatic pancreatic cancer (TeloVac): an open-label, randomised, phase 3 trial. Lancet Oncol. 2014 Jul;15(8):829–40.

106. Tuinmann G, Hegewisch-Becker S, Zschaber R, Kehr A, Schulz J, Hossfeld DK. Gemcitabine and mitomycin C in advanced pancreatic cancer: a single-institution experience. Anticancer Drugs. 2004 Jul;15(6):575–9.

107. Van Buren G, Ramanathan RK, Krasinskas AM, Smith RP, Abood GJ, Bahary N, et al. Phase II Study of Induction Fixed-Dose Rate Gemcitabine and Bevacizumab Followed by 30 Gy Radiotherapy as Preoperative Treatment for Potentially Resectable Pancreatic Adenocarcinoma. Ann Surg Oncol. 2013 Nov 1;20(12):3787–93.

108. Chew HK, Wun T, Harvey D, Zhou H, White RH. Incidence of Venous Thromboembolism and Its Effect on Survival Among Patients With Common Cancers. Arch Intern Med. 2006 Feb 27;166(4):458.

109. Kindler HL, Niedzwiecki D, Hollis D, Sutherland S, Schrag D, Hurwitz H, et al. Gemcitabine Plus Bevacizumab Compared With Gemcitabine Plus Placebo in Patients With Advanced Pancreatic Cancer: Phase III Trial of the Cancer and Leukemia Group B (CALGB 80303). Journal of Clinical Oncology. 2010 Aug 1;28(22):3617–22.

110. Yhim HY, Jang MJ, Kwak JY, Yim CY, Choi WI, Lee YC, et al. The incidence, risk factors, and prognosis of recurrent venous thromboembolism (VTE) in patients with advanced solid cancers receiving anticoagulation therapy after the diagnosis of index VTE. Thromb Res. 2013 Apr;131(4):e133–40.

111. Astsaturov IA, Meropol NJ, Alpaugh RK, Burtness BA, Cheng JD, McLaughlin S, et al. Phase II and Coagulation Cascade Biomarker Study of Bevacizumab With or Without Docetaxel in Patients With Previously Treated Metastatic Pancreatic Adenocarcinoma. Am J Clin Oncol. 2011 Feb;34(1):70–5.

112. Chau I, Cunningham D, Russell C, Norman AR, Kurzawinski T, Harper P, et al. Gastrazole (JB95008), a novel CCK2/gastrin receptor antagonist, in the treatment of advanced pancreatic cancer: results from two randomised controlled trials. Br J Cancer. 2006 Apr 4;94(8):1107–15.

113. Gade IL, Brækkan SK, Næss IA, Hansen J -B., Cannegieter SC, Overvad K, et al. The impact of initial cancer stage on the incidence of venous thromboembolism: the Scandinavian Thrombosis and Cancer (STAC) Cohort. Journal of Thrombosis and Haemostasis. 2017 Aug;15(8):1567–75.

114. Aparicio J, García-Mora C, Martín M, Petriz ML, Feliu J, Sánchez-Santos ME, et al. A Phase I, Dose-Finding Study of Sorafenib in Combination with Gemcitabine and Radiation Therapy in Patients with Unresectable Pancreatic Adenocarcinoma: A Grupo Español Multidisciplinario en Cáncer Digestivo (GEMCAD) Study. PLoS One. 2014 Jan 9;9(1):e82209.

115. Bendell J, O'Reilly EM, Middleton MR, Chau I, Hochster H, Fielding A, et al. Phase I study of olaparib plus gemcitabine in patients with advanced solid tumours and comparison with gemcitabine alone in patients with locally advanced/metastatic pancreatic cancer. Annals of Oncology. 2015 Apr;26(4):804–11.

116. Hoffman JP, Lipsitz S, Pisansky T, Weese JL, Solin L, Benson AB. Phase II trial of preoperative radiation therapy and chemotherapy for patients with localized, resectable adenocarcinoma of the pancreas: an Eastern Cooperative Oncology Group Study. Journal of Clinical Oncology. 1998 Jan;16(1):317–23.

117. Spano JP, Moore MJ, Pithavala YK, Ricart AD, Kim S, Rixe O. Phase I study of axitinib (AG-013736) in combination with gemcitabine in patients with advanced pancreatic cancer. Invest New Drugs. 2012 Aug 14;30(4):1531–9.

118. Infante JR, Jones SF, Bendell JC, Spigel DR, Yardley DA, Weekes CD, et al. A phase I, dose-escalation study of pomalidomide (CC-4047) in combination with gemcitabine in metastatic pancreas cancer. Eur J Cancer. 2011 Jan;47(2):199–205.

119. Kim JS, Kang EJ, Kim DS, Choi YJ, Lee SY, Kim HJ, et al. Early venous thromboembolism at the beginning of palliative chemotherapy is a poor prognostic factor in patients with metastatic pancreatic cancer: a retrospective study. BMC Cancer. 2018 Dec 17;18(1):1260.

120. Blom JW, Osanto S, Rosendaal FR. High risk of venous thrombosis in patients with pancreatic cancer: A cohort study of 202 patients. Eur J Cancer. 2006 Feb;42(3):410–4.

121. Rad MA, Fanipakdel A, Vossoughinia H, Dadgarmoghaddam M, Esmaeili B, Jafarian AH, et al. Demographic and Clinical Evaluation of Patients with Pancreatic Cancer in Mashhad University of Medical Sciences During 2002-2013. Curr Cancer Ther Rev. 2020 Jun 9;16(2):127–35.

122. Barrau M, Maoui K, Le Roy B, Roblin X, Mismetti P, Phelip JM, et al. Early venous thromboembolism is a strong prognostic factor in patients with advanced pancreatic ductal adenocarcinoma. J Cancer Res Clin Oncol. 2021 Nov 14;147(11):3447–54.

123. LAMBERT M, PLOQUIN A, DECLERCK L, DUHAMEL A, MAKHLOUFI S, TURPIN A, et al. Deep Vein Thrombosis: An Independent Poor Prognosis Factor of Advanced Pancreatic Adenocarcinoma. Anticancer Res. 2016 Oct 12;36(10):5527–30.

124. Mita MM, Nemunaitis J, Grilley-Olson J, El-Rayes B, Bekaii-Saab T, Harvey RD, et al. Phase 1 Study of CEP-37250/KHK2804, a Tumor-specific Anti-glycoconjugate Monoclonal Antibody, in Patients with Advanced Solid Tumors. Target Oncol. 2016 Dec 25;11(6):807–14.

125. Ouaïssi M, Frasconi C, Mege D, Panicot-dubois L, Boiron L, Dahan L, et al. Impact of venous thromboembolism on the natural history of pancreatic adenocarcinoma. Hepatobiliary & Pancreatic Diseases International. 2015 Aug;14(4):436–42.

126. Cardillo N, Seible DM, Fero KE, Bruggeman AR, Sarkar RR, Azuara A, et al. Clinical Impact of Local Progression in Pancreatic Cancer. Journal of the National Comprehensive Cancer Network. 2018 Jun 11;16(6):711–7.

127. Khalaf N, Kramer J, Liu Y, Abrams D, Singh H, El-Serag H, et al. Diabetes Status and Pancreatic Cancer Survival in the Nationwide Veterans Affairs Healthcare System. Dig Dis Sci. 2023 Sep 20;68(9):3634–43.

128. Nso N, Nyabera A, Nassar M, Mbome Y, Emmanuel K, Alshamam M, et al. Incidence and risk factors of cardiovascular mortality in patients with gastrointestinal adenocarcinoma. PLoS One. 2023 Jan 27;18(1):e0262013.

129. Starling N, Watkins D, Cunningham D, Thomas J, Webb J, Brown G, et al. Dose Finding and Early Efficacy Study of Gemcitabine Plus Capecitabine in Combination With Bevacizumab Plus Erlotinib in Advanced Pancreatic Cancer. Journal of Clinical Oncology. 2009 Nov 20;27(33):5499–505.

130. Chan PC, Chang WL, Hsu MH, Yeh CH, Muo CH, Chang KS, et al. Higher stroke incidence in the patients with pancreatic cancer. Medicine. 2018 Mar;97(11):e0133.

131. Stein PD, Beemath A, Meyers FA, Skaf E, Sanchez J, Olson RE. Incidence of Venous Thromboembolism in Patients Hospitalized with Cancer. Am J Med. 2006 Jan;119(1):60–8.

132. Eijgenraam P, Heinen MM, Verhage BAJ, Keulemans YC, Schouten LJ, van den Brandt PA. Diabetes type II, other medical conditions and pancreatic cancer risk: a prospective study in The Netherlands. Br J Cancer. 2013 Nov 22;109(11):2924–32.

133. Ghaneh P, Palmer D, Cicconi S, Jackson R, Halloran CM, Rawcliffe C, et al. Immediate surgery compared with short-course neoadjuvant gemcitabine plus capecitabine, FOLFIRINOX, or chemoradiotherapy in patients with borderline resectable pancreatic cancer (ESPAC5): a four-arm, multicentre, randomised, phase 2 trial. Lancet Gastroenterol Hepatol. 2023 Feb;8(2):157–68.

134. Grierson P, Teague A, Suresh R, Lim KH, Amin M, Pedersen K, et al. Phase Ib/II study combining tosedostat with capecitabine in patients with advanced pancreatic adenocarcinoma. J Gastrointest Oncol. 2020 Feb;11(1):61–7.

135. Han P, Yang Y, He Y, Wu H, Wang D, Liu K, et al. The effect of atrial fibrillation on perioperative outcomes in patients with pancreatic cancer undergoing open pancreaticoduodenectomy: analysis of the National Inpatient Sample. Gland Surg. 2021 Jun;10(6):1951–61.

136. Hanna-Sawires RG, Groen J V., Hamming A, Tollenaar RAEM, Mesker WE, Luelmo SAC, et al. Incidence, timing and risk factors of venous thromboembolic events in patients with pancreatic cancer. Thromb Res. 2021 Nov;207:134–9.

137. Ramanathan RK, Thomas GW, Khorana AA, Shah S, Zhou C, Wong S, et al. A Phase 2 Study of PCI-27483, a Factor VIIa Inhibitor in Combination with Gemcitabine for Advanced Pancreatic Cancer. Oncology. 2019;96(4):217–22.

138. García Adrián S, González AR, de Castro EM, Olmos VP, Morán LO, del Prado PM, et al. Incidence, risk factors, and evolution of venous thromboembolic events in patients diagnosed with pancreatic carcinoma and treated with chemotherapy on an outpatient basis. Eur J Intern Med. 2022 Nov;105:30–7.

139. Kondo S, Sasaki M, Hosoi H, Sakamoto Y, Morizane C, Ueno H, et al. Incidence and risk factors for venous thromboembolism in patients with pretreated advanced pancreatic carcinoma. Oncotarget. 2018 Mar 30;9(24):16883–90.

140. Kruger S, Haas M, Burkl C, Goehring P, Kleespies A, Roeder F, et al. Incidence, outcome and risk stratification tools for venous thromboembolism in advanced pancreatic cancer – A retrospective cohort study. Thromb Res. 2017 Sep;157:9–15.

141. Hingorani SR, Zheng L, Bullock AJ, Seery TE, Harris WP, Sigal DS, et al. HALO 202: Randomized Phase II Study of PEGPH20 Plus Nab-Paclitaxel/Gemcitabine Versus Nab-Paclitaxel/Gemcitabine in Patients With Untreated, Metastatic Pancreatic Ductal Adenocarcinoma. Journal of Clinical Oncology. 2018 Feb 1;36(4):359–66.

142. Yoon SY, Lee MY, Yoon J, Kim HJ, Kim KH, Kim SH, et al. The incidence of venous thromboembolism is not lowin Korean patients with advanced pancreatic cancer. Blood Res. 2018;53(3):227.

143. Dragovich T, Laheru D, Dayyani F, Bolejack V, Smith L, Seng J, et al. Phase II trial of vatalanib in patients with advanced or metastatic pancreatic adenocarcinoma after first-line gemcitabine therapy (PCRT O4-001). Cancer Chemother Pharmacol. 2014 Aug 18;74(2):379–87.

144. Okusaka T, Saiura A, Shimada K, Ikeda M, Ioka T, Kimura T, et al. Incidence and risk factors for venous thromboembolism in the Cancer-VTE Registry pancreatic cancer subcohort. J Gastroenterol. 2023 Dec 7;58(12):1261–71.

145. Chew TW, Gau CS, Wen YW, Shen LJ, Mullins CD, Hsiao FY. Epidemiology, clinical profile and treatment patterns of venous thromboembolism in cancer patients in Taiwan: a population-based study. BMC Cancer. 2015 Dec 17;15(1):298.

146. Yamai T, Ikezawa K, Hiraga E, Kawamoto Y, Hirao T, Higashi S, et al. Early detection of venous thromboembolism after the initiation of chemotherapy predicts a poor prognosis in patients with unresectable metastatic pancreatic cancer who underwent first-line chemotherapy with gemcitabine plus nab-paclitaxel. PLoS One. 2022 Mar 1;17(3):e0264653.

147. Nakai Y, Isayama H, Ijichi H, Sasaki T, Kogure H, Yagioka H, et al. Phase I trial of gemcitabine and candesartan combination therapy in normotensive patients with advanced pancreatic cancer: <scp>GECA</scp> 1. Cancer Sci. 2012 Aug 15;103(8):1489–92.

148. Nakai Y, Isayama H, Sasaki T, Mizuno S, Sasahira N, Kogure H, et al. Clinical Outcomes of Chemotherapy for Diabetic and Nondiabetic Patients With Pancreatic Cancer. Pancreas. 2013 Mar;42(2):202–8.

149. Hung YS, Chen JS, Chen YY, Lu CH, Chang PH, Chou WC. Incidence, Risk Factors, and Outcomes of Arterial Thromboembolism in Patients with Pancreatic Cancer Following Palliative Chemotherapy. Cancers (Basel). 2018 Nov 12;10(11):432.

150. Nagy Z, Horváth O, Kádas J, Valtinyi D, László L, Kopper B, et al. D-Dimer as a Potential Prognostic Marker. Pathology & Oncology Research. 2012 Jul 28;18(3):669–74.

151. Schwartzberg LS, Arena FP, Bienvenu BJ, Kaplan EH, Camacho LH, Campos LT, et al. A Randomized, Open-Label, Safety and Exploratory Efficacy Study of Kanglaite Injection (KLTi) plus Gemcitabine versus Gemcitabine in Patients with Advanced Pancreatic Cancer. J Cancer. 2017;8(10):1872–83.

152. Riedl J, Posch F, Königsbrügge O, Lötsch F, Reitter EM, Eigenbauer E, et al. Red Cell Distribution Width and Other Red Blood Cell Parameters in Patients with Cancer: Association with Risk of Venous Thromboembolism and Mortality. PLoS One. 2014 Oct 27;9(10):e111440.

153. Infante J, Arkenau HT, Bendell J, Rubin M, Waterhouse D, Jones G, et al. Lenalidomide in combination with gemcitabine as first-line treatment for patients with metastatic carcinoma of the pancreas: A Sarah Cannon Research Institute phase II trial. Cancer Biol Ther. 2013 Apr 27;14(4):340–6.

154. Ko AH, LoConte N, Tempero MA, Walker EJ, Kate Kelley R, Lewis S, et al. A Phase I Study of FOLFIRINOX Plus IPI-926, a Hedgehog Pathway Inhibitor, for Advanced Pancreatic Adenocarcinoma. Pancreas. 2016 Mar;45(3):370–5.

155. Picozzi VJ, Ramanathan RK, Lowery MA, Ocean AJ, Mitchel EP, O'Neil BH, et al. 90 Y-clivatuzumab tetraxetan with or without low-dose gemcitabine: A phase Ib study in patients with metastatic pancreatic cancer after two or more prior therapies. Eur J Cancer. 2015 Sep;51(14):1857–64.

156. Herman JM, Swartz MJ, Hsu CC, Winter J, Pawlik TM, Sugar E, et al. Analysis of Fluorouracil-Based Adjuvant Chemotherapy and Radiation After Pancreaticoduodenectomy for Ductal Adenocarcinoma of the Pancreas: Results of a Large, Prospectively Collected Database at the Johns Hopkins Hospital. Journal of Clinical Oncology. 2008 Jul 20;26(21):3503–10.

157. Epstein AS, Soff GA, Capanu M, Crosbie C, Shah MA, Kelsen DP, et al. Analysis of incidence and clinical outcomes in patients with thromboembolic events and invasive exocrine pancreatic cancer. Cancer. 2012 Jun 15;118(12):3053–61.

158. O'Reilly EM, Niedzwiecki D, Hall M, Hollis D, Bekaii-Saab T, Pluard T, et al. A Cancer and Leukemia Group B Phase II Study of Sunitinib Malate in Patients with Previously Treated Metastatic Pancreatic Adenocarcinoma (CALGB 80603). Oncologist. 2010 Dec 1;15(12):1310–9.

159. Xiong HQ, Rosenberg A, LoBuglio A, Schmidt W, Wolff RA, Deutsch J, et al. Cetuximab, a Monoclonal Antibody Targeting the Epidermal Growth Factor Receptor, in Combination With Gemcitabine for Advanced Pancreatic Cancer: A Multicenter Phase II Trial. Journal of Clinical Oncology. 2004 Jul 1;22(13):2610–6.

160. Spanheimer PM, Cyr AR, Liao J, Johlin FC, Hoshi H, Howe JR, et al. Complications and survival associated with operative procedures in patients with unresectable pancreatic head adenocarcinoma. J Surg Oncol. 2014 Jun 7;109(7):697–701.

161. Spano JP, Chodkiewicz C, Maurel J, Wong R, Wasan H, Barone C, et al. Efficacy of gemcitabine plus axitinib compared with gemcitabine alone in patients with advanced pancreatic cancer: an open-label randomised phase II study. The Lancet. 2008 Jun;371(9630):2101–8.

162. Pelzer U, Opitz B, Deutschinoff G, Stauch M, Reitzig PC, Hahnfeld S, et al. Efficacy of Prophylactic Low–Molecular Weight Heparin for Ambulatory Patients With Advanced Pancreatic Cancer: Outcomes From the CONKO-004 Trial. Journal of Clinical Oncology. 2015 Jun 20;33(18):2028–34.

163. Pandit H, Hong YK, Li Y, Rostas J, Pulliam Z, Li SP, et al. Evaluating the Regulatory Immunomodulation Effect of Irreversible Electroporation (IRE) in Pancreatic Adenocarcinoma. Ann Surg Oncol. 2019 Mar 4;26(3):800–6.

164. Müller MW, Friess H, Köninger J, Martin D, Wente MN, Hinz U, et al. Factors influencing survival after bypass procedures in patients with advanced pancreatic adenocarcinomas. The American Journal of Surgery. 2008 Feb;195(2):221–8.

165. Faris JE, Blaszkowsky LS, McDermott S, Guimaraes AR, Szymonifka J, Huynh MA, et al. FOLFIRINOX in Locally Advanced Pancreatic Cancer: The Massachusetts General Hospital Cancer Center Experience. Oncologist. 2013 May 1;18(5):543–8.

166. Conroy T, Hammel P, Hebbar M, Ben Abdelghani M, Wei AC, Raoul JL, et al. FOLFIRINOX or Gemcitabine as Adjuvant Therapy for Pancreatic Cancer. New England Journal of Medicine. 2018 Dec 20;379(25):2395–406.

167. Conroy T, Desseigne F, Ychou M, Bouché O, Guimbaud R, Bécouarn Y, et al. FOLFIRINOX versus Gemcitabine for Metastatic Pancreatic Cancer. New England Journal of Medicine. 2011 May 12;364(19):1817–25.

168. Loehrer PJ, Feng Y, Cardenes H, Wagner L, Brell JM, Cella D, et al. Gemcitabine Alone Versus Gemcitabine Plus Radiotherapy in Patients With Locally Advanced Pancreatic Cancer: An Eastern Cooperative Oncology Group Trial. Journal of Clinical Oncology. 2011 Nov 1;29(31):4105–12.

169. Heinemann V, Wilke H, Mergenthaler HG, Clemens M, König H, Illiger HJ, et al. Gemcitabine and cisplatin in the treatment of advanced or metastatic pancreatic cancer. Annals of Oncology. 2000 Nov;11(11):1399–403.

170. Kindler HL, Wroblewski K, Wallace JA, Hall MJ, Locker G, Nattam S, et al. Gemcitabine plus sorafenib in patients with advanced pancreatic cancer: a phase II trial of the University of Chicago Phase II Consortium. Invest New Drugs. 2012 Feb 28;30(1):382–6.

171. Haddock MG, Swaminathan R, Foster NR, Hauge MD, Martenson JA, Camoriano JK, et al. Gemcitabine, Cisplatin, and Radiotherapy for Patients With Locally Advanced Pancreatic Adenocarcinoma: Results of the North Central Cancer Treatment Group Phase II Study N9942. Journal of Clinical Oncology. 2007 Jun 20;25(18):2567–72.

172. Burris HA, Moore MJ, Andersen J, Green MR, Rothenberg ML, Modiano MR, et al. Improvements in survival and clinical benefit with gemcitabine as first-line therapy for patients with advanced pancreas cancer: a randomized trial. Journal of Clinical Oncology. 1997 Jun;15(6):2403–13.

173. Chou SC, Pai CH, Lin SW, Tien HF. Incidence and risk factors for venous thromboembolism in a cohort of Taiwanese patients with lung, gastric, pancreatic cancers or lymphoma. Journal of the Formosan Medical Association. 2022 Jan;121(1):360–6.

174. Martin RC, Philips P, Ellis S, Hayes D, Bagla S. Irreversible electroporation of unresectable soft tissue tumors with vascular invasion: effective palliation. BMC Cancer. 2014 Dec 26;14(1):540.

175. Vogel JA, Rombouts SJ, de Rooij T, van Delden OM, Dijkgraaf MG, van Gulik TM, et al. Induction Chemotherapy Followed by Resection or Irreversible Electroporation in Locally Advanced Pancreatic Cancer (IMPALA): A Prospective Cohort Study. Ann Surg Oncol. 2017 Sep 30;24(9):2734–43.

176. Rocha Lima CM, Green MR, Rotche R, Miller WH, Jeffrey GM, Cisar LA, et al. Irinotecan Plus Gemcitabine Results in No Survival Advantage Compared With Gemcitabine Monotherapy in Patients With Locally Advanced or Metastatic Pancreatic Cancer Despite Increased Tumor Response Rate. Journal of Clinical Oncology. 2004 Sep 15;22(18):3776–83.

177. Icli F, Akbulut H, Utkan G, Yalcin B, Dincol D, Isikdogan A, et al. Low molecular weight heparin (LMWH) increases the efficacy of cisplatinum plus gemcitabine combination in advanced pancreatic cancer. J Surg Oncol. 2007 May 27;95(6):507–12.

178. Reni M, Cereda S, Milella M, Novarino A, Passardi A, Mambrini A, et al. Maintenance sunitinib or observation in metastatic pancreatic adenocarcinoma: A phase II randomised trial. Eur J Cancer. 2013 Nov;49(17):3609–15.

179. Kim EJ, Ben-Josef E, Herman JM, Bekaii-Saab T, Dawson LA, Griffith KA, et al. A multi-institutional phase 2 study of neoadjuvant gemcitabine and oxaliplatin with radiation therapy in patients with pancreatic cancer. Cancer. 2013 Aug 29;119(15):2692–700.

180. Philip PA, Lacy J, Portales F, Sobrero A, Pazo-Cid R, Manzano Mozo JL, et al. Nab-paclitaxel plus gemcitabine in patients with locally advanced pancreatic cancer (LAPACT): a multicentre, open-label phase 2 study. Lancet Gastroenterol Hepatol. 2020 Mar;5(3):285–94.

181. Miyamoto DT, Mamon HJ, Ryan DP, Willett CG, Ancukiewicz M, Kobayashi WK, et al. Outcomes and Tolerability of Chemoradiation Therapy for Pancreatic Cancer Patients Aged 75 Years or Older. International Journal of Radiation Oncology*Biology*Physics. 2010 Jul;77(4):1171–7.

182. Safran H, Moore T, Iannitti D, Dipetrillo T, Akerman P, Cioffi W, et al. Paclitaxel and concurrent radiation for locally advanced pancreatic cancer. International Journal of Radiation Oncology*Biology*Physics. 2001 Apr;49(5):1275–9.

183. Mamon HJ, Niedzwiecki D, Hollis D, Tan BR, Mayer RJ, Tepper JE, et al. A phase 2 trial of gemcitabine, 5-fluorouracil, and radiation therapy in locally advanced nonmetastatic pancreatic adenocarcinoma. Cancer. 2011 Jun 15;117(12):2620–8.

184. Ko AH, Youssoufian H, Gurtler J, Dicke K, Kayaleh O, Lenz HJ, et al. A phase II randomized study of cetuximab and bevacizumab alone or in combination with gemcitabine as first-line therapy for metastatic pancreatic adenocarcinoma. Invest New Drugs. 2012 Aug 1;30(4):1597–606.

185. Ko AH, Venook AP, Bergsland EK, Kelley RK, Korn WM, Dito E, et al. A phase II study of bevacizumab plus erlotinib for gemcitabine-refractory metastatic pancreatic cancer. Cancer Chemother Pharmacol. 2010 Nov 4;66(6):1051–7.

186. Rich T, Harris J, Abrams R, Erickson B, Doherty M, Paradelo J, et al. Phase II Study of External Irradiation and Weekly Paclitaxel for Nonmetastatic, Unresectable Pancreatic Cancer: RTOG-98-12. Am J Clin Oncol. 2004 Feb;27(1):51–6.

187. Small W, Mulcahy MF, Rademaker A, Bentrem DJ, Benson AB, Weitner BB, et al. Phase II Trial of Full-Dose Gemcitabine and Bevacizumab in Combination With Attenuated Three-Dimensional Conformal Radiotherapy in Patients With Localized Pancreatic Cancer. International Journal of Radiation Oncology*Biology*Physics. 2011 Jun;80(2):476–82.

188. Van Cutsem E, Vervenne WL, Bennouna J, Humblet Y, Gill S, Van Laethem JL, et al. Phase III Trial of Bevacizumab in Combination With Gemcitabine and Erlotinib in Patients With Metastatic Pancreatic Cancer. Journal of Clinical Oncology. 2009 May 1;27(13):2231–7.

189. Van Cutsem E, van de Velde H, Karasek P, Oettle H, Vervenne WL, Szawlowski A, et al. Phase III Trial of Gemcitabine Plus Tipifarnib Compared With Gemcitabine Plus Placebo in Advanced Pancreatic Cancer. Journal of Clinical Oncology. 2004 Apr 15;22(8):1430–8.

190. Poplin E, Feng Y, Berlin J, Rothenberg ML, Hochster H, Mitchell E, et al. Phase III, Randomized Study of Gemcitabine and Oxaliplatin Versus Gemcitabine (fixed-dose rate infusion) Compared With Gemcitabine (30-minute infusion) in Patients With Pancreatic Carcinoma E6201: A Trial of the Eastern Cooperative Oncology Group. Journal of Clinical Oncology. 2009 Aug 10;27(23):3778–85.

191. Pisters PWT, Hudec WA, Lee JE, Raijman I, Lahoti S, Janjan NA, et al. Preoperative Chemoradiation for Patients With Pancreatic Cancer: Toxicity of Endobiliary Stents. Journal of Clinical Oncology. 2000 Feb 14;18(4):860–860.

192. Varadhachary GR, Wolff RA, Crane CH, Sun CC, Lee JE, Pisters PWT, et al. Preoperative Gemcitabine and Cisplatin Followed by Gemcitabine-Based Chemoradiation for Resectable Adenocarcinoma of the Pancreatic Head. Journal of Clinical Oncology. 2008 Jul 20;26(21):3487–95.

193. Evans DB, Varadhachary GR, Crane CH, Sun CC, Lee JE, Pisters PWT, et al. Preoperative Gemcitabine-Based Chemoradiation for Patients With Resectable Adenocarcinoma of the Pancreatic Head. Journal of Clinical Oncology. 2008 Jul 20;26(21):3496–502.

194. Reni M, Zanon S, Balzano G, Passoni P, Pircher C, Chiaravalli M, et al. A randomised phase 2 trial of nab-paclitaxel plus gemcitabine with or without capecitabine and cisplatin in locally advanced or borderline resectable pancreatic adenocarcinoma. Eur J Cancer. 2018 Oct;102:95–102.

195. Colucci G, Labianca R, Di Costanzo F, Gebbia V, Cartenì G, Massidda B, et al. Randomized Phase III Trial of Gemcitabine Plus Cisplatin Compared With Single-Agent Gemcitabine As First-Line Treatment of Patients With Advanced Pancreatic Cancer: The GIP-1 Study. Journal of Clinical Oncology. 2010 Apr 1;28(10):1645–51.

196. Hurwitz HI, Uppal N, Wagner SA, Bendell JC, Beck JT, Wade SM, et al. Randomized, Double-Blind, Phase II Study of Ruxolitinib or Placebo in Combination With Capecitabine in Patients With Metastatic Pancreatic Cancer for Whom Therapy With Gemcitabine Has Failed. Journal of Clinical Oncology. 2015 Dec 1;33(34):4039–47.

197. Martin RCG, Kwon D, Chalikonda S, Sellers M, Kotz E, Scoggins C, et al. Treatment of 200 Locally Advanced (Stage III) Pancreatic Adenocarcinoma Patients With Irreversible Electroporation. Ann Surg. 2015 Sep;262(3):486–94.
